# Pattern of forest recovery and carbon stock following shifting cultivation in Manipur, North-East India

**DOI:** 10.1371/journal.pone.0239906

**Published:** 2020-10-08

**Authors:** Pentile Thong, Uttam Kumar Sahoo, Uttam Thangjam, Rocky Pebam

**Affiliations:** 1 Department of Forestry, Mizoram University, Aizawl, Mizoram, India; 2 North-Eastern Space Applications Centre, Umiam, Meghalaya, India; Institut Agro - AGROCAMPUS OUEST, FRANCE

## Abstract

Shifting cultivation has resulted in large-scale deforestation and forest degradation in the tropics; however the abandoned fallows are known to have high potential for carbon capture. The paper is an attempt to determine the forest recovery patterns following shifting cultivation by evaluating the tree species composition, diversity and abundance with respect to topographical factors in Manipur, India. We also used ordination analysis to understand the change in species composition with regard to environmental variables. The living woody biomass carbon of each fallow was quantified, and the factors affecting the recovery of carbon stock along an increasing fallow gradient was assessed. Our results showed that the species richness and basal area recovered relatively with time since abandonment, and the north-facing lower elevation fallow sites displayed higher species richness and stem density than those in higher elevations. Environmental variables had no impact on the regeneration of *Elaeocarpus floribundus* Blume and *Castanopsis hystrix* Hook. f. & Thomson ex A. DC. which suggests that they may be capable of effective restoration of degraded forest areas. As these species appear naturally in the forests, it would facilitate quicker rehabilitation and reinstate the soil nutrients making the soil reusable in a short term. We also found that fallow age plays a vital role in recovering above-ground biomass carbon from living woody species followed by the aspect of the site. The total living woody biomass carbon ranged from 0.98 Mg ha^-1^ in 5 years fallow to 142.58 Mg ha^-1^ in 20 years fallow. The above-ground biomass carbon recovery of the oldest fallow was 39% to 40% of the reference undisturbed forest and the estimated time for the shifting cultivation fallows to reach that of the undisturbed forest level was approximately 39 years to 41 years.

## Introduction

Shifting cultivation is a major land use in tropics despite the fact that it is blamed to be a major cause of forest loss. Serious concerns are raised that this practice though the oldest form of agriculture may negatively affect biodiversity, carbon stocks and greenhouse gas emissions [1–3]. However, this practice provides subsistence livelihoods to millions of people worldwide [1] and, therefore, it is likely to continue as it is intricately linked to cultural, ecological and economic aspects of communities [4]. This practice is characterized by the alternation of the cropping and fallow phase, while the abandoned land regenerates naturally. After a certain fallow period, the trees are slashed and burnt for the ashes to enrich the soil, thus allowing a new cropping phase. This intervening period between two successive slashed and burnt practices is termed as *Jhum cycle*. The fallow duration and cropping period are specifically influenced by ecological and socio-economic factors [5]. In North-East India, this practice is locally known as Jhum where the cropping period has been reported to be 1–2 years with a fallow duration varying from 6–12 years [6, 7]. The regional estimation of abandoned land after Jhum differs significantly depending on the means of estimation. However, this region encompasses 3697.14 km^2^ of the National area of abandoned land [8]. Manipur, in particular, has 0.45% (100.10 km^2^) of its total geographical area under abandoned Jhum land. Under the natural process of ecological recovery, the abandoned lands are eventually restored to secondary forests.

In the tropical region, secondary forests constitute over 50% of the forested area and have the ability to assimilate and store carbon [9]. In India, secondary forests cover about 32 million ha which is approximately 45.8% of the forest area of the country [10]. The dynamic nature of shifting cultivation results in a landscape mosaic of Jhum field, secondary forests and old-growth forests. As such, many forests in North-East India are secondary forests at different stages of succession following shifting cultivation. These forests are an essential source of rural livelihood and also for multiple environmental functions such as soil and watershed conservation, flood control and carbon storage [11]. However, secondary forests get constantly subjected to increasing exploitation by the growing population as well as by budding industrial and urban demand for forest products. Succession following Jhum is comprised of fast growing species with high regeneration and species accumulation than other human-modified and abandoned agroforestry systems [12]. In the tropics, the diversity of woody species steadily increases with the age of fallow [13]. The quantity of biomass in a forest ascertains the potential amount of carbon added to the atmosphere or sequestered on the land [14]. An understanding of species recovery especially woody species and carbon stock is vital not only for developing rehabilitation strategy for shifting cultivation areas but also for carbon-based payments for ecosystem services such as reducing emissions from deforestation and forest degradation.

Given the importance of the secondary forests and the human pressure on these resources, the present study is conducted to (1) understand the tree recovery pattern following shifting cultivation, (2) estimate the living woody biomass and carbon stock under different fallow regimes, (3) identify naturally occurring potential tree species to accelerate the restorative phase of the fallows. Despite the undulated topography, the state of Manipur has 77.69% of its total geographical area under forest cover, based on which we put forward the hypothesis that topographical parameters namely slope, aspect and elevation have a bearing on the species composition with increasing fallow age. We believe that the result of this study will provide baseline data on the rate of vegetation recovery and biomass carbon as accumulated by the living woody species in order to achieve proper management of such successive areas.

## Materials and methods

### Ethics statement

The study was carried out in four different shifting cultivation fallow lands owned by villagers in Ukhrul and Chandel district of Manipur. Permission was obtained from each of the land owner towards collection of plant samples and vegetation data. No rare or endangered species was used in this study, and besides the study did neither involve the use of wild animals nor threatened environmental systems.

### Study area

**Fig 1:**
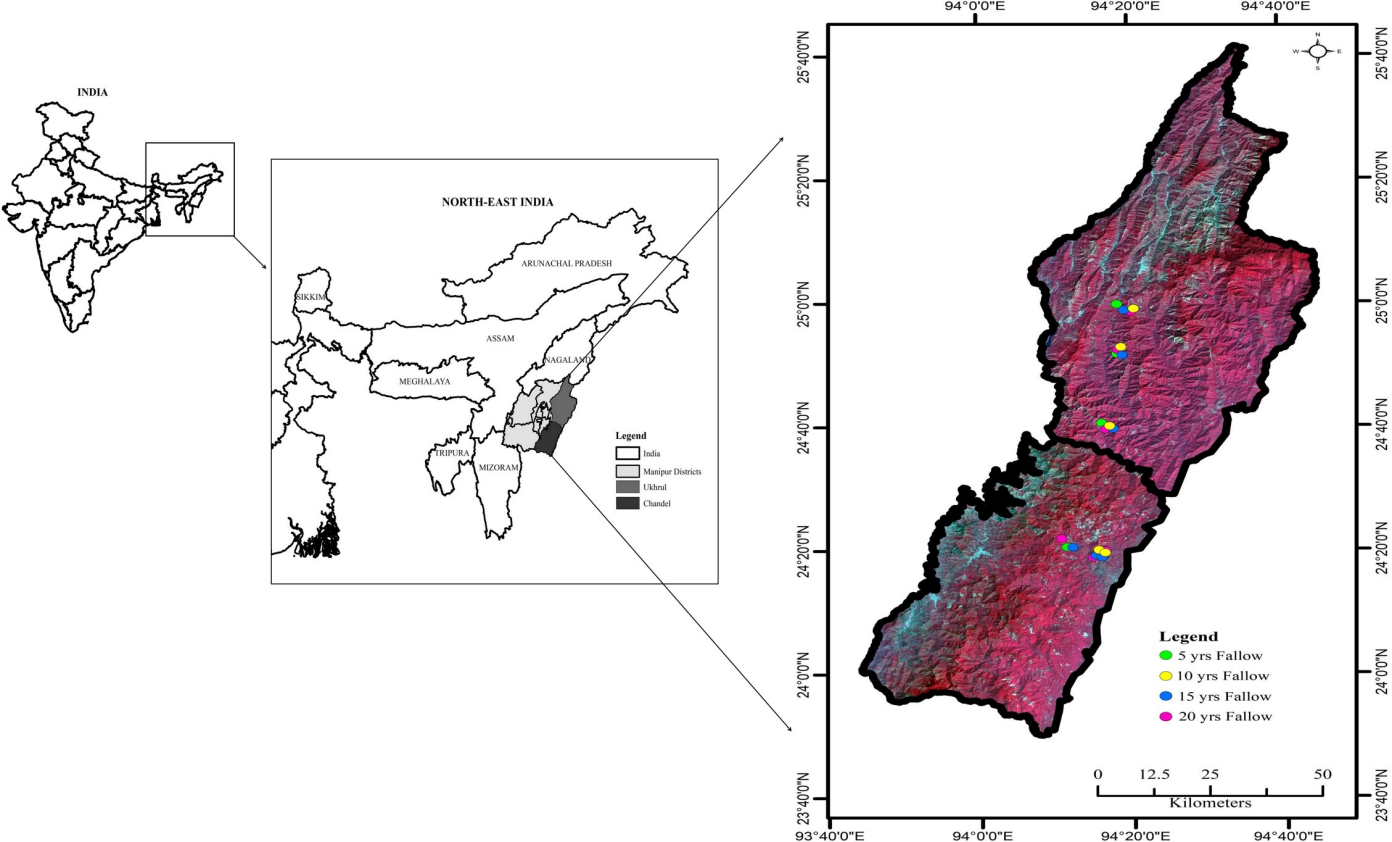
Map showing the location of the study area (source: www.glovis.usgs.gov).

The study was conducted in Ukhrul (24°29’6”-24°41’35”-N and 94°7’24”-94°44’44”E) and Chandel (23°50’11”-24°38’52”N and 93°53’58”-94°18’10”E) districts of Manipur, Northeast India. The satellite imagery for the map showing the study area was downloaded from www.glovis.usgs.gov which is a public domain (Fig 1). These districts are situated in the north-eastern and south-eastern part of the State. Ukhrul is located at an elevation of 1662 m a.s.l and Chandel at 957 m a.s.l. The subtropical climate of this region is affected by the southwest and northeast monsoons with distinct rainy (June to September) and dry (November to February) seasons. Ukhrul and Chandel districts have contributed to the increase in the net forest cover of the state by 151 km^2^ and 17 km^2^ respectively [15].

### Site selection

Keeping in view the topographical parameters, soil type, and mean annual rainfall and temperature, we selected four different fallow periods after the abandonment of the Jhum fields, namely, 5 (JF_5_), 10 (JF_10_), 15 (JF_15_) and 20 (JF_20_) years fallow (Table 1). The study sites were selected as per the findings of Thong et al. [7] and the plot was navigated using a GPS.

**Table 1:** Detail characteristics of the study sites.

	UKHRUL						CHANDEL					
Fallow age	Location	Elevation	Slope (°)	Aspect	Soil type	Avg. annual rainfall & temperature	Location	Elevation	Slope (°)	Aspect	Soil type	Avg. annual rainfall & temperature
5	24°51'48"N, 94°18'16"E	995–1215	1–41	N	Alluvial, Lateritic black and red soil	1616 mm & 15.5°C	24°20'32"N, 94°11'25"E	593–886	13–36	S	Alluvial, Lateritic and red clayey soil	1877 mm & 20.6°C
5	24°59'49"N, 94°18'17"E	1036–1152	1–37	NE			24°19'04"N, 94°15'04"E	706–769	4–38	SE		
5	24°40'36"N, 94°16'07"E	1085–1285	13–42	W			24°20'01"N, 94°16'24"E	557–590	1–34	N		
10	24°52'54"N, 94°18'47"E	1369–1453	3–32	S			24°21'07"N, 94°12'11"E	723–755	3–35	N		
10	24°59'05"N, 94°20'31"E	1179–1313	4–43	E			24°20'04"N, 94°15'35"E	398–441	11–31	SE,S		
10	24°40'08"N, 94°17'10"E	1190–1290	2–56	N			24°19'37"N, 94°16'25"E	466–566	6–29	SE		
15	24°51'35"N, 94°18'58"E	1256–1404	11–42	E			24°20'29"N, 94°12'10"E	507–650	14–38	S,SW		
15	24°58'51"N, 94°19'13"E	1216–1275	3–49	N			24°19'09"N, 94°15'14"E	645–708	15–33	E		
15	24°39'41"N, 94°17'41"E	1266–1351	2–42	N			24°18'53"N, 94°16'01"E	404–421	4–27	N		
20	24°52'27"N, 94°18'22"E	1424–1491	15–34	W			24°21'53"N, 94°10'42"E	1142–1247	14–25	NE		
20	24°58'36"N, 94°20'08"E	1225–1330	10–44	W,NW			24°18'50"N, 94°14'50"E	678–726	10–30	NW		
20	24°39'36"N, 94°16'37"E	1242–1268	3–42	N			24°19'58"N, 94°16'29"E	504–552	17–32	SE		

### Vegetation sampling

The vegetation sampling was conducted during the dry months of January and February for fallow aged 5 (JF_5_), 10 (JF_10_), 15 (JF_15_) and 20 (JF_20_) years. We identified a total of 12 sites (four fallow aged plots x three replicates) in each district. At each site, five quadrates of 10 m x 10 m were randomly laid for identifying tree species at different stages of their growth, namely trees, pole, saplings and seedlings. The diameter at breast height (dbh) was measured at 1.37 m from the ground level for individuals with dbh >3 cm. Mature trees were defined as stems with dbh ≥10 cm and height ˃2 m, poles as individuals with dbh ≥3 to <10 cm and height ˃2m, saplings as individuals with collar diameter <3cm and height ˃30 cm to ≤2 m, and seedlings as individuals with collar diameter <3cm and height upto 30 cm. The herbaria at Mizoram University and other published literatures were consulted for correct tree species identification for those which could not be identified in the field. We referred to The Plant List (www.theplantlist.org) for species nomenclature classification.

### Analysis of plant diversity and community structure

Community structure variables such as density, frequency, abundance, basal area and important value index (IVI) of each species in different fallow sites were calculated according to Mueller-Dombois and Ellenberg [16]. Spatial distribution of tree species was determined following Whitford Index [17]. Plant diversity measures such as Shannon-Wiener diversity index (H'), Simpson’s dominance index (C), and Margalef species richness index (D_mg_) were also determined [18–20].

Rarefaction curves based on the number of individuals encountered during the vegetation analysis was plotted to assess the species accumulation pattern for all tree species. The rate of change in species composition with increasing fallow age (β diversity) was calculated following Whittaker [21]. To analyse the similarity in species composition among the replicates of different fallow age stands, Jaccard’s similarity index was represented in the form of a dendrogram [22].

### Biomass estimation

A non-destructive sampling method was adopted to estimate tree above and below-ground biomass and carbon stock of each tree species. With dbh as an independent variable, tree volume (dbh ≥5 cm) was calculated using local and regional based species specific volume equations given by Forest Survey of India (FSI) [23]. Specific gravity of the species was obtained from Global wood density database [24]. The above-ground biomass (AGB) of each individual tree (in kg) was calculated as:

AGB=Treevolume×Specificgravity

However, for those species for which volume equations were not available, AGB was estimated using generic allometric models for tropical forests [25–29]. With the AGB estimated from local volume equation as the base model, the best predictive model was selected on the basis of adjusted R^2^, RMSE, MAD and systematic errors (bias). The AGB for those individuals with dbh<5 cm was derived by using the equation developed by Ali et al. [30]:

ln(AGB)=−3.23+2.17×ln(dbh)

The above ground carbon stock for woody trees with dbh <10 cm, and ≥10 cm were calculated as 46% and 49% of the ABG respectively [31]. The below ground biomass (BGB) was determined by the equation given by Mokany et al. [32] and its carbon stock was calculated as 50% of the BGB.

$$BGB=0.205\times AGB\;when\;AGB<125\;Mg\;ha^{-1},$$

$$BGB=0.235\times AGB\;when\;AGB>125\;Mg\;ha^{-1}$$

Hence, the total living woody biomass carbon was calculated as:

TLWBC=AGBC+BGBC

The uncertainty of total living woody biomass in each fallow stand was estimated as per Higuchi et al. [33]. And the recovery of biomass carbon of the fallow age was computed following the formula given by Mukul et al. [34]:

$$R=(\frac{X_{fallow}}{X_{s}})\times 100$$

Where, X_fallow_ is the fallow site’s biomass carbon and X_s_ is the mean of biomass carbon in the old growth forest. The findings of Waikhom et al. [35] on sacred grove of Manipur was used as reference data for carbon stock recovery estimation. They estimated the biomass using regression equation developed by Chambers et al. [36] which had 38% moisture content. Therefore, biomass estimated from the sacred grove was converted to dry biomass by removing the water content and then the carbon stock was determined. This was done to assess the recovery percent of our estimated dry biomass carbon.

### Statistical analysis

All statistical analysis was conducted using SPSS version 20.0 and PAST version 3.25. One-way ANOVA (Analysis of Variance) was used to examine if there is a significant difference in stem density, basal area, diversity, evenness, dominance and species richness (dependent variables) among different fallow ages (fixed variable). Multiple regression was performed to investigate the relationship of slope, aspect and altitude with species composition along an increasing fallow gradient. The constrained ordination technique of Canonical Correspondence Analysis (CCA) was used to assess the environmental variables contributing to the variation in species composition among fallows. The environmental variables used in CCA were (i) elevation (ii) fallow age (iii) slope, and (iv) aspect. Each of these environmental variables and its association with the variation in the distribution of species were tested using 999 unrestricted permutations.

Prior to analysis of carbon stock, the normality and heteroscedasticity of carbon distribution with increasing fallow age was checked using the Shapiro-Wilk and Levene’s test respectively. To assess variation in carbon stock, we constructed a linear mixed-effect model (LMEM) with fallow age, slope, aspect and elevation as fixed effects, and incorporated sites nested within fallow ages as random intercepts in the model. We applied the information theoretical approach based on the Akaike Information Criterion corrected for small sample sizes (AICc) for model selection, and the model with the lowest AICc score was selected as the best-fit model.

## Results

### Stand characteristic and tree diversity

**Fig 2:**
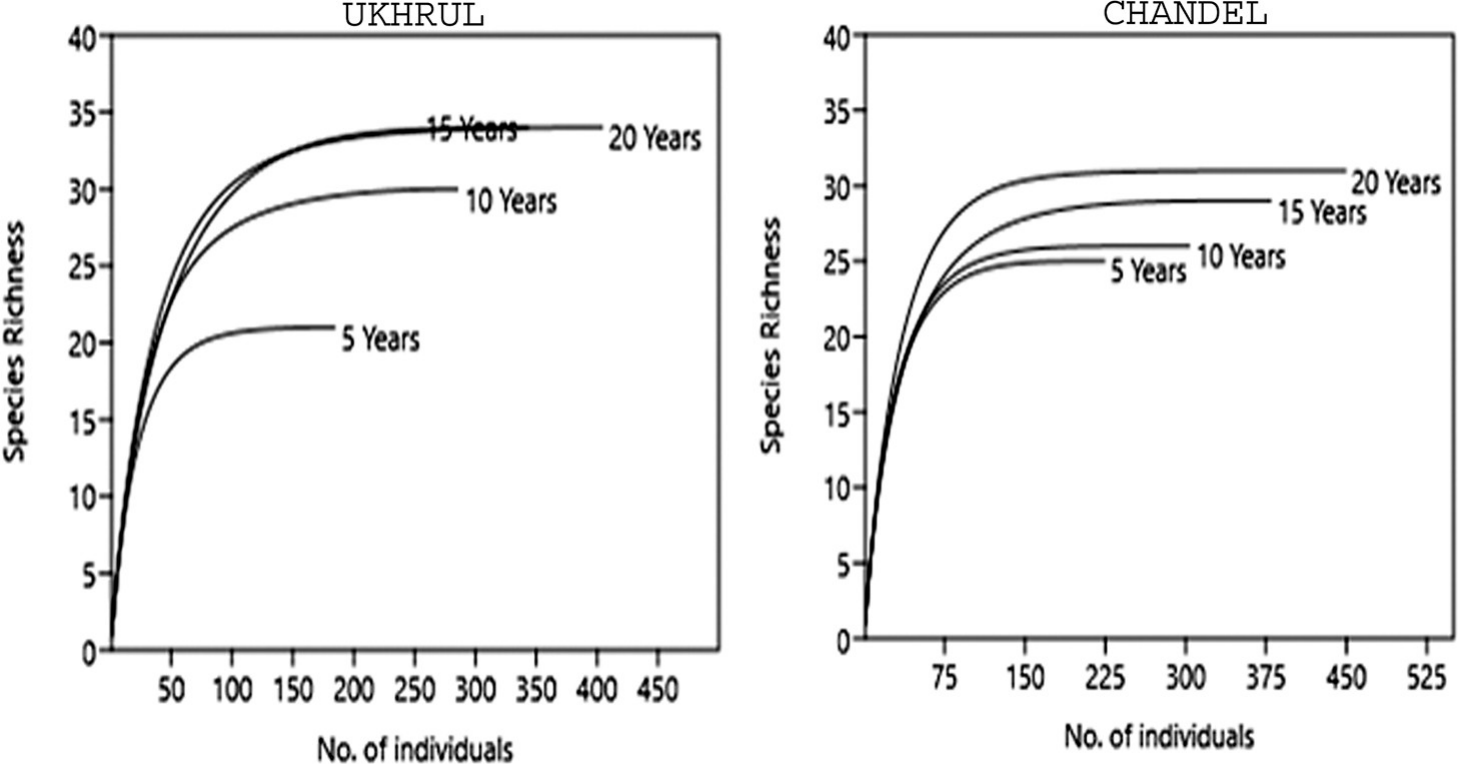
Rarefaction curves of species accumulation in fallows following shifting cultivation.

**Fig 3:**
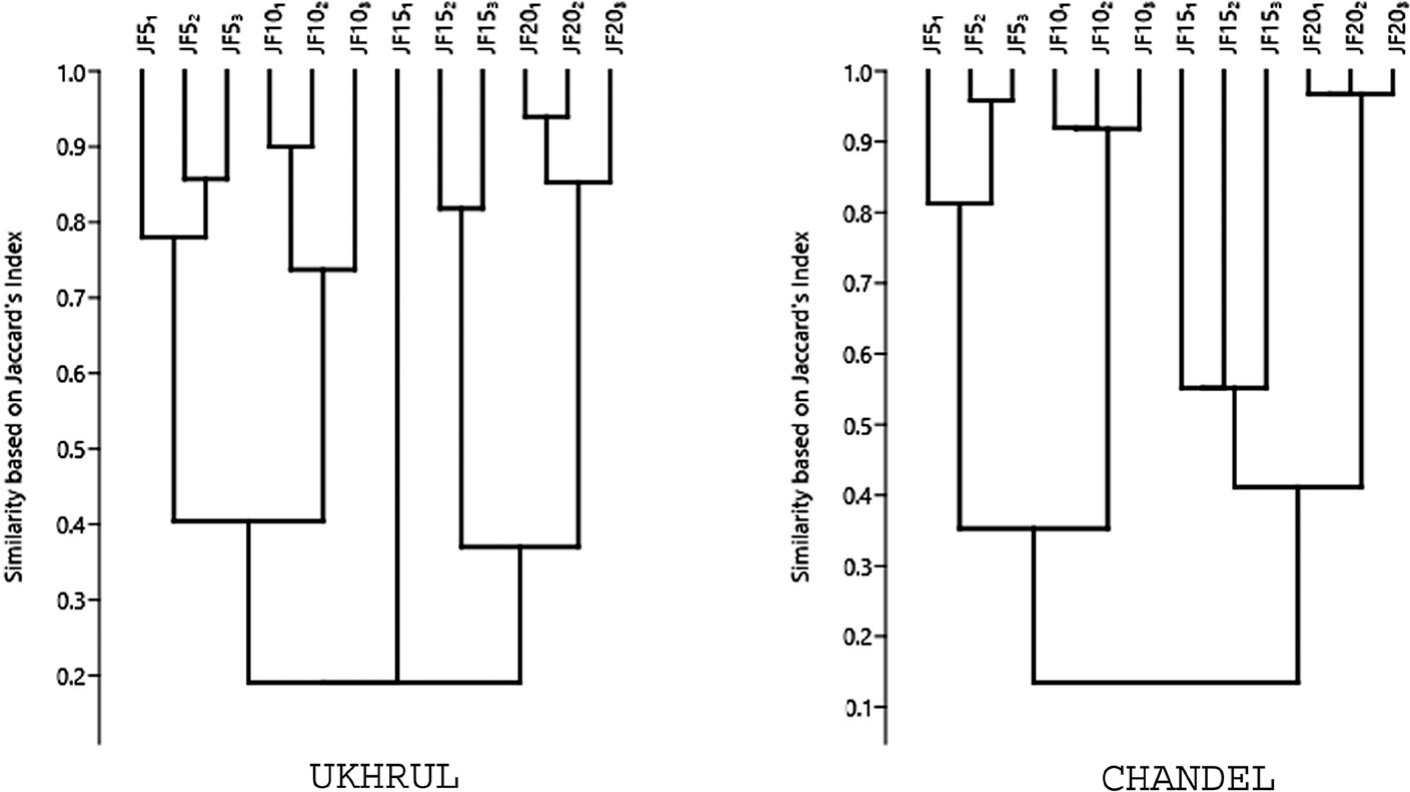
Dendrogram based on similarities in composition of trees in different replicates of Jhum fallows. The values ranges from 0 (no similarity) to 1 (complete similarity).

A total of 58 tree species belonging to 27 families in Ukhrul and 59 tree species belonging to 31 families in Chandel were recorded from the fallow stands. The most dominant family in the study areas was Fagaceae except for the 15 years old fallow site specific to Chandel where the dominant family was Pinaceae. Phytosociological analysis showed high tree diversity and species richness while the low values of dominance index revealed inequitable distribution of trees across increasing fallow age (Table 2). The rarefaction curves of species accumulation indicated that the tree species richness curves in all the fallow ages reached a plateau with increasing fallow age which validates that time duration limits the appearance of new species in fallow ages. The highest number of individuals was recorded from the 20 years fallow with 404 individuals in Ukhrul and 449 in Chandel (Fig 2). Whittaker’s β index showed high level of species similarity between 5 years and 10 years fallow in Ukhrul as well as between 15 years and 20 years fallow in Chandel (Table 3). The three replicates of 5 years fallow, 10 years fallow, 15 years fallow and 20 years fallow were dissimilar to each other with regard to species composition in Ukhrul, while it was contrary in Chandel (Fig 3). Replicates of 15 years fallow showed lowest similarity while the highest was observed in the replicates of 20 years fallow. In Ukhrul, the younger fallows had more similar species which lessened as the fallow age increased. However, Chandel had low species similarity in the younger fallows which increased with the increase in fallow age. Environmental factors namely slope and aspect were found to be similar between 5 and 10 years fallow in Ukhrul as well as between 15 and 20 years fallow in Chandel. This explains for the high species similarity between the fallows (see S1 Table).

**Table 2:** Phytosociological and diversity attributes of tree species in different Jhum fallow stands in the study area.

**Parameters**	**UKHRUL**			
	**JF₅**	**JF₁₀**	**JF₁₅**	**JF₂₀**
No. of species	21	30	34	34
No. of families	15	18	20	21
Density (individuals/ha)	1233.33±42.16	1900±75.59	2286.67±88.3	2693.33±73.33
Basal area (m^2^ha^-1^)	0.85±0.18	9.1±0.48	29.26±1.59	82.1±2.75
Shannon Diversity Index (H´)	1.72±0.07	1.97±0.11	1.84±0.14	2.28±0.09
Simpson Dominance Index (C)	0.21±0.02	0.18±0.03	0.23±0.04	0.13±0.01
Margalef Species Richness Index (D_mg_)	2.24±0.16	2.81±0.3	2.52±0.31	3.48±0.29
**Parameters**	**CHANDEL**			
	**JF₅**	**JF₁₀**	**JF₁₅**	**JF₂₀**
No. of species	25	26	29	31
No. of families	20	16	22	20
Density (individuals/ha)	1493.33±39.6	2020±31.17	2526.67±65.8	2993.33±59.73
Basal area (m^2^ha^-1^)	1.22±0.12	7.97±0.33	32.1±1.19	82.43±3.52
Shannon Diversity Index (H´)	1.61±0.11	1.83±0.08	1.88±0.15	2.44±0.11
Simpson Dominance Index (C)	0.25±0.03	0.19±0.02	0.22±0.04	0.11±0.01
Margalef Species Richness Index (D_mg_)	1.96±0.24	2.13±0.19	2.43±0.28	3.7±0.46

**Table 3:** Whittaker’s β index of the fallow stands in the study area.

	UKHRUL				CHANDEL			
	JF₅	JF₁₀	JF₁₅	JF₂₀	JF₅	JF₁₀	JF₁₅	JF₂₀
**JF₅**	0	0.33	0.60	0.82	0	0.41	0.74	0.71
**JF₁₀**		0	0.41	0.63		0	0.71	0.79
**JF₁₅**			0	0.44			0	0.33
**JF₂₀**				0				0

### Population density and distribution pattern

**Fig 4:**
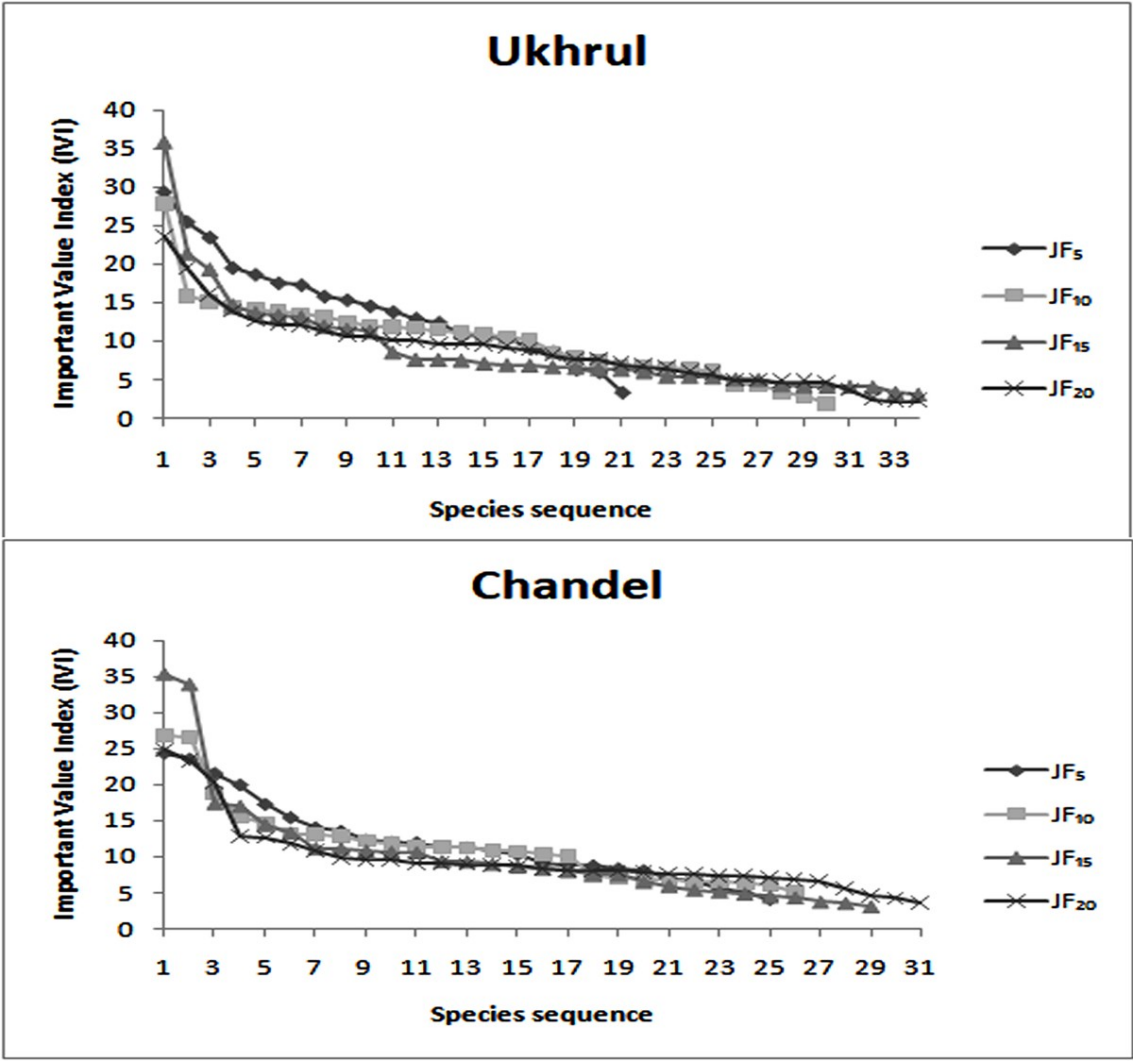
Dominance-diversity curves for tree species at different fallow age.

**Fig 5:**
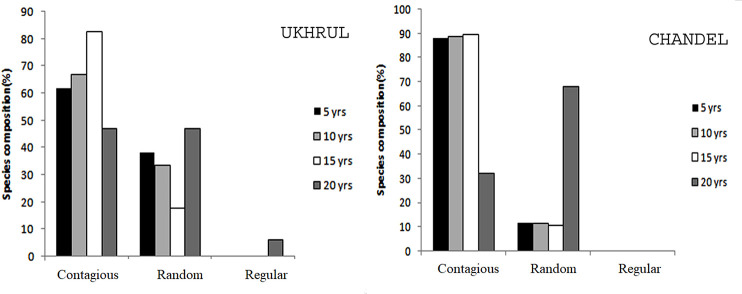
Spatial distribution of tree species at different fallow age.

The distribution analysis of all tree species encountered in each fallow stand is represented in S2 Table. The dominance-distribution curves for all the fallow stands showed normal distribution with high dominance and low equitability in younger fallows which proceed to high equitability as the fallow age increases (Fig 4). In Ukhrul, *Lithocarpus dealbatus* (Hook.f.& Thomson ex Miq.) Rehder (IV1 29.32), *Rhus chinenesis* Mill. (IVI 27.85), *Pinus kesiya* Royle ex Gordon (IVI 35.73) and *Schima wallichii* Choisy (IVI 23.59) were the most dominant species along an increasing fallow gradient, while *Quercus serrata* Murray (IVI 24.26, 26.82) dominated in 5 and 10 years fallow in Chandel and *Schima wallichii* Choisy (IVI 35.25) and *Castanopsis hystrix* Hook. F. & Thomson ex A. DC. (IVI 24.76) dominated the 15 and 20 years fallow respectively. Most of the fallows showed contagious distribution pattern (32–90%) followed by random distribution (10–68%). Regular distribution was observed only for 20 years fallow in Chandel (Fig 5).

### Species recovery in Jhum fallows

In Ukhrul, a distinct rise of 42.9% in the count of tree species was observed from 5 years fallow to 10 years fallow. It further increased by 3.3% in 15 years fallow and then remained stable up to 20 years fallow. Likewise in Chandel, the count of tree species increased by 4% from 5 years fallow to 10 years fallow and which was further enhanced by 11.5% in 15 years fallow and 6.9% in 20 years fallow. Hence, the percent recovery of tree species from 5 years fallow to 20 years fallow was 61.9% and 34.78% in Ukhrul and Chandel respectively.

### Influence of topographical parameters on the species composition

ANOVA results showed that species richness increases significantly from the youngest to the oldest fallow in Ukhrul (*F* = 13.17, *P*<0.001) as well as in Chandel (*F* = 13.28, *P*<0.001). It was also observed that in Ukhrul, significant difference (*F* = 16.71, *P*<0.001 and *F* = 8.707, *P*<0.005) was observed in the 15 years fallow replicates of species richness and stem density as well as in the 10 years fallow replicates of stem density (*F* = 4.244, *P*<0.05). On the other hand, Chandel species richness was found to be significant among the replicates of 15 years fallow (*F* = 3.912, *P*<0.05) and none of the replicates were significant for stem density. Multiple regression results revealed that topographical factors such as slope, aspect and elevation, all had a significant impact on the species richness and stem density along an increasing fallow gradient (Table 4). This finding supports our proposed hypothesis that slope, aspect and elevation have a bearing on the species composition in successional areas.

**Table 4:** Relationship of Jhum fallows with topographical parameters.

		UKHRUL			CHANDEL		
		R^2^	F	P	R^2^	F	P
Species Richness	JF x Slope	0.28	10.86	<0.001**	0.32	13.53	<0.001**
	JF x Aspect	0.26	9.89	<0.001**	0.31	12.98	<0.001**
	JF x Elevation	0.26	9.87	<0.001**	0.31	12.87	<0.001**
Stem Density	JF x Slope	0.79	105.64	<0.001**	0.89	242.91	<0.001**
	JF x Aspect	0.79	110.21	<0.001**	0.90	244.93	<0.001**
	JF x Elevation	0.79	104.35	<0.001**	0.90	248.81	<0.001**

**Significant at P<0.001.

### Species affected by environmental variables

**Fig 6:**
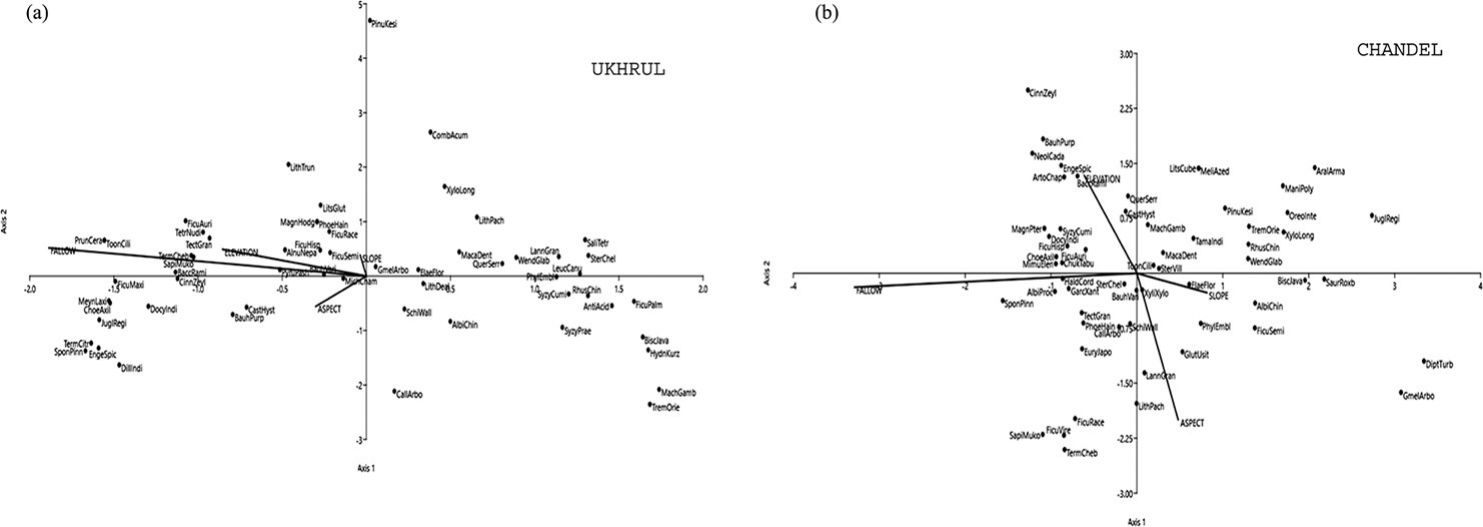
CCA ordination diagram showing distribution and abundance of tree species in the study area. (a) Ukhrul where Trace = 0.799 and p = 0.001. The axis 1 accounts for 68.6% and axis 2 accounts for 19.8% of the variability. (b) Chandel where Trace = 0.541 and p = 0.001. The axis 1 accounts for 77.3% and axis 2 accounts for 14.4% of the variability.

The contribution of environmental variables in the distribution and abundance of tree species along an increasing fallow gradient was determined by Canonical Correspondence Analysis (CCA). Fig 6A showed that the abundance of *Toona ciliata* M. Roem and *Prunus ceraseidos* D. Don were closely associated with the elevation of the sites in Ukhrul, while *Pinus kesiya* Royle ex Gordon and *Toona ciliata* M. Roem were associated with the slope. The fallow age as well as the aspect of the sites also influenced the abundance of *Spondias pinnata* (L. f.) Kurz and *Terminalia citrine* Roxb.ex Fleming. In Chandel, the species mostly associated with the slope and aspect of the sites were *Dipterocarpus turbinatus* C. F. Gaertn and *Gmelina arborea* Roxb. (Fig 6B). Species namely *Cinnamomum zeylanicum* Blume, *Bauhinia purpurea* L. and *Spondias pinnata* (L. f.)Kurz, *Sapindus mukorossi* Gaertn. were associated with the elevation and fallow age simultaneously.

### Potential species for restoration

**Fig 7:**
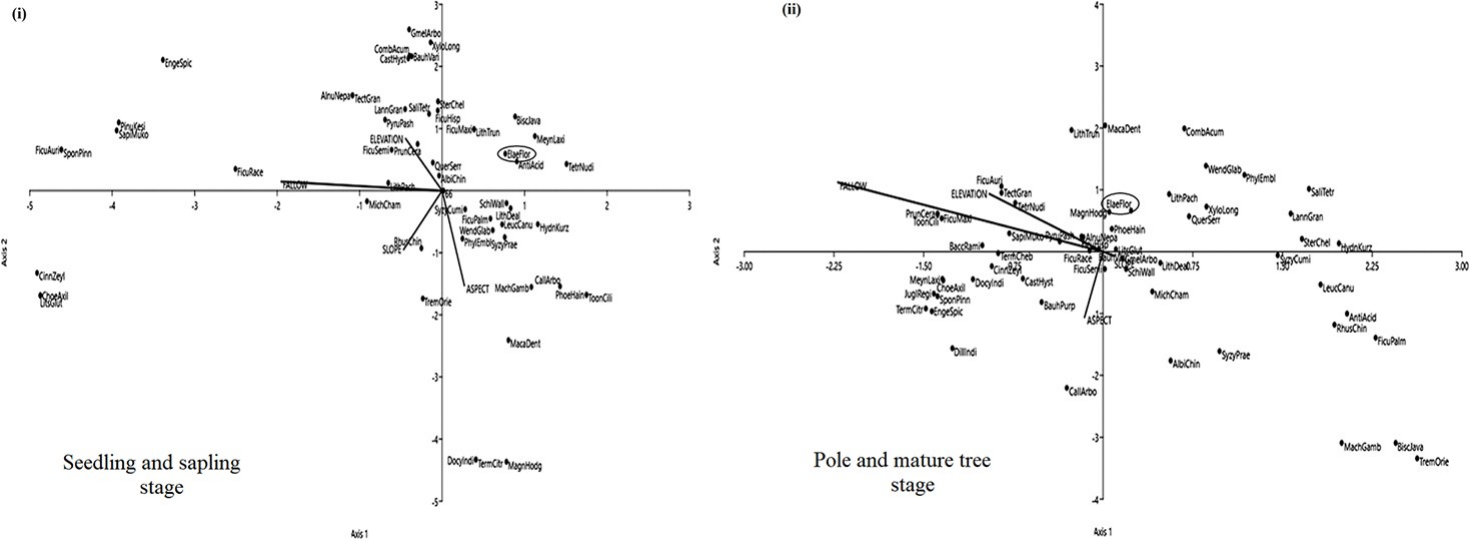
CCA triplot of tree species in Ukhrul. (i) seedling and sapling stage, and (ii) pole and mature stage in Ukhrul from 5 years, 10 years, 15 years and 20 years fallow following shifting cultivation and its relation with selected environmental variables. Full species names are shown in S4 Table.

**Fig 8:**
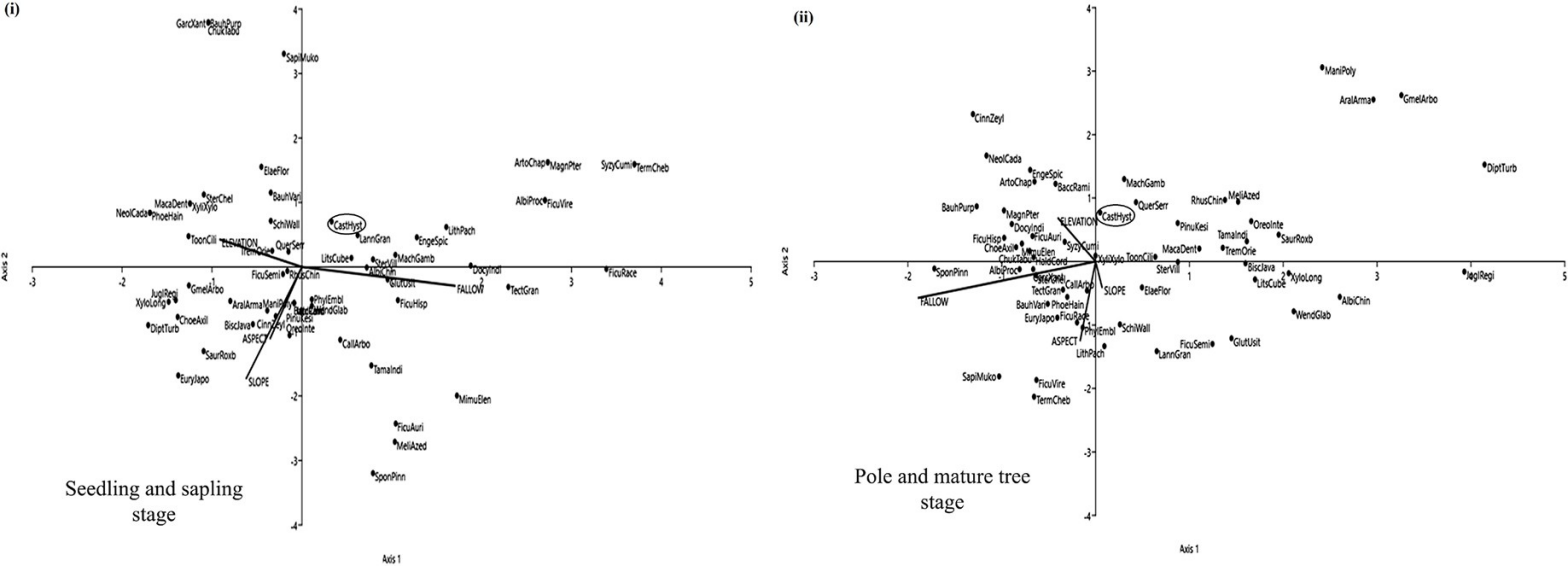
CCA triplot of tree species in Chandel. (i) seedling and sapling stage, and (ii) pole and mature stage in Chandel from 5 years, 10 years, 15 years and 20 years fallow following shifting cultivation and its relation with selected environmental variables. Full species names are shown in S4 Table.

ANOVA results showed that the species composition of tree seedling and sapling decreases significantly from the youngest to the oldest fallow in Ukhrul (*F* = 136.81, *P*<0.001) and Chandel (*F* = 134.84, *P*<0.001) while the pole and mature tree species composition increases from the youngest to the oldest fallow in Ukhrul (*F* = 28.63, *P*<0.001) and Chandel (*F* = 22.70, *P*<0.001) as well. Some species establishes rapidly after abandonment and continues to grow to mature trees in older fallows. One such species is *Elaeocarpus floribundus* Blume in Ukhrul (Fig 7) and *Castanopsis hystrix* Hook. f. & Thomson ex A. DC. in Chandel (Fig 8) (see S3 Table). The CCA triplots further showed that these species (ElaeFlor and CastHyst) were generalist species, that is, they appear opposite to the direction of increasing value of the environmental variables and its relation with the tree species. This testifies that the elevation, fallow age, slope and aspect of the sites have no influence on the abundance of *Elaeocarpus floribundus* Blume and *Castanopsis hystrix* Hook. f. & Thomson ex A. DC. in Ukhrul and Chandel respectively.

### Tree above-ground biomass and total carbon stock

**Fig 9:**
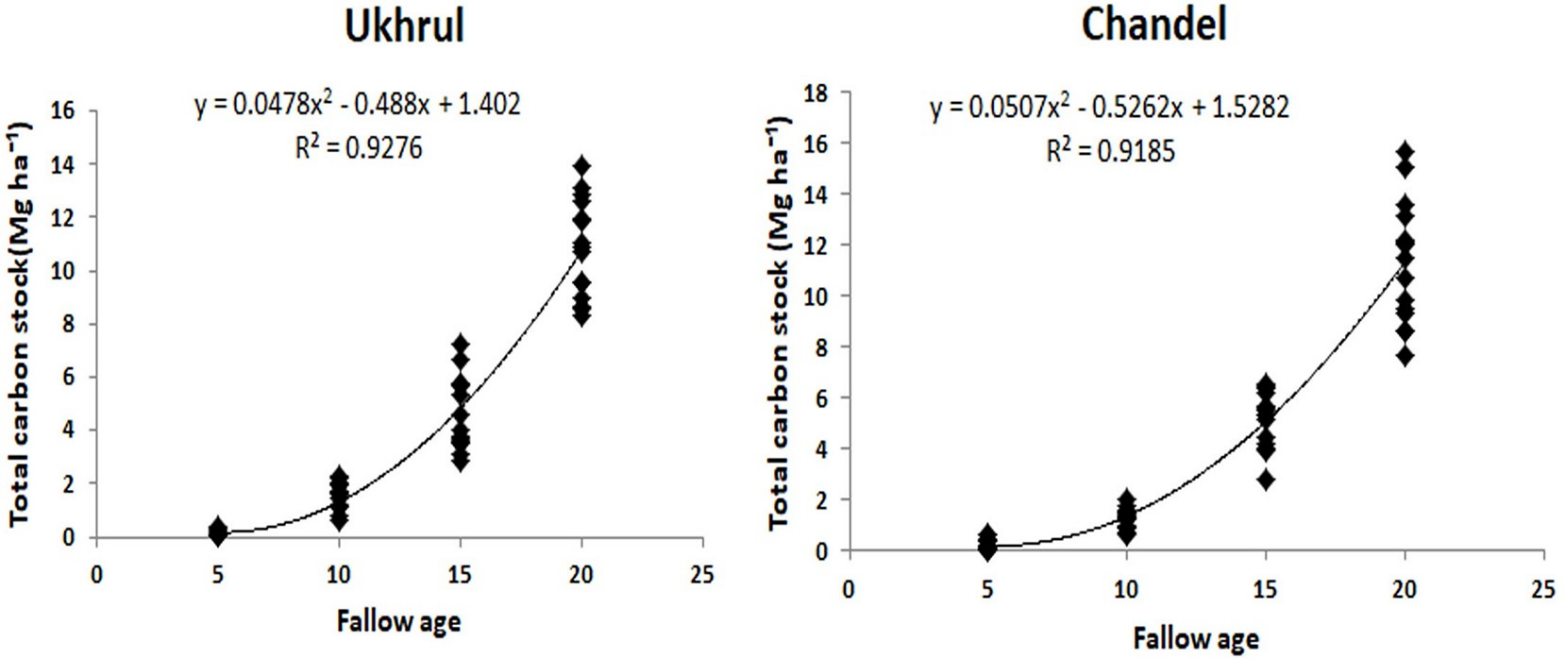
Total living woody biomass carbon estimates for Jhum fallow.

Seven common models for tropical forests were fitted to our data to test the performance of the models with respect to FSI equation [23] (Table 5). The adjusted R^2^ of all the regression models were identical while the lowest values of RMSE and MAD in model 7 verified it as the best model to predict AGB in our study. From the 79 tree species, the AGB of 37 species were evaluated using species specific allometric model given by FSI. For the remaining species, AGB was assessed by following the equation suggested by Nath et al. [29] (see S4 Table). Total living woody biomass carbon (AGBC+BGBC) ranged from 0.98 Mg ha^-1^ to 138.84 Mg ha^-1^ in Ukhrul and 2.5 Mg ha^-1^ to 142.58 Mg ha^-1^ in Chandel. The uncertainty percentage of TLWBC decreased from 78% to 9% in Ukhrul and 56% to 11% in Chandel with increasing fallow age (see S5 Table). The total carbon stock increased rapidly with increased time since abandonment (Fig 9). Pearson correlation analysis showed positive significant correlation for slope and aspect with TLWBC in Ukhrul (*R* = 0.78, *P*<0.001) while elevation was found to be significant for TLWBC in Chandel (*R* = 0.99, *P*<0.001).

**Table 5:** Regression models used for AGB estimation.

Model	Base model	Comparison model	Regression equation	Adjusted R^2^	RMSE	MAD	bias
1	FSI (1996)	Brown et al. (1989)	AGB = 38.4908–11.7883 D+ 1.1926 D^2^	0.72	148.04	106.53	-2.33
2	FSI (1996)	Brown et al. (1989)	AGB = 13.2579–4.8945 D + 0.6713 D^2^	0.72	75.21	54.70	-1.38
3	FSI (1996)	Brown and Iverson (1992)	AGB = 21.297–6.953 D + 0.740 D^2^	0.72	72.32	50.09	-1.11
4	FSI (1996)	Brown (1997)	AGB = 42.69–12.8 (D) + 1.242 (D)^2^	0.72	151.32	108.09	-2.32
5	FSI (1996)	Brown (1997)	AGB = exp (-2.134 + 2.530* ln (DBH))	0.72	113.33	82.94	-2.05
6	FSI (1996)	IPCC (2003)	AGB = exp (-2.289 + 2.649*lnD—0.021*lnD^2^)	0.72	128.38	92.85	-2.18
7	FSI (1996)	Nath et al. (2019)	AGB = 0.18D^2.16^ *1.32	0.72	53.66	40.67	-1.41

### Species contribution to carbon stock

The highest total living woody biomass carbon (TLWBC) in Ukhrul was contributed by *Antidesma acidum* Retz. in 5 years old fallow followed by *Rhus chinensis* Mill., *Pinus kesiya* Royle ex Gordon and *Schima wallichii* Choisy in 10, 15 and 20 years old fallow respectively. In Chandel, *Toona ciliata* M. Roem and *Pinus kesiya* Royle ex Gordon contributed the highest TLWBC in 5 and 15 years old fallow while *Quercus serrata* Murray contributed the highest TLWBC in 10 and 20 years old fallow (Table 6).

**Table 6:** Summary of biomass carbon contributed by individual species in each fallow age.

Tree species	Living Woody Biomass Carbon (Mg ha^-1^)							
	Ukhrul				Chandel			
	JF₅	JF₁₀	JF₁₅	JF₂₀	JF₅	JF₁₀	JF₁₅	JF₂₀
*Albizia chinensis* (Osbeck) Merr.	0.017	0.84		0.97	0.022	0.15		
*Albizia procera* (Roxb.) Benth							0.88	1.69
*Alnus nepalensis* D. Don		1.62	6.70	13.78				
*Antidesma acidum* Retz.	0.318	0.72						
*Aralia armata* (Wall. ex G. Don) Seem.					0.267			
*Artocarpus chaplasha* Roxb.							6.17	4.90
*Baccaurea ramiflora* Lour.			2.06	10.15			3.13	5.38
*Bauhinia purpurea* L.			1.51	1.73				4.28
*Bauhinia variegata* L.		0.78	3.59	5.61	0.011		3.09	13.06
*Bischofia javanica* Blume	0.211	0.64			0.003	2.07		
*Callicarpa arborea* Roxb.	0.056			7.33		0.47	1.21	6.03
*Castanopsis hystrix* Hook. f. & Thomson ex A. DC.		1.33	2.34	15.86	0.036	2.70		21.62
*Choerospondias axillaris* (Roxb.) B. L. Burtt & A. W. Hill				9.48			2.67	8.61
*Chukrasia tabularis* A. Juss.							2.32	7.30
*Cinnamomum zeylanicum* Blume			1.05	3.99				5.91
*Combretum acuminatum* Roxb.		2.10	8.40					
*Dillenia indica* L.				6.38				
*Dipterocarpus turbinatus* C. F. Gaertn					0.261			
*Docynia indica* (Wall.) Decne.			2.16	3.71			1.47	9.18
*Elaeocarpus floribundus* Blume	0.084	1.07	3.30	7.38	0.170		4.36	
*Engelhardtia spicata* Lectan ex Blume				6.89			3.16	8.42
*Eurya japonica* Thunb.							4.35	
*Ficus auriculata* Lour.			1.19	4.51		0.20	0.96	5.82
*Ficus hispida* L. f.		0.13	0.77	2.63		0.05		4.80
*Ficus maxima* Mill.				1.17				
*Ficus palmata* Forssk.	0.013	0.04						
*Ficus racemosa* L.		0.10	0.44	1.73			1.45	
*Ficus semicordata* Buch.-Ham. ex Sm.		0.16	0.80	2.39	0.015	0.41	0.48	
*Ficus virens* Aiton							0.59	2.54
*Garcinia xanthochymus* Hook. f. Ex T. Anderson							4.28	
*Gluta usitata (Wall*.*)* Ding Hou						1.00		
*Gmelina arborea* Roxb.		2.23		4.24	0.213			
*Haldina cordifolia* (Roxb.) Ridsdale							2.33	4.58
*Hydnocarpus kurzü* (King) Warb.	0.003	0.07						
*Juglans regia* L.				3.79	0.091			
*Lannea grandis* Engl.		1.60				1.49	7.99	
*Leucosceptrum canum* Sm.	0.023	2.18	1.74					
*Lithocarpus dealbata* (Hook. F. & Thomson ex Miq.) Rehder	0.205	1.89	6.50	7.89				
*Lithocarpus pachyphyllus* (Kurz) Rehder	0.001	0.58	4.18			2.17	5.87	
*Lithocarpus truncatus* (King ex Hook. f.) Rehder			2.76	3.67				
*Litsea cubeba* (Lour.) Pers.						0.60		
*Litsea glutinosa* (Lour.) C. B. Rob.			2.26					
*Macaranga denticulata* (Blume) Müll. Ang.	0.006		0.81		0.040			3.07
*Machilus gamblei* King ex Hook. f.	0.115					1.01		
*Magnolia hodgsonü* (Hook. f. & Thomson) H. Keng			0.84					
*Magnolia pterocarpa* Roxb.							7.58	8.54
*Maniltoa polyandra* (Roxb.) Harms.					0.138	0.64		
*Melia azedarach* L.						0.27		
*Meyna laxiflora* Robyns				8.42				
*Michelia champaca* L.		1.97		10.66				
*Mimusops elengi* L.							4.68	5.19
*Neolamarckia cadamba* (Roxb.) Bosser								3.00
*Oreocnide integrifolia* (Gaudich.) Miq.					0.004	1.25		
*Phoebe hainesiana* Brandis			1.57				1.62	3.19
*Phyllanthus emblica* L.	0.018	1.91	2.81		0.014	0.74	3.82	
*Pinus kesiya* Royle ex Gordon			11.70		0.054	0.33	12.83	
*Prunus ceraseidos* D. Don				6.43				
*Pyrus pashia* Buch.-Ham. Ex D. Don.		0.85	3.27	11.49				
*Quercus serrata* Murray	0.280	2.03	4.96		0.478	4.62		25.67
*Rhus chinensis* Mill.	0.213	4.13			0.117	1.34		
*Salix tetrasperma* Roxb.		0.37						
*Sapindus mukorossi* Gaertn.			2.03	8.20			3.65	5.15
*Saurauia roxburghii* Wall.					0.005	0.87		
*Schima wallichii* Choisy	0.037	0.32	5.05	19.27	0.045	0.41	10.71	21.13
*Spondias pinnata* (L. f.) Kurz				5.01				6.18
*Sterculia villosa* Roxb.						1.57		
*Stereospermum chelonoides* (L. f.) DC.	0.004	0.50			0.011		0.82	7.86
*Syzygium cumini* (L.) Skeels	0.011	0.97	1.02					8.10
*Syzygium praecox* (Roxb.) Rathakr. & N. C. Nair	0.068	0.07	1.49					
*Tamarindus indica* L.						0.91		
*Tectona grandis* L. f.			0.40	3.77			3.23	4.20
*Terminalia chebula* Retz.			2.44	11.41			5.89	8.61
*Terminalia citrine* Roxb. ex Fleming				12.93				
*Tetrameles nudiflora* R. Br.			0.92	2.73				
*Toona ciliata* M. Roem				8.80	2.377			8.73
*Trema orientalis* (L.) Blume	0.017				0.008	0.67		
*Wendlandia glabrata* DC.	0.013	1.54	5.92		0.010	0.83		
*Xylia xylocarpa* (Roxb.) Taub.					0.018			8.07
*Xylosma longifolia* Clos		1.45	2.62		0.016	0.92		
AGB	1.71	34.20	99.61	234.41	4.42	27.69	111.58	240.82
AGB Carbon	0.80	16.17	48.54	114.81	2.05	12.97	54.28	117.89
BGB Carbon	0.18	3.51	10.21	24.03	0.45	2.84	11.44	24.68
**Total Carbon**	**0.98**	**19.68**	**58.75**	**138.84**	**2.50**	**15.81**	**65.72**	**142.58**

### Factors affecting the recovery of carbon stock in fallow regime

The fallow age was found to have contributed the most to the recovery of carbon from living woody biomass in Ukhrul and Chandel as well (Table 7). It was also observed that the aspect of the sites in Ukhrul was important in explaining the increase in carbon stock. Although slope and elevation displayed a positive correlation with the carbon stock in Ukhrul and Chandel respectively, it did not influence the recovery of carbon stock in the Jhum fallows.

**Table 7:** Summary of LMEM across site carbon stock with environmental variable using SPSS.

UKHRUL									
Parameter	Explanatory variables				Log Likelihood	AIC	AICc	Δ AICc	Weight
	Fallow age	Aspect	Slope	Elevation					
log(TLWBC)	x				**-25.44**	**-13.44**	**-11.86**	**0**	**0.84**
	x	x			-42.47	-16.47	-8.55	3.31	0.16
	x	x	x		-44.86	-10.86	3.71	15.57	0.00
	x	x	x	x	-51.12	-10.12	14.20	26.06	0.00
CHANDEL									
Parameter	Explanatory variables				Log Likelihood	AIC	AICc	Δ AICc	Weight
	Fallow age	Aspect	Slope	Elevation					
log(TLWBC)	x				**27.18**	**39.18**	**40.77**	**0**	**1.00**
	x	x			16.67	42.67	50.58	9.81	0.00
	x	x	x		10.55	44.55	59.12	18.35	0.00
	x	x	x	x	-3.99	38.01	62.32	21.56	0.00

### Recovery of carbon stock

The recovery percent of 20 years fallow in Ukhrul and Chandel was 39% and 40% of the control. Furthermore, based on the regional regression model between AGBC and fallow period, the estimated recovery period was found to be 41 years for Ukhrul and 39 years for Chandel (Table 8).

**Table 8:** Estimated recovery time and recovery percent for above ground biomass carbon (Mg ha^-1^) of 20 years fallow (JF_20_) against control.

Site	AGBC @JF_20_	AGBC (Control)	Model	R^2^	Estimated recovery time (in years)	Recovery %
Ukhrul	114.81	292.54	y = 0.1135x + 7.3853	0.8494	40.59	39.25
Chandel	117.89	292.54	y = 0.1065x + 7.4752	0.8382	38.63	40.30

## Discussion

### Characteristics of secondary successive communities on Jhum fallows

The structure of secondary communities after the abandonment of Jhum fields generally becomes more complex as a result of species recovery. The findings of this study are in agreement with earlier studies which state that there is an increase in the number of trees with increase in fallow age [37]. The basal area is also considered as an essential predictor of ecological succession, and as reported from similar aged fallows in tropical moist forest, the present study also showed a positive association between basal area and fallow age [38, 39]. The values of the Shannon-Wiener diversity index (H') and the Margalef species richness index (D_mg_) along an increasing fallow gradient indicates high diversity and species richness in the successive communities.

### Species composition along an increasing fallow gradient

All species response individually to the process of succession, but the similarity of species among fallows is reported by many researchers. The species similarity between 5 year and 10 year fallow in Ukhrul, and 15 year and 20 year fallow in Chandel suggests that the species compositions of fallows having similar age are more identical than those with more differing ages [40, 41]. The fallows were predominantly composed of fast-growing pioneer and shade-tolerant tree species. Both pioneer and forest tree species got established in the young fallows and dominated at different stages of succession depending on their life span and growth rate [42].

### Relationship between species composition and topographical parameters

In a hilly region, elevation, slope and aspect are considered as key topographical factors that strongly affect the species composition, structure and distribution patterns of vegetation [43]. Elevation affects the micro-climate in several ways and thus results in the variation of communities [44, 45]. In the present study, Chandel situated in lower elevation displays higher species richness and stem density than the Ukhrul site which concurs with the results of other workers [46, 47]. In the northern hemisphere, southern slopes are directly exposed to radiation while northern exposures are cold and humid which could sustain more tree species and stem density [48, 49]. With majority of the sampling sites facing north, our result showed significant relation between species richness and number of individuals along an increasing fallow gradient, identical to the findings of other studies [50, 51].

### Recovery of Jhum fallows

The percent recovery of tree species from 5 years fallow to 20 years fallow was more in Ukhrul than in Chandel because the forest recovery increased with increasing elevation [52]. High altitude species have great potential to adapt to diverse micro-climatic situation as high elevation forests are generally open forests and therefore have more ability to recruit in the deforested areas [53]. In our study, five species in Ukhrul and eight species in Chandel were associated with the youngest fallow in the stage of seedlings and saplings, and survived as mature trees in the oldest fallow. Amongst all tree species, *Elaeocarpus floribundus* Blume and *Castanopsis hystrix* Hook. f. & Thomson ex A. DC. were independent of any environmental variables, and this suggests that they may be capable of effective restoration of degraded forest areas. These tree species can be considered as generalist species as they rapidly establish after abandonment and survive in dense forest. Since both the species occur naturally in wild, they can be utilized for active restoration to accelerate the recovery process of fallows.

Our result showed a positive relationship between the fallow period and the total living woody biomass carbon which is in accord with other works related to recovery following shifting cultivation in the tropical forests and neotropical secondary forests [54–56].

### Sources of uncertainty

Generally uncertainty in carbon stock estimation arises mainly due to inventory protocol and method utilized to convert tree measurement to biomass. The latter can be quantified based on allometric equations or on biomass expansion factor. Conversion from tree volume to carbon content results in larger uncertainties as compared to the use of allometric models [57]. This explains the high uncertainty percentage in the present study.

### Implications for management of Jhum fallows

Over the years, it is accepted that regrowth forests in the tropics provide similar ecosystem goods and services as old-growth forests [58]. This region however has been a huge contributor of greenhouse gas emission due to deforestation and adapting proper land-use system will help recover biomass carbon stock in the forests [59]. Tree plantation may be adapted to accelerate the steady process of natural succession in the fallows thereby restoring the species richness and enhancing the carbon sinks. And as per our findings, *Elaeocarpus floribundus* Blume and *Castanopsis hystrix* Hook. f. & Thomson ex A. DC. which occurs naturally in wild can be utilized as potential species for active restoration to accelerate the recovery process of fallows.

## Conclusion

With the decrease in the pristine forests, secondary forests are of greater importance and value in the conservation and restoration of tropical biodiversity. Species richness and basal area recovered relatively with time since abandonment in our study, while the species composition recovery showed resemblance between similar-aged fallows. Topographical factors namely elevation, slope and aspect influenced the high species richness and stem density of fallows in Chandel than in Ukhrul. Regeneration fallow stands are considered to mitigate forest degradation and contribute to global carbon sequestration. We found that the fallow age plays a vital role in recovering aboveground biomass from living woody species followed by the aspect of the site. In this study, we identified two species which can be used in the active restoration of Jhum fallows and which colonize early fallows and appear naturally in the forests. The seedlings of these tree species can be collected from the surrounding forests or germinated and planted in Jhum fallows to accelerate natural succession. Tropical Amazonia had observed 90% survival in the transplantation of seedlings into fallows with partially established vegetation [60]. This would facilitate quicker rehabilitation and reinstate the soil nutrients making the soil reusable in a short term, such that the expansion of slashing and burning of primary forests for cultivation would be reduced. A gradual increase in the diversity of woody species along the fallow age evinces the potential of longer fallow forests to enhance carbon stock. Further, the results would help in formulating appropriate policy interventions on conservation strategies and developing climate change mitigation policies for efficient preservation of forest carbon stocks.

## Supporting information

S1 Table Tukey HSD multiple comparison between Jhum fallows with respect to environmental factors of Ukhrul and Chandel. (DOCX)

S2 Table Density, basal area (m2 ha-1) and IVI of tree species in Jhum fallows. (DOCX)

S3 Table Abundance of species in seedling and sapling stage and pole and mature stage after abandonment. (DOCX)

S4 Table Allometric models used in the present study with species name in short. (DOCX)

S5 Table Uncertainty percentage for the parameters of TLWBC in all fallow age. (DOCX)
