# Supplementary material for: Pattern of forest recovery and carbon stock following shifting cultivation in Manipur, North-East India
Source: PLoS One. 2020 Oct 8;15(10):e0239906. doi: 10.1371/journal.pone.0239906 (PMC7544089; doi:10.1371/journal.pone.0239906)
Supplement: S1 Table — (DOCX) [file pone.0239906.s001.docx]

**S1 Table Tukey HSD multiple comparison between Jhum fallows with respect to environmental factors of Ukhrul and Chandel**

| Environmental factor | Interaction | | P-value | |
| --- | --- | --- | --- | --- |
|  | Dependent | Independent | Ukhrul | Chandel |
| Elevation | JF_5_ | JF_10_ | <0.001** | 0.235 |
|  |  | JF_15_ | <0.001** | 0.185 |
|  |  | JF_20_ | <0.001** | 0.208 |
|  | JF_10_ | JF_5_ | <0.001** | 0.235 |
|  |  | JF_15_ | 0.814 | 0.998 |
|  |  | JF_20_ | 1 | 0.002* |
|  | JF_15_ | JF_5_ | <0.001** | 0.185 |
|  |  | JF_10_ | 0.814 | 0.998 |
|  |  | JF_20_ | 0.756 | 0.001* |
|  | JF_20_ | JF_5_ | <0.001** | 0.208 |
|  |  | JF_10_ | 1 | 0.002* |
|  |  | JF_15_ | 0.756 | 0.001* |
| Slope | JF_5_ | JF_10_ | 0.989 | 0.955 |
|  |  | JF_15_ | 1 | 0.952 |
|  |  | JF_20_ | 0.998 | 1 |
|  | JF_10_ | JF_5_ | 0.989 | 0.955 |
|  |  | JF_15_ | 0.978 | 1 |
|  |  | JF_20_ | 0.965 | 0.929 |
|  | JF_15_ | JF_5_ | 1 | 0.952 |
|  |  | JF_10_ | 0.978 | 1 |
|  |  | JF_20_ | 1 | 0.925 |
|  | JF_20_ | JF_5_ | 0.998 | 1 |
|  |  | JF_10_ | 0.965 | 0.929 |
|  |  | JF_15_ | 1 | 0.925 |
| Aspect | JF_5_ | JF_10_ | 0.104 | 0.997 |
|  |  | JF_15_ | 0.056 | 0.936 |
|  |  | JF_20_ | 0.816 | 1 |
|  | JF_10_ | JF_5_ | 0.104 | 0.997 |
|  |  | JF_15_ | 0.989 | 0.863 |
|  |  | JF_20_ | 0.013* | 0.991 |
|  | JF_15_ | JF_5_ | 0.056 | 0.936 |
|  |  | JF_10_ | 0.989 | 0.863 |
|  |  | JF_20_ | 0.006* | 0.96 |
|  | JF_20_ | JF_5_ | 0.816 | 1 |
|  |  | JF_10_ | 0.013* | 0.991 |
|  |  | JF_15_ | 0.006* | 0.96 |

JF5-5 years Jhum fallow, JF10-10 years Jhum fallow, JF15-15 years Jhum fallow, JF20-20 years Jhum fallow, **Significant at P<0.001, *Significant at P<0.05
