# Supplementary material for: Pattern of forest recovery and carbon stock following shifting cultivation in Manipur, North-East India
Source: PLoS One. 2020 Oct 8;15(10):e0239906. doi: 10.1371/journal.pone.0239906 (PMC7544089; doi:10.1371/journal.pone.0239906)
Supplement: S2 Table — (DOCX) [file pone.0239906.s002.docx]

**S2 Table Density, basal area (m^2^ ha^-1^) and IVI of tree species in Jhum fallows**

| **UKHRUL** | | | | | | | | | | | | |
| --- | --- | --- | --- | --- | --- | --- | --- | --- | --- | --- | --- | --- |
| Tree species | 5 years | | | 10 years | | | 15 years | | | 20 years | | |
|  | D | BA | IVI | D | BA | IVI | D | BA | IVI | D | BA | IVI |
| *Albizia chinensis* (Osbeck) Merr. | 0.4 | 0.03 | 10.67 | 0.47 | 0.21 | 8.43 |  |  |  | 0.40 | 0.80 | 4.59 |
| *Alnus nepalensis* D. Don |  |  |  | 0.53 | 0.37 | 9.95 | 1.00 | 1.45 | 13.03 | 1.80 | 5.06 | 16.07 |
| *Antidesma acidum* Retz. | 0.4 | 0.09 | 17.25 | 0.33 | 0.17 | 7.37 |  |  |  |  |  |  |
| *Baccaurea ramiflora* Lour. |  |  |  |  |  |  | 0.40 | 0.45 | 7.00 | 0.87 | 2.19 | 10.70 |
| *Bauhinia purpurea* L. |  |  |  |  |  |  | 0.40 | 0.54 | 6.56 | 0.13 | 0.53 | 2.22 |
| *Bauhinia variegata* L. |  |  |  | 0.73 | 0.43 | 12.46 | 0.87 | 1.29 | 11.14 | 0.40 | 1.50 | 6.00 |
| *Bischofia javanica* Blume | 0.67 | 0.07 | 19.48 | 0.33 | 0.12 | 6.01 |  |  |  |  |  |  |
| *Callicarpa arborea* Roxb. | 0.53 | 0.05 | 15.31 |  |  |  |  |  |  | 1.13 | 3.67 | 11.37 |
| *Castanopsis hystrix* Hook. f. & Thomson ex A. DC. |  |  |  | 0.93 | 0.42 | 11.89 | 0.60 | 0.87 | 6.34 | 2.07 | 6.96 | 19.37 |
| *Choerospondias axillaris* (Roxb.) B. L. Burtt & A. W. Hill |  |  |  |  |  |  |  |  |  | 0.87 | 2.21 | 9.66 |
| *Cinnamomum zeylanicum* Blume |  |  |  |  |  |  | 0.40 | 0.43 | 6.18 | 0.60 | 1.84 | 7.68 |
| *Combretum acuminatum* Roxb. |  |  |  | 0.87 | 0.49 | 13.83 | 1.60 | 1.85 | 19.23 |  |  |  |
| *Dillenia indica* L. |  |  |  |  |  |  |  |  |  | 0.47 | 1.70 | 7.01 |
| *Docynia indica* (Wall.) Decne. |  |  |  |  |  |  | 0.27 | 0.46 | 4.23 | 0.73 | 2.12 | 9.58 |
| *Elaeocarpus* *floribundus* Blume | 0.67 | 0.04 | 15.77 | 0.60 | 0.40 | 11.37 | 1.07 | 1.31 | 13.58 | 0.87 | 2.75 | 9.25 |
| *Engelhardtia* *spicata* Lectan ex Blume |  |  |  |  |  |  |  |  |  | 0.80 | 1.96 | 10.18 |
| *Ficus* *auriculata* Lour. |  |  |  |  |  |  | 0.40 | 0.51 | 4.97 | 0.40 | 1.71 | 5.71 |
| *Ficus* *hispida* L. f. |  |  |  | 0.27 | 0.13 | 4.34 | 0.27 | 0.44 | 4.88 | 0.47 | 1.29 | 4.91 |
| *Ficus* *maxima* Mill. |  |  |  |  |  |  |  |  |  | 0.20 | 0.63 | 2.58 |
| *Ficus* *palmata* Forssk. | 0.27 | 0.02 | 8.59 | 0.13 | 0.05 | 2.69 |  |  |  |  |  |  |
| *Ficus* *racemosa* L. |  |  |  | 0.13 | 0.09 | 3.13 | 0.40 | 0.36 | 5.19 | 0.40 | 0.90 | 3.66 |
| *Ficus* *semicordata* Buch.-Ham. ex Sm. |  |  |  | 0.53 | 0.08 | 6.65 | 0.27 | 0.40 | 4.02 | 0.33 | 1.07 | 4.69 |
| *Gmelina* *arborea* Roxb. |  |  |  | 0.93 | 0.10 | 10.43 |  |  |  | 0.40 | 1.05 | 4.91 |
| *Hydnocarpus* *kurzü* (King) Warb. | 0.40 | 0.01 | 8.99 | 0.20 | 0.08 | 4.12 |  |  |  |  |  |  |
| *Juglans* *regia* L. |  |  |  |  |  |  |  |  |  | 0.67 | 2.18 | 8.88 |
| *Lannea* *grandis* Engl. |  |  |  | 0.67 | 0.39 | 10.84 |  |  |  |  |  |  |
| *Leucosceptrum* *canum* Sm. | 1.00 | 0.04 | 17.49 | 0.73 | 0.50 | 13.97 | 0.27 | 0.38 | 3.20 |  |  |  |
| *Lithocarpus* *dealbata* (Hook. F. & Thomson ex Miq.) Rehder | 1.40 | 0.10 | 29.32 | 1.13 | 0.47 | 14.25 | 0.93 | 1.40 | 11.83 | 1.20 | 3.62 | 12.09 |
| *Lithocarpus* *pachyphyllus* (Kurz) Rehder | 0.13 | 0.002 | 3.31 | 0.33 | 0.14 | 6.24 | 0.87 | 0.92 | 11.38 |  |  |  |
| *Lithocarpus* *truncatus* (King ex Hook. f.) Rehder |  |  |  |  |  |  | 0.33 | 0.59 | 6.42 | 0.20 | 0.83 | 2.29 |
| *Litsea* *glutinosa* (Lour.) C. B. Rob. |  |  |  |  |  |  | 0.47 | 0.51 | 7.49 |  |  |  |
| *Macaranga* *denticulata* (Blume) Müll. Ang. | 0.27 | 0.01 | 6.29 |  |  |  | 0.40 | 0.61 | 6.78 |  |  |  |
| *Machilus* *gamblei* King ex Hook. f. | 0.40 | 0.04 | 12.38 |  |  |  |  |  |  |  |  |  |
| *Magnolia* *hodgsonü* (Hook. f. & Thomson) H. Keng |  |  |  |  |  |  | 0.27 | 0.19 | 4.05 |  |  |  |
| *Meyna* *laxiflora* Robyns |  |  |  |  |  |  |  |  |  | 0.60 | 1.78 | 8.14 |
| *Michelia* *champaca* L. |  |  |  | 0.93 | 0.51 | 15.11 |  |  |  | 1.00 | 2.60 | 10.10 |
| *Phoebe* *hainesiana* Brandis |  |  |  |  |  |  | 0.27 | 0.34 | 5.30 |  |  |  |
| *Phyllanthus* *emblica* L. | 0.73 | 0.03 | 14.51 | 1.13 | 0.48 | 15.77 | 0.40 | 0.61 | 5.30 |  |  |  |
| *Pinus* *kesiya* Royle ex Gordon |  |  |  |  |  |  | 3.80 | 4.51 | 35.73 |  |  |  |
| *Prunus* *ceraseidos* D. Don |  |  |  |  |  |  |  |  |  | 0.53 | 1.52 | 6.51 |
| *Pyrus* *pashia* Buch.-Ham. Ex D. Don. |  |  |  | 0.47 | 0.21 | 7.71 | 0.47 | 0.71 | 7.41 | 1.00 | 2.68 | 10.72 |
| *Quercus* *serrata* Murray | 0.60 | 0.03 | 13.75 | 0.93 | 0.30 | 13.41 | 1.27 | 1.34 | 14.57 |  |  |  |
| *Rhus* *chinensis* Mill. | 0.67 | 0.12 | 23.41 | 2.00 | 1.01 | 27.85 |  |  |  |  |  |  |
| *Salix* *tetrasperma* Roxb. |  |  |  | 0.33 | 0.09 | 6.44 |  |  |  |  |  |  |
| *Sapindus* *mukorossi* Gaertn. |  |  |  |  |  |  | 0.33 | 0.44 | 5.92 | 0.53 | 2.00 | 7.63 |
| *Saurauia* *roxburghii* Wall. |  |  |  |  |  |  |  |  |  |  |  |  |
| *Schima* *wallichii* Choisy | 1.53 | 0.06 | 25.43 | 0.87 | 0.31 | 11.76 | 1.87 | 2.53 | 21.26 | 2.67 | 8.15 | 23.59 |
| *Spondias* *pinnata* (L. f.) Kurz |  |  |  |  |  |  |  |  |  | 1.07 | 3.62 | 12.66 |
| *Stereospermum* *chelonoides* (L. f.) DC. | 0.27 | 0.01 | 5.94 | 0.33 | 0.10 | 6.51 |  |  |  |  |  |  |
| *Syzygium* *cumini* (L.) Skeels | 0.47 | 0.03 | 10.72 | 0.73 | 0.36 | 13.08 | 0.13 | 0.27 | 2.99 |  |  |  |
| *Syzygium* *praecox* (Roxb.) Rathakr. & N. C. Nair | 0.53 | 0.08 | 18.60 | 0.13 | 0.03 | 1.73 | 0.27 | 0.37 | 3.92 |  |  |  |
| *Tectona* *grandis* L. f. |  |  |  |  |  |  | 0.27 | 0.20 | 4.08 | 0.47 | 1.19 | 4.79 |
| *Terminalia* *chebula* Retz. |  |  |  |  |  |  | 0.47 | 0.53 | 6.82 | 0.73 | 2.58 | 9.61 |
| *Terminalia* *citrine* Roxb. ex Fleming |  |  |  |  |  |  |  |  |  | 1.00 | 3.03 | 12.23 |
| *Tetrameles* *nudiflora* R. Br. |  |  |  |  |  |  | 0.40 | 0.60 | 7.52 | 0.60 | 1.49 | 6.72 |
| *Toona* *ciliata* M. Roem |  |  |  |  |  |  |  |  |  | 1.33 | 4.71 | 13.91 |
| *Trema* *orientalis* (L.) Blume | 0.40 | 0.03 | 10.33 |  |  |  |  |  |  |  |  |  |
| *Wendlandia* *glabrata* DC. | 0.53 | 0.02 | 12.89 | 0.73 | 0.36 | 11.63 | 1.00 | 1.29 | 13.24 |  |  |  |
| *Xylosma* *longifolia* Clos |  |  |  | 0.53 | 0.34 | 11.00 | 0.47 | 0.57 | 8.45 |  |  |  |
| **CHANDEL** | | | | | | | | | | | | |
| Tree species | 5 years | | | 10 years | | | 15 years | | | 20 years | | |
|  | D | BA | IVI | D | BA | IVI | D | BA | IVI | D | BA | IVI |
| *Albizia* *chinensis* (Osbeck) Merr. | 0.33 | 0.05 | 10.28 | 1.00 | 0.27 | 14.64 |  |  |  |  |  |  |
| *Albizia* *procera* (Roxb.) Benth |  |  |  |  |  |  | 0.47 | 0.42 | 5.40 | 0.67 | 1.91 | 7.48 |
| *Aralia* *armata* (Wall. ex G. Don) Seem. | 0.53 | 0.09 | 15.37 |  |  |  |  |  |  |  |  |  |
| *Artocarpus* *chaplasha* Roxb. |  |  |  |  |  |  | 0.47 | 0.52 | 6.47 | 0.87 | 2.71 | 9.61 |
| *Baccaurea* *ramiflora* Lour. |  |  |  |  |  |  | 0.60 | 0.69 | 8.28 | 0.80 | 1.75 | 7.74 |
| *Bauhinia* *purpurea* L. |  |  |  |  |  |  |  |  |  | 0.53 | 1.15 | 5.63 |
| *Bauhinia* *variegata* L. | 0.60 | 0.02 | 10.75 |  |  |  | 0.93 | 1.18 | 11.12 | 1.33 | 3.57 | 12.71 |
| *Bischofia* *javanica* Blume | 0.20 | 0.01 | 5.01 | 0.73 | 0.37 | 12.78 |  |  |  |  |  |  |
| *Callicarpa* *arborea* Roxb. |  |  |  | 0.80 | 0.30 | 11.29 | 0.47 | 0.59 | 5.93 | 0.93 | 2.60 | 9.71 |
| *Castanopsis* *hystrix* Hook. f. & Thomson ex A. DC. | 1.60 | 0.06 | 19.88 | 2.20 | 0.82 | 26.55 |  |  |  | 2.93 | 8.29 | 24.76 |
| *Choerospondias* *axillaris* (Roxb.) B. L. Burtt & A. W. Hill |  |  |  |  |  |  | 0.60 | 0.60 | 7.99 | 0.73 | 2.19 | 7.56 |
| *Chukrasia* *tabularis* A. Juss. |  |  |  |  |  |  | 0.27 | 0.51 | 4.88 | 0.87 | 2.12 | 8.40 |
| *Cinnamomum* *zeylanicum* Blume |  |  |  |  |  |  |  |  |  | 1.13 | 2.60 | 9.88 |
| *Dipterocarpus* *turbinatus* C. F. Gaertn | 0.47 | 0.09 | 13.51 |  |  |  |  |  |  |  |  |  |
| *Docynia* *indica* (Wall.) Decne. |  |  |  |  |  |  | 0.27 | 0.32 | 3.56 | 0.87 | 2.24 | 9.05 |
| *Elaeocarpus* *floribundus* Blume | 0.80 | 0.08 | 17.20 |  |  |  | 1.47 | 1.79 | 17.39 |  |  |  |
| *Engelhardtia* *spicata* Lectan ex Blume |  |  |  |  |  |  | 0.60 | 0.70 | 7.55 | 0.73 | 2.32 | 8.21 |
| *Eurya* *japonica* Thunb. |  |  |  |  |  |  | 0.67 | 0.94 | 8.58 |  |  |  |
| *Ficus* *auriculata* Lour. |  |  |  | 0.33 | 0.13 | 6.93 | 0.27 | 0.38 | 3.74 | 0.73 | 2.55 | 8.98 |
| *Ficus* *hispida* L. f. |  |  |  | 0.33 | 0.06 | 5.07 |  |  |  | 0.73 | 2.51 | 8.93 |
| *Ficus* *racemosa* L. |  |  |  |  |  |  | 0.60 | 0.91 | 8.96 |  |  |  |
| *Ficus* *semicordata* Buch.-Ham. ex Sm. | 0.27 | 0.02 | 8.04 | 0.60 | 0.33 | 10.71 | 0.20 | 0.26 | 3.10 |  |  |  |
| *Ficus* *virens* Aiton |  |  |  |  |  |  | 0.27 | 0.33 | 4.35 | 0.33 | 0.99 | 4.28 |
| *Garcinia* *xanthochymus* Hook. f. Ex T. Anderson |  |  |  |  |  |  | 0.80 | 0.94 | 10.61 |  |  |  |
| *Gluta* *usitata* (Wall.) Ding Hou |  |  |  | 0.73 | 0.25 | 11.29 |  |  |  |  |  |  |
| *Gmelina* *arborea* Roxb. | 0.53 | 0.05 | 11.90 |  |  |  |  |  |  |  |  |  |
| *Haldina* *cordifolia* (Roxb.) Ridsdale |  |  |  |  |  |  | 0.53 | 0.52 | 7.49 | 0.60 | 0.98 | 6.63 |
| *Juglans* *regia* L. | 0.53 | 0.05 | 12.26 |  |  |  |  |  |  |  |  |  |
| *Lannea* *grandis* Engl. |  |  |  | 1.00 | 0.37 | 13.19 | 1.40 | 1.74 | 16.99 |  |  |  |
| *Lithocarpus* *pachyphyllus* (Kurz) Rehder |  |  |  | 0.93 | 0.52 | 15.60 | 1.20 | 1.29 | 13.29 |  |  |  |
| *Litsea* *cubeba* (Lour.) Pers. |  |  |  | 0.33 | 0.14 | 6.13 |  |  |  |  |  |  |
| *Macaranga* *denticulata* (Blume) Müll. Ang. | 0.47 | 0.09 | 13.98 |  |  |  |  |  |  | 0.67 | 1.80 | 6.86 |
| *Machilus* *gamblei* King ex Hook. f. |  |  |  | 0.80 | 0.27 | 11.80 |  |  |  |  |  |  |
| *Magnolia* *pterocarpa* Roxb. |  |  |  |  |  |  | 1.20 | 1.64 | 14.37 | 0.87 | 2.40 | 8.75 |
| *Maniltoa* *polyandra* (Roxb.) Harms. | 0.33 | 0.04 | 9.00 | 0.40 | 0.15 | 6.62 |  |  |  |  |  |  |
| *Melia* *azedarach* L. |  |  |  | 0.27 | 0.21 | 6.60 |  |  |  |  |  |  |
| *Mimusops* *elengi* L. |  |  |  |  |  |  | 0.80 | 1.02 | 10.85 | 0.73 | 1.77 | 8.03 |
| *Neolamarckia* *cadamba* (Roxb.) Bosser |  |  |  |  |  |  |  |  |  | 1.00 | 3.00 | 10.91 |
| *Oreocnide* *integrifolia* (Gaudich.) Miq. | 0.20 | 0.01 | 4.06 | 0.67 | 0.31 | 10.79 |  |  |  |  |  |  |
| *Phoebe* *hainesiana* Brandis |  |  |  |  |  |  | 0.27 | 0.35 | 5.16 | 0.27 | 0.66 | 3.65 |
| *Phyllanthus* *emblica* L. | 0.40 | 0.02 | 8.79 | 0.80 | 0.20 | 10.08 | 0.93 | 0.85 | 9.35 |  |  |  |
| *Pinus* *kesiya* Royle ex Gordon | 0.47 | 0.05 | 11.38 | 0.47 | 0.18 | 6.35 | 3.73 | 4.90 | 33.80 |  |  |  |
| *Quercus* *serrata* Murray | 1.33 | 0.13 | 24.26 | 2.20 | 0.84 | 26.82 |  |  |  | 2.33 | 5.82 | 20.25 |
| *Rhus* *chinensis* Mill. | 0.60 | 0.05 | 11.99 | 0.87 | 0.34 | 13.07 |  |  |  |  |  |  |
| *Sapindus* *mukorossi* Gaertn. |  |  |  |  |  |  | 0.67 | 0.93 | 9.28 | 0.67 | 1.83 | 7.39 |
| *Saurauia* *roxburghii* Wall. | 0.27 | 0.01 | 5.60 | 0.40 | 0.21 | 7.35 |  |  |  |  |  |  |
| *Schima* *wallichii* Choisy | 1.40 | 0.08 | 21.49 | 1.60 | 0.51 | 18.84 | 3.67 | 4.97 | 35.25 | 2.67 | 7.83 | 23.31 |
| *Spondias* *pinnata* (L. f.) Kurz |  |  |  |  |  |  |  |  |  | 1.20 | 3.54 | 11.73 |
| *Sterculia* *villosa* Roxb. |  |  |  | 0.60 | 0.37 | 11.24 |  |  |  |  |  |  |
| *Stereospermum* *chelonoides* (L. f.) DC. | 0.33 | 0.02 | 6.95 |  |  |  | 0.27 | 0.38 | 4.50 | 0.73 | 2.16 | 8.01 |
| *Syzygium* *cumini* (L.) Skeels |  |  |  |  |  |  |  |  |  | 0.87 | 2.07 | 7.86 |
| *Tamarindus* *indica* L. |  |  |  | 0.60 | 0.23 | 10.31 |  |  |  |  |  |  |
| *Tectona* *grandis* L. f. |  |  |  |  |  |  | 0.93 | 1.19 | 11.17 | 0.40 | 1.10 | 4.64 |
| *Terminalia* *chebula* Retz. |  |  |  |  |  |  | 0.73 | 1.26 | 10.58 | 0.80 | 2.45 | 9.08 |
| *Toona* *ciliata* M. Roem | 1.20 | 0.11 | 23.47 |  |  |  |  |  |  | 1.27 | 3.86 | 12.84 |
| *Trema* *orientalis* (L.) Blume | 0.33 | 0.01 | 6.55 | 0.40 | 0.16 | 6.74 |  |  |  |  |  |  |
| *Wendlandia* *glabrata* DC. | 0.40 | 0.02 | 8.34 | 0.80 | 0.22 | 12.19 |  |  |  |  |  |  |
| *Xylia* *xylocarpa* (Roxb.) Taub. | 0.47 | 0.03 | 8.73 |  |  |  |  |  |  | 0.67 | 1.67 | 7.19 |
| *Xylosma* *longifolia* Clos | 0.87 | 0.03 | 11.20 | 0.33 | 0.21 | 7.037 |  |  |  |  |  |  |

D-Density, BA-Basal area, IVI-Importance Value Index
