# Supplementary material for: Pattern of forest recovery and carbon stock following shifting cultivation in Manipur, North-East India
Source: PLoS One. 2020 Oct 8;15(10):e0239906. doi: 10.1371/journal.pone.0239906 (PMC7544089; doi:10.1371/journal.pone.0239906)
Supplement: S3 Table — (DOCX) [file pone.0239906.s003.docx]

**S3 Table Abundance of species in seedling and sapling stage and pole and mature stage after abandonment**

| **Seedling and Sapling** | | | | **Pole and mature tree** | | | |
| --- | --- | --- | --- | --- | --- | --- | --- |
| UKHRUL | | CHANDEL | | UKHRUL | | CHANDEL | |
| Tree species | Abundance | Tree species | Abundance | Tree species | Abundance | Tree species | Abundance |
| *Schima* *wallichii* Choisy | 3.14 | *Castanopsis* *hystrix* Hook. f. & Thomson ex A. DC. | 5.25 | *Schima* *wallichii* Choisy | 5.71 | *Castanopsis* *hystrix* Hook. f. & Thomson ex A. DC. | 4.40 |
| *Lithocarpus* *dealbata* (Hook. F. & Thomson ex Miq.) Rehder | 2.57 | *Xylosma* *longifolia* Clos | 4.33 | *Castanopsis* *hystrix* Hook. f. & Thomson ex A. DC. | 5.17 | *Schima* *wallichii* Choisy | 4.00 |
| *Leucosceptrum* *canum* Sm. | 2.20 | *Quercus* *serrata* Murray | 3.75 | *Alnus* *nepalensis* D. Don | 4.50 | *Quercus* *serrata* Murray | 3.18 |
| *Phyllanthus* *emblica* L. | 2.20 | *Schima* *wallichii* Choisy | 3.20 | *Callicarpa* *arborea* Roxb. | 3.40 | *Cinnamomum* *zeylanicum* Blume | 2.83 |
| *Syzygium* *cumini* (L.) Skeels | 2.00 | *Toona* *ciliata* M. Roem | 2.80 | *Lithocarpus* *dealbata* (Hook. F. & Thomson ex Miq.) Rehder | 3.40 | *Syzygium* *cumini* (L.) Skeels | 2.60 |
| *Rhus* *chinensis* Mill. | 1.67 | *Elaeocarpus* *floribundus* Blume | 2.75 | *Toona* *ciliata* M. Roem | 3.33 | *Spondias* *pinnata* (L. f.) Kurz | 2.57 |
| *Elaeocarpus* *floribundus* Blume | 1.60 | *Dipterocarpus* *turbinatus* C. F. Gaertn | 2.00 | *Ficus* *racemosa* L. | 3.00 | *Bauhinia* *variegata* L. | 2.50 |
| *Quercus* *serrata* Murray | 1.60 | *Rhus* *chinensis* Mill. | 2.00 | *Lithocarpus* *truncatus* (King ex Hook. f.) Rehder | 3.00 | *Toona* *ciliata* M. Roem | 2.38 |
| *Albizia* *chinensis* (Osbeck) Merr. | 1.50 | *Trema* *orientalis* (L.) Blume | 2.00 | *Elaeocarpus* *floribundus* Blume | 2.60 | *Choerospondias* *axillaris* (Roxb.) B. L. Burtt & A. W. Hill | 2.20 |
| *Antidesma* *acidum* Retz. | 1.50 | *Xylia* *xylocarpa* (Roxb.) Taub. | 2.00 | *Michelia* *champaca* L. | 2.50 | *Chukrasia* *tabularis* A. Juss. | 2.17 |
| *Bischofia* *javanica* Blume | 1.50 | *Bauhinia* *variegata* L. | 1.80 | *Ficus* *hispida* L. f. | 2.33 | *Magnolia* *pterocarpa* Roxb. | 2.17 |
| *Stereospermum* *chelonoides* (L. f.) DC. | 1.50 | *Aralia* *armata* (Wall. ex G. Don) Seem. | 1.67 | *Tectona* *grandis* L. f. | 2.33 | *Baccaurea* *ramiflora* Lour. | 2.00 |
| *Syzygium* *praecox* (Roxb.) Rathakr. & N. C. Nair | 1.50 | *Gmelina* *arborea* Roxb. | 1.67 | *Pyrus* *pashia* Buch.-Ham. Ex D. Don. | 2.14 | *Callicarpa* *arborea* Roxb. | 2.00 |
| *Macaranga* *denticulata* (Blume) Müll. Ang. | 1.33 | *Stereospermum* *chelonoides* (L. f.) DC. | 1.67 | *Spondias* *pinnata* (L. f.) Kurz | 2.00 | *Macaranga* *denticulata* (Blume) Müll. Ang. | 2.00 |
| *Callicarpa* *arborea* Roxb. | 1.25 | *Wendlandia* *glabrata* DC. | 1.67 | *Choerospondias* *axillaris* (Roxb.) B. L. Burtt & A. W. Hill | 1.86 | *Neolamarckia* *cadamba* (Roxb.) Bosser | 1.88 |
| *Trema* *orientalis* (L.) Blume | 1.25 | *Juglans* *regia* L. | 1.50 | *Tetrameles* *nudiflora* R. Br. | 1.80 | *Artocarpus* *chaplasha* Roxb. | 1.86 |
| *Hydnocarpus* *kurzü* (King) Warb. | 1.20 | *Phyllanthus* *emblica* L. | 1.50 | *Terminalia* *citrine* Roxb. ex Fleming | 1.67 | *Docynia* *indica* (Wall.) Decne. | 1.86 |
| *Wendlandia* *glabrata* DC. | 1.17 | *Maniltoa* *polyandra* (Roxb.) Harms. | 1.33 | *Prunus* *ceraseidos* D. Don | 1.60 | *Engelhardtia* *spicata* Lectan ex Blume | 1.83 |
| *Ficus* *palmata* Forssk. | 1.00 | *Saurauia* *roxburghii* Wall. | 1.33 | *Terminalia* *chebula* Retz. | 1.57 | *Stereospermum* *chelonoides* (L. f.) DC. | 1.83 |
| *Lithocarpus* *pachyphyllus* (Kurz) Rehder | 1.00 | *Pinus* *kesiya* Royle ex Gordon | 1.25 | *Albizia* *chinensis* (Osbeck) Merr. | 1.50 | *Bauhinia* *purpurea* L. | 1.75 |
| *Machilus* *gamblei* King ex Hook. f. | 1.00 | *Albizia* *chinensis* (Osbeck) Merr. | 1.00 | *Ficus* *auriculata* Lour. | 1.50 | *Terminalia* *chebula* Retz. | 1.71 |
|  |  | *Bischofia* *javanica* Blume | 1.00 | *Ficus* *maxima* Mill. | 1.50 | *Albizia* *procera* (Roxb.) Benth | 1.67 |
|  |  | *Ficus* *semicordata* Buch.-Ham. ex Sm. | 1.00 | *Gmelina* *arborea* Roxb. | 1.50 | *Sapindus* *mukorossi* Gaertn. | 1.67 |
|  |  | *Macaranga* *denticulata* (Blume) Müll. Ang. | 1.00 | *Baccaurea* *ramiflora* Lour. | 1.44 | *Xylia* *xylocarpa* (Roxb.) Taub. | 1.67 |
|  |  | *Oreocnide* *integrifolia* (Gaudich.) Miq. | 1.00 | *Cinnamomum* *zeylanicum* Blume | 1.43 | *Ficus* *auriculata* Lour. | 1.57 |
|  |  |  |  | *Juglans* *regia* L. | 1.43 | *Ficus* *hispida* L. f. | 1.57 |
|  |  |  |  | *Docynia* *indica* (Wall.) Decne. | 1.38 | *Mimusops* *elengi* L. | 1.57 |
|  |  |  |  | *Engelhardtia* *spicata* Lectan ex Blume | 1.33 | *Tectona* *grandis* L. f. | 1.50 |
|  |  |  |  | *Sapindus* *mukorossi* Gaertn. | 1.33 | *Haldina* *cordifolia* (Roxb.) Ridsdale | 1.29 |
|  |  |  |  | *Meyna* *laxiflora* Robyns | 1.29 | *Ficus* *virens* Aiton | 1.25 |
|  |  |  |  | *Ficus* *semicordata* Buch.-Ham. ex Sm. | 1.25 | *Phoebe* *hainesiana* Brandis | 1.00 |
|  |  |  |  | *Bauhinia* *variegata* L. | 1.20 |  |  |
|  |  |  |  | *Dillenia* *indica* L. | 1.17 |  |  |
