# Supplementary material for: Pattern of forest recovery and carbon stock following shifting cultivation in Manipur, North-East India
Source: PLoS One. 2020 Oct 8;15(10):e0239906. doi: 10.1371/journal.pone.0239906 (PMC7544089; doi:10.1371/journal.pone.0239906)
Supplement: S4 Table — (DOCX) [file pone.0239906.s004.docx]

**S4 Table Allometric models used in the present study with species name in short**

| Species | Short name | Allometric equation | R² | Specific gravity |
| --- | --- | --- | --- | --- |
| *ᵟAlbizia* *chinensis* (Osbeck) Merr. | AlbiChin | √V= -0.07109 + 2.99732 D - 0.26953 √D | 0.966 | 0.30 |
| *ᵟAlbizia* *procera* (Roxb.) Benth | AlbiProc | √V= -0.07109 + 2.99732 D - 0.26953 √D | 0.966 | 0.64 |
| **Alnus* *nepalensis* D. Don | AlnuNepa | AGB = 0.18 D^2.16^*1.32 | 0.869 | - |
| **Antidesma* *acidum* Retz. | AntiAcid | AGB = 0.18 D^2.16^*1.32 | 0.869 | - |
| **Aralia* *armata* (Wall. ex G. Don) Seem. | AralArma | AGB = 0.18 D^2.16^*1.32 | 0.869 | - |
| *ᵟArtocarpus* *chaplasha* Roxb. | ArtoChap | V= 1.65081 - 4.57531 √D + 11.62114 D^2^ | 0.992 | 0.45 |
| **Baccaurea* *ramiflora* Lour. | BaccRami | AGB = 0.18 D^2.16^*1.32 | 0.869 | - |
| *ᵟBauhinia* *purpurea* L. | BauhPurp | √V= -0.07109 + 2.99732 D - 0.26953 √D | 0.949 | 0.72 |
| *ᵟBauhinia* *variegata* L. | BauhVari | √V= -0.07109 + 2.99732 D - 0.26953 √D | 0.949 | 0.70 |
| *ᵟBischofia* *javanica* Blume | BiscJava | √V= -0.00273 + 2.56199 D | 0.951 | 0.72 |
| *ᵟCallicarpa* *arborea* Roxb. | CallArbo | √V= -0.04506 + 2.33446 D | 0.926 | 0.41 |
| *ᵟCastanopsis* *hystrix* Hook. f. & Thomson ex A. DC. | CastHyst | √V= 0.34640 + 3.99269 D - 1.64666 √D | 0.990 | 0.48 |
| **Choerospondias* *axillaris* (Roxb.) B. L. Burtt & A. W. Hill | ChoeAxil | AGB = 0.18 D^2.16^*1.32 | 0.869 | - |
| *ᵟChukrasia* *tabularis* A. Juss. | ChukTabu | V= -0.07559 + 9.23051 D² | 0.999 | 0.59 |
| *ᵟCinnamomum* *zeylanicum* Blume | CinnZeyl | V= 0.14885 - 1.62875 D + 5.93114 D^2^ + 11.73286 D^3^ | 0.976 | 0.43 |
| **Combretum* *acuminatum* Roxb. | CombAcum | AGB = 0.18 D^2.16^*1.32 | 0.869 | - |
| *ᵟDillenia* *indica* L. | DillIndi | √V= 0.05376 + 3.73731 D - 0.79622 √D | 0.976 | 0.70 |
| *ᵟDipterocarpus* *turbinatus* C. F. Gaertn | DiptTurb | √V= -0.4464 + 3.6062 D | 0.973 | 0.61 |
| **Docynia* *indica* (Wall.) Decne. | DocyIndi | AGB = 0.18 D^2.16^*1.32 | 0.869 | - |
| *ᵟElaeocarpus* *floribundus* Blume | ElaeFlor | √V= 0.43483 + 5.72522 D - 2.59907 √D | 0.991 | 0.54 |
| **Engelhardtia* *spicata* Lectan ex Blume | EngeSpic | AGB = 0.18 D^2.16^*1.32 | 0.869 | - |
| **Eurya* *japonica* Thunb. | EuryJapo | AGB = 0.18 D^2.16^*1.32 | 0.869 | - |
| *ᵟFicus* *auriculata* Lour. | FicuAuri | √V= 0.03629 + 3.95389 D - 0.84421 √D | 0.981 | 0.49 |
| *ᵟFicus* *hispida* L. f. | FicuHisp | √V= 0.03629 + 3.95389 D - 0.84421 √D | 0.981 | 0.35 |
| *ᵟFicus* *maxima* Mill. | FicuMaxi | √V= 0.03629 + 3.95389 D - 0.84421 √D | 0.981 | 0.39 |
| *ᵟFicus* *palmata* Forssk. | FicuPalm | √V= 0.03629 + 3.95389 D - 0.84421 √D | 0.981 | 0.39 |
| *ᵟFicus* *racemosa* L. | FicuRace | √V= 0.03629 + 3.95389 D - 0.84421 √D | 0.981 | 0.32 |
| *ᵟFicus* *semicordata* Buch.-Ham. ex Sm. | FicuSemi | √V= 0.03629 + 3.95389 D - 0.84421 √D | 0.981 | 0.39 |
| *ᵟFicus* *virens* Aiton | FicuVire | √V= 0.03629 + 3.95389 D - 0.84421 √D | 0.981 | 0.39 |
| **Garcinia* *xanthochymus* Hook. f. Ex T. Anderson | GarcXant | AGB = 0.18 D^2.16^*1.32 | 0.869 | - |
| **Gluta* *usitata* (Wall.) Ding Hou | GlutUsit | AGB = 0.18 D^2.16^*1.32 | 0.869 | - |
| *ᵟGmelina* *arborea* Roxb. | GmelArbo | V= 0.01156 + 0.21230 D + 5.10448 D² | 0.969 | 0.56 |
| **Haldina* *cordifolia* (Roxb.) Ridsdale | HaldCord | AGB = 0.18 D^2.16^*1.32 | 0.869 | - |
| *ᵟHydnocarpus* *kurzü* (King) Warb. | HydnKurz | V= -0.10582 + 0.90158 D + 4.05744 D² | 0.955 | 0.59 |
| **Juglans* *regia* L. | JuglRegi | AGB = 0.18 D^2.16^*1.32 | 0.869 | - |
| **Lannea* *grandis* Engl. | LannGran | AGB = 0.18 D^2.16^*1.32 | 0.869 | - |
| **Leucosceptrum* *canum* Sm. | LeucCanu | AGB = 0.18 D^2.16^*1.32 | 0.869 | - |
| **Lithocarpus* *dealbata* (Hook. F. & Thomson ex Miq.) Rehder | LithDeal | AGB = 0.18 D^2.16^*1.32 | 0.869 | - |
| **Lithocarpus* *pachyphyllus* (Kurz) Rehder | LithPach | AGB = 0.18 D^2.16^*1.32 | 0.869 | - |
| **Lithocarpus* *truncatus* (King ex Hook. f.) Rehder | LithTrun | AGB = 0.18 D^2.16^*1.32 | 0.869 | - |
| **Litsea* *cubeba* (Lour.) Pers. | LitsCube | AGB = 0.18 D^2.16^*1.32 | 0.869 | - |
| **Litsea* *glutinosa* (Lour.) C. B. Rob. | LitsGlut | AGB = 0.18 D^2.16^*1.32 | 0.869 | - |
| *ᵟMacaranga* *denticulata* (Blume) Müll. Ang. | MacaDent | √V= -0.10251 + 1.67509 D – 0.31323 D² | 0.790 | 0.33 |
| **Machilus* *gamblei* King ex Hook. f. | MachGamb | AGB = 0.18 D^2.16^*1.32 | 0.869 | - |
| **Magnolia* *hodgsonü* (Hook. f. & Thomson) H. Keng | MagnHodg | AGB = 0.18 D^2.16^*1.32 | 0.869 | - |
| **Magnolia* *pterocarpa* Roxb. | MagnPter | AGB = 0.18 D^2.16^*1.32 | 0.869 | - |
| **Maniltoa* *polyandra* (Roxb.) Harms. | ManiPoly | AGB = 0.18 D^2.16^*1.32 | 0.869 | - |
| *ᵟMelia* *azedarach* L. | MeliAzed | V= -0.03510 + 5.32981 D² | 0.941 | 0.56 |
| **Meyna* *laxiflora* Robyns | MeynLaxi | AGB = 0.18 D^2.16^*1.32 | 0.869 | - |
| *ᵟMichelia* *champaca* L. | MichCham | √V= 0.37142 + 5.64184 D - 2.27448 √D | 0.988 | 0.59 |
| **Mimusops* *elengi* L. | MimuElen | AGB = 0.18 D^2.16^*1.32 | 0.869 | - |
| **Neolamarckia* *cadamba* (Roxb.) Bosser | NeolCada | AGB = 0.18 D^2.16^*1.32 | 0.869 | - |
| **Oreocnide* *integrifolia* (Gaudich.) Miq. | OreoInte | AGB = 0.18 D^2.16^*1.32 | 0.869 | - |
| **Phoebe* *hainesiana* Brandis | PhoeHain | AGB = 0.18 D^2.16^*1.32 | 0.869 | - |
| **Phyllanthus* *emblica* L. | PhylEmbl | AGB = 0.18 D^2.16^*1.32 | 0.869 | - |
| *ᵟPinus* *kesiya* Royle ex Gordon | PinuKesi | V= -0.01523 + 5.65779 D^2^ | 0.976 | 0.43 |
| **Prunus* *ceraseidos* D. Don | PrunCera | AGB = 0.18 D^2.16^*1.32 | 0.869 | - |
| **Pyrus* *pashia* Buch.-Ham. Ex D. Don. | PyruPash | AGB = 0.18 D^2.16^*1.32 | 0.869 | - |
| *ᵟQuercus* *serrata* Murray | QuerSerr | V= 0.14153 - 2.27358 D + 12.90490 D² | 0.983 | 0.69 |
| **Rhus* *chinensis* Mill. | RhusChin | AGB = 0.18 D^2.16^*1.32 | 0.869 | - |
| **Salix* *tetrasperma* Roxb. | SaliTetr | AGB = 0.18 D^2.16^*1.32 | 0.869 | - |
| **Sapindus* *mukorossi* Gaertn. | SapiMuko | AGB = 0.18 D^2.16^*1.32 | 0.869 | - |
| **Saurauia* *roxburghii* Wall. | SaurRoxb | AGB = 0.18 D^2.16^*1.32 | 0.869 | - |
| *ᵟSchima* *wallichii* Choisy | SchiWall | √V= 0.27609 – 3.68443 D + 15.86687 D² | 0.984 | 0.51 |
| *ᵟSpondias* *pinnata* (L. f.) Kurz | SponPinn | √V= 0.49487 + 6.18662 D - 2.95076 √D | 0.973 | 0.31 |
| *ᵟSterculia* *villosa* Roxb. | SterVill | V= 0.27909 - 3.26515 D + 13.46829 D² | 0.990 | 0.26 |
| *ᵟStereospermum* *chelonoides* (L. f.) DC. | SterChel | √V= 0.49746 + 5.98454 D - 2.84986 √D | 0.992 | 0.62 |
| *ᵟSyzygium* *cumini* (L.) Skeels | SyzyCumi | √V= -0.05923 + 2.33654 D | 0.927 | 0.76 |
| *ᵟSyzygium* *praecox* (Roxb.) Rathakr. & N. C. Nair | SyzyPrae | V= -0.13284 + 1.88944 D - 4.96385 D² + 21.41051 D³ | 0.935 | 0.69 |
| **Tamarindus* *indica* L. | TamaIndi | AGB = 0.18 D^2.16^*1.32 | 0.869 | - |
| *ᵟTectona* *grandis* L. f. | TectGran | √V= -0.07109 + 2.99732 D - 0.26953 √D | 0.996 | 0.72 |
| **Terminalia* *chebula* Retz. | TermCheb | AGB = 0.18 D^2.16^*1.32 | 0.869 | - |
| *ᵟTerminalia* *citrine* Roxb. ex Fleming | TermCitr | √V= -0.05527 + 2.59229 D | 0.990 | 0.71 |
| *ᵟTetrameles* *nudiflora* R. Br. | TetrNudi | V= 0.12914 - 2.50478 D + 15.25108 D² | 0.927 | 0.29 |
| *ᵟToona* *ciliata* M. Roem | ToonCili | V= 1.10314 - 3.52579 √D + 15.50182 D² | 0.980 | 0.42 |
| **Trema* *orientalis* (L.) Blume | TremOrie | AGB = 0.18 D^2.16^*1.32 | 0.869 | - |
| **Wendlandia* *glabrata* DC. | WendGlab | AGB = 0.18 D^2.16^*1.32 | 0.869 | - |
| **Xylia* *xylocarpa* (Roxb.) Taub. | XyliXylo | AGB = 0.18 D^2.16^*1.32 | 0.869 | - |
| **Xylosma* *longifolia* Clos | XyloLong | AGB = 0.18 D^2.16^*1.32 | 0.869 | - |

Source: *ᵟ* FSI (1996), ***Nath et al. (2019)
