# Supplementary material for: Pattern of forest recovery and carbon stock following shifting cultivation in Manipur, North-East India
Source: PLoS One. 2020 Oct 8;15(10):e0239906. doi: 10.1371/journal.pone.0239906 (PMC7544089; doi:10.1371/journal.pone.0239906)
Supplement: S5 Table — (DOCX) [file pone.0239906.s005.docx]

**S5 Table Uncertainty percentage for the parameters of TLWBC in all fallow age**

| Site | Parameters estimate | 5 years fallow | | | 10 years fallow | | | 15 years fallow | | | 20 years fallow | | |
| --- | --- | --- | --- | --- | --- | --- | --- | --- | --- | --- | --- | --- | --- |
|  |  | AGBC | BGBC | TLWBC | AGBC | BGBC | TLWBC | AGBC | BGBC | TLWBC | AGBC | BGBC | TLWBC |
| UKHRUL | Uncertainty % | 78.28 | 77.80 | 78.20 | 16.70 | 16.60 | 16.68 | 15.45 | 15.36 | 15.44 | 8.81 | 8.79 | 8.81 |
| CHANDEL | Uncertainty % | 56.25 | 56.47 | 56.29 | 16.08 | 15.97 | 16.06 | 10.84 | 10.87 | 10.84 | 11.09 | 10.91 | 11.05 |

TLWBC-Total living woody biomass carbon, AGBC-Aboveground biomass carbon, BGBC-Belowground biomass c arbon.
