# Supplementary material for: Pattern of forest recovery and carbon stock following shifting cultivation in Manipur, North-East India
Source: PLoS One. 2020 Oct 8;15(10):e0239906. doi: 10.1371/journal.pone.0239906 (PMC7544089; doi:10.1371/journal.pone.0239906)
Supplement: Attachment — Submitted filename: Review for Manuscript Number PONE-D-19-29248.docx [file pone.0239906.s006.docx]

PATTERN OF FOREST RECOVERY AND CARBON STOCK FOLLOWING SHIFTING CULTIVATION IN MANIPUR, NORTH-EAST INDIA

Pentile Thong^1,@^, Uttam Kumar Sahoo^1^, Uttam Thangjam^1,@^ and Rocky Pebam^2,@^

^1^Department of Forestry, Mizoram University, Aizawl, Mizoram – 796004

^2^North-Eastern Space Applications Centre, Umiam, Meghalaya - 793103

Corresponding Author: Uttam Kumar Sahoo, email: [uttams64@gmail.com](mailto:uttams64@gmail.com); [uksahoo_2003@rediffmail.com](mailto:uksahoo_2003@rediffmail.com)

@These authors contributed equally to the work

**Abstract**

Shifting cultivation has resulted in large-scale deforestation and forest degradation in the tropics, however the fallows after abandonment are considered as a major global carbon sink. The paper is an attempt to determine the forest recovery patterns following shifting cultivation by evaluating the tree species composition, diversity and abundance with respect to topographical factors in Manipur, India. We also used ordination analysis to understand the change in species composition with regard to environmental variables. The living woody biomass carbon of each fallow was quantified, and the factors affecting the recovery of carbon stock along an increasing fallow gradient was assessed. Our results showed that the species richness and basal area recovered relatively with time since abandonment, while the species composition recovery showed resemblance between the fallows of similar age. The north-facing lower elevation fallow sites displayed higher species richness and stem density than those in higher elevations which supports our hypothesis that topographical parameters namely slope, aspect and elevation have a bearing on the species composition with increasing fallow age. Environmental variables had no impact on the regeneration of *Elaeocarpus* *floribundus* Blume and *Castanopsis* *hystrix* Hook. f. & Thomson ex A. DC. for this to suggest that they may be capable of effective restoration of degraded forest areas. As these species appear naturally in the forests, it would facilitate quicker rehabilitation and reinstate the soil nutrients making the soil reusable in a short term, such that the expansion of slashing and burning of primary forests for cultivation would be reduced. We also found that fallow age plays a vital role in recovering above-ground biomass carbon from living woody species followed by the aspect of the site.

**KEYWORDS:** fallows, carbon sink, ordination analysis, restoration, above-ground biomass carbon

**Introduction**

Shifting cultivation is a major land use in tropics despite the fact that it is blamed to be a major cause of forest loss. Serious concerns are raised that this practice though the oldest form of agriculture may negatively affect biodiversity, carbon stocks and greenhouse gas emissions [1-3]. However, this practice provides subsistence livelihoods to millions of people worldwide [1] and, therefore, it is likely to continue as it is intricately linked to cultural, ecological and economic aspects of communities [4]. This practice is characterized by the alternation of the cropping and fallow phase, while the abandoned land regenerates naturally. After a certain fallow period, the trees are slashed and burnt for the ashes to enrich the soil, thus allowing a new cropping phase. This intervening period between two successive slashed and burnt practices is termed as *Jhum cycle*. The fallow duration and cropping period are specifically influenced by ecological and socio-economic factors [5]. In North-East India, this practice is locally known as Jhum where the cropping period has been reported to be 1-2 years with a fallow duration varying from 6-12 years [6-7]. The regional estimation of abandoned land after Jhum differs significantly depending on the means of estimation. However, this region encompasses 3697.14 km² of the National area of abandoned land [8]. Manipur, in particular, has 0.45% (100.10 km²) of its total geographical area under abandoned Jhum land. Under the natural process of ecological recovery, the abandoned lands are eventually restored to secondary forests.

In the tropical region, secondary forests constitute over 50% of the forested area and define a major global carbon sink which stores carbon in above-ground biomass [9]. In India, secondary forests cover about 32 million ha which is approximately 45.8% of the forest area of the country [10]. The dynamic nature of shifting cultivation results in a landscape mosaic of Jjhum field, secondary forests and old-growth forests. As such, many forests in Northeast India are secondary forests at different stages of succession following shifting cultivation. These forests are an essential source of rural livelihood and also for multiple environmental functions such as soil and watershed conservation, flood control and carbon storage [11]. However, secondary forests get constantly subjected to increasing exploitation by the growing population as well as by budding industrial and urban demand for forest products. Succession following Jhum is faster in terms of basal area, regeneration and accumulation of species than other human-modified and agroforestry systems in the tropics and the diversity of woody species steadily increase with the age of fallow [12-13]. The quantity of biomass in a forest ascertains the potential amount of carbon added to the atmosphere or sequestered on the land [14]. The carbon stock in tree biomass from young growing forests contributes about 50% to atmospheric carbon and is hence considered as the greatest terrestrial carbon sink. An understanding of species recovery especially woody species and carbon stock is vital not only for developing rehabilitation strategy for shifting cultivation areas but also for carbon-based payments for ecosystem services such as reducing emissions from deforestation and forest degradation.

Given the importance of the secondary forests and the human pressure on these resources, the present study is conducted to (1) understand the tree recovery pattern following shifting cultivation, (2) estimate the living woody biomass and carbon stock under different fallow regimes, (3) identify naturally occurring potential tree species to accelerate the restorative phase of the fallows. Despite the undulated topography, the state of Manipur has 77.69% of its total geographical area under forest cover, based on which we put forward the hypothesis that topographical parameters namely slope, aspect and elevation have a bearing on the species composition with increasing fallow age. We believe that the result of this study will provide baseline data on the rate of vegetation recovery and biomass carbon as accumulated by the living woody species in order to achieve proper management of such successive areas.

**Materials and methods**

**Ethics statement:**

The study was carried out in four different shifting cultivation fallow lands owned by villagers in Ukhrul and Chandel district of Manipur. Permission was obtained from each of the land owner towards collection of plant samples and vegetation data. No rare or endangered species was used in this study, and besides the study did neither involve the use of wild animals nor threatened environmental systems.

**Study area:**

The study was conducted in Ukhrul (24°29’6’’ - 24°41’35”-N and 94°7’24” - 94°44’44”E) and Chandel (23°50’11” - 24°38’52”N and 93°53’58” - 94°18’10”E) districts of Manipur, Northeast India (Fig1). Ukhrul is located at an elevation of 1662 m a.s.l and Chandel at 957 m a.s.l. The subtropical climate of this region is affected by the southwest and northeast monsoons with distinct rainy (June to September) and dry (November to February) seasons and also by an annual rainfall of about 1616 mm and 1877 mm at Ukhrul and Chandel respectively. The average annual temperature of 15.5°C in Ukhrul and 20.6°C in Chandel contributes to its warm and temperate climatic condition. Ukhrul and Chandel districts have contributed to the increase in the net forest cover of the state by 151 km² and 17 km² respectively [15].

**Site selection:**

Keeping in view elevation, soil type, and mean annual rainfall and temperature, we selected four different fallow periods after the abandonment of the Jhum fields, namely, 5 (JF_5_), 10 (JF_10_), 15 (JF_15_) and 20 (JF_20_) years fallow (Fig 1a-1b). The study sites were selected as per the findings of [7] and the plot was navigated using a GPS. The elevation of the study sites in Ukhrul ranges between 995-1537m a.s.l and 398-1247 m a.s.l in Chandel. Majority of the fallow lands were confined to 21 – 30° slope and aspect of North facing (20.8%) followed by South (16.7%).

**Figure 1: (a) Map showing the location of the study area. (b) Fallow polygons (derives from post-classification of satellite imagery) placed over Google Earth in the study areas.**

**Vegetation sampling:**

The vegetation sampling was conducted during the dry months of January and February for fallow aged 5 (JF_5_), 10 (JF_10_), 15 (JF_15_) and 20 (JF_20_) years. We identified a total of 12 sites (four fallow aged plots x three replicates) in each district. At each site, five quadrates of 10 m x 10 m were randomly laid for identifying tree species at different stages of their growth. The diameter at breast height (dbh) was measured at 1.37 m from the ground level for individuals with dbh > 3 cm. Mature trees were defined as stems with dbh ≥10 cm and height ˃2 m, poles as individuals with dbh ≥ 3 to ˂10 cm and height ˃ 2m, saplings as individuals with collar diameter ˂ 3cm and height ˃ 30 cm to ≤ 2 m, and seedlings as individuals with collar diameter ˂ 3cm and height up to 30 cm. The herbaria at Mizoram University and other published literatures were consulted for correct tree species identification for those which could not be identified in the field. We referred to The Plant List (www.theplantlist.org) for species nomenclature classification.

**Analysis of plant diversity and community structure:**

Community structure variables such as density, frequency, abundance, basal area and important value index (IVI) of each species in different fallow sites were calculated according to Mueller-Dombois and Ellenberg [16]. Spatial distribution of tree species was determined following Whitford Index [17]. Plant diversity measures such as Shannon-Wiener diversity index (H'), Simpson’s dominance index (C), Pielou’s evenness index (J) and Margalef species richness index (D_mg_) were also determined [18-21].

Rarefaction curves based on the number of individuals encountered during the vegetation analysis was plotted to assess the species accumulation pattern for all tree species. The rate of change in species composition with increasing fallow age (β diversity) was calculated following Whittaker [22]. To analyse the similarity in species composition among the replicates of different fallow age stands, Jaccard’s similarity index was represented in the form of a dendrogram [23].

**Biomass inventory:**

A non-destructive sampling method was adopted to estimate tree above and below-ground biomass and carbon stock of each tree species. With dbh as an independent variable, tree volume was calculated using local and regional based species specific volume equations [24]. Specific gravity of the species was obtained from Global wood density database [25]. The above-ground biomass (AGB) of each individual tree (in kg) was calculated as:

AGB = Tree volume x Specific gravity

However, for those species for which volume equations were not available, pan-tropic equations for mixed species was used to calculate the AGB as given by Intergovernmental Panel on Climate Change [26]:

AGB = exp (-2.289 + 2.649 logD - 0.021 logD²)

Where D is the dbh in cm

The AGB of saplings and seedlings (collar diameter ˂ 3 cm) was derived by using the equation suggested by [27]:

AGB = 3.344 + 0.443 LnD²

The above-ground carbon stock was calculated as 47% of the AGB as summarized by IPCC [28] and below-ground carbon stock was determined by the equation given by [29]:

BGBC = 0.205 x AGB

Hence, the total of living woody biomass carbon was calculated as:

TLWBC = AGBC + BGBC

**Statistical analysis:**

All statistical analysis was conducted using SPSS version 20.0 and PAST version 3.25. One-way ANOVA (Analysis of Variance) was used to examine if there is a significant difference in stem density, basal area, diversity, evenness, dominance and species richness (dependent variables) among different fallow ages (fixed variable). Multiple regression was performed to investigate the relationship of slope, aspect and altitude with species composition along an increasing fallow gradient. The constrained ordination technique of Canonical Correspondence Analysis (CCA) was used to assess the environmental variables contributing to the variation in species composition among fallows. The environmental variables used in CCA were (i) elevation (ii) fallow age (iii) slope, and (iv) aspect. Each of these environmental variables and its association with the variation in the distribution of species were tested using 999 unrestricted permutations.

Prior to analysis of carbon stock, the normality and heteroscedasticity of carbon distribution with increasing fallow age was checked using the Shapiro-Wilk and Levene’s test respectively. To assess variation in carbon stock, we constructed a linear mixed-effect model (LMEM) with fallow age, slope, aspect and elevation as fixed effects, and incorporated sites nested within fallow ages as random intercepts in the model. We applied the information theoretical approach based on the Akaike Information Criterion corrected for small sample sizes (AICc) for model selection, and the model with the lowest AICc score was selected as the best-fit model.

**Results**

**Stand characteristic and tree diversity:**

A total of 58 tree species belonging to 27 families in Ukhrul and 59 tree species belonging to 31 families in Chandel were recorded from the fallow stands. The most dominant family in the study areas was Fagaceae except for the 15 years old fallow site specific to Chandel where the dominant family was Pinaceae. ANOVA results showed that stem density, basal area, species diversity and species richness significantly increased with increasing fallow age (Table 1). However, no significant outcome was observed in the species dominance and evenness index in Ukhrul and Chandel respectively. The rarefaction curves of species accumulation indicated that the tree species richness curves in all the fallow ages reached a plateau based on the increasing fallow age. The highest number of individuals was recorded from the 20 years fallow with 404 individuals in Ukhrul and 449 in Chandel (Fig 2). Whittaker’s β index showed a high level of species similarity between 5 years and 10 years fallow in Ukhrul as well as between 15 years and 20 years fallow in Chandel (Table 2). The three replicates of 5 years fallow, 10 years fallow, 15 years fallow and 20 years fallow were dissimilar to each other with regard to species composition in Ukhrul, while on the contrary it was not so in Chandel (Fig 3). The 15 years fallow replicates were most similar to each other in both the districts. In Chandel, the younger fallows were more similar to each other and displayed increasing similarity in species composition along an increasing fallow gradient. However, in Ukhrul, 15 and 20 years fallows were more similar to each other than the younger fallows, and the similarity in species composition declined from 20 years to 5 years fallow.

**Figure 2: Rarefaction curves of species accumulation in fallows following shifting cultivation.**

**Figure 3: Dendrogram based on similarities in composition of trees in different replicates of jhum fallows. The values ranges from 0 (complete similarity) to 1(complete dissimilarity).**

Table 1: Phytosociological and diversity attributes of tree species in the fallow stands in the study area.

| **Parameters** | **UKHRUL** | | | | | | |
| --- | --- | --- | --- | --- | --- | --- | --- |
|  | **JF₅** | **JF₁₀** | **JF₁₅** | **JF₂₀** | ***R²*** | ***F*** | ***P*** |
| No. of species | 21 | 30 | 34 | 34 | - | - | - |
| No. of families | 15 | 18 | 20 | 21 | - | - | - |
| Density (individuals/ha) | 1233.33±42.16 | 1900±75.59 | 2286.67±88.3 | 2693.33±73.33 | 0.78 | 212.05 | <0.001** |
| Basal area (m²haˉ¹) | 0.85±0.18 | 9.1±0.48 | 29.26±1.59 | 82.1±2.75 | 0.84 | 304.16 | <0.001** |
| Shannon Diversity Index (H´) | 1.72±0.07 | 1.97±0.11 | 1.84±0.14 | 2.28±0.09 | 0.13 | 9.68 | 0.002* |
| Pielou Evenness Index (J´) | 0.87±0.01 | 0.84±0.03 | 0.79±0.03 | 0.82±0.02 | 0.05 | 4.42 | 0.039* |
| Simpson Dominance Index (C) | 0.21±0.02 | 0.18±0.03 | 0.23±0.04 | 0.13±0.01 | 0.03 | 2.82 | 0.09^NS^ |
| Margalef Species Richness Index (D_mg_) | 2.24±0.16 | 2.81±0.3 | 2.52±0.31 | 3.48±0.29 | 0.1 | 7.71 | 0.007* |
| **Parameters** | **CHANDEL** | | | | | | |
|  | **JF₅** | **JF₁₀** | **JF₁₅** | **JF₂₀** | ***R²*** | ***F*** | ***P*** |
| No. of species | 25 | 26 | 29 | 31 | - | - | - |
| No. of families | 20 | 16 | 22 | 20 | - | - | - |
| Density (individuals/ha) | 1493.33±39.6 | 2020±31.17 | 2526.67±65.8 | 2993.33±59.73 | 0.89 | 494.33 | <0.001** |
| Basal area (m²haˉ¹) | 1.22±0.12 | 7.97±0.33 | 32.1±1.19 | 82.43±3.52 | 0.84 | 308.48 | <0.001** |
| Shannon Diversity Index (H´) | 1.61±0.11 | 1.83±0.08 | 1.88±0.15 | 2.44±0.11 | 0.27 | 22.82 | <0.001** |
| Pielou Evenness Index (J´) | 0.87±0.02 | 0.88±0.01 | 0.81±0.03 | 0.91±0.02 | 0.01 | 0.41 | 0.52^NS^ |
| Simpson Dominance Index (C) | 0.25±0.03 | 0.19±0.02 | 0.22±0.04 | 0.11±0.01 | 0.13 | 10.15 | 0.002* |
| Margalef Species Richness Index (D_mg_) | 1.96±0.24 | 2.13±0.19 | 2.43±0.28 | 3.7±0.46 | 0.2 | 15.59 | <0.001** |

Note: **Significant at 0.001, *Significant at 0.05 and ^NS^ Not significant

Table 2: Whittaker’s β index of the fallow stands in the study area.

|  | UKHRUL | | | | CHANDEL | | | |
| --- | --- | --- | --- | --- | --- | --- | --- | --- |
|  | **JF₅** | **JF₁₀** | **JF₁₅** | **JF₂₀** | **JF₅** | **JF₁₀** | **JF₁₅** | **JF₂₀** |
| **JF₅** | 0 | 0.33 | 0.60 | 0.82 | 0 | 0.41 | 0.74 | 0.71 |
| **JF₁₀** |  | 0 | 0.41 | 0.63 |  | 0 | 0.71 | 0.79 |
| **JF₁₅** |  |  | 0 | 0.44 |  |  | 0 | 0.33 |
| **JF₂₀** |  |  |  | 0 |  |  |  | 0 |

**Population density and distribution pattern:**

The distribution analysis of all tree species encountered in each fallow stand is represented in Appendix 1. The dominance-distribution curves for all the fallow stands showed normal distribution with high dominance and low equitability in younger fallows which proceed to high equitability as the fallow age increases (Fig 4). In Ukhrul, *Lithocarpus dealbatus* (Hook.f. & Thomson ex Miq.) Rehder (IV1 29.32), *Rhus chinenesis* Mill. (IVI 27.85), *Pinus kesiya* Royle ex Gordon (IVI 35.73) and *Schima wallichii* Choisy (IVI 23.59) were the most dominant species along an increasing fallow gradient, while *Quercus serrata* Murray (IVI 24.26, 26.82) dominated in 5 and 10 years fallow in Chandel and *Schima wallichii* Choisy (IVI 35.25) and *Castanopsis hystrix* Hook. F. & Thomson ex A. DC. (IVI 24.76) dominated the 15 and 20 years fallow respectively. Most of the fallows showed contigious distribution pattern (32-90%) followed by random distribution (10-68%). Regular distribution was observed only for 20 years fallow in Chandel (Fig 5).

**Figure 4: Dominance-diversity curves for tree species at different fallow age.**

**Figure 5: Spatial distribution of tree species at different fallow age.**

**Per cent recovery of tree species:**

In Ukhrul, a distinct rise of 42.9 % in the count of tree species was observed from 5 years fallow to 10 years fallow. It further increased by 3.3% in 15 years fallow and then remained stable up to 20 years fallow. Likewise in Chandel, the count of tree species increased by 4% from 5 years fallow to 10 years fallow and which was further enhanced by 11.5% in 15 years fallow and 6.9% in 20 years fallow. Hence, the percent recovery of tree species from 5 years fallow to 20 years fallow was 61.9% and 34.78% in Ukhrul and Chandel respectively.

**Influence of topographical parameters on the species composition:**

ANOVA results showed that species richness increases significantly from the youngest to the oldest fallow in Ukhrul (*F*=13.17, *P*˂0.001) as well as in Chandel (*F*=13.28, *P*˂0.001). Multiple regression results revealed that topographical factors such as slope, aspect and elevation, all had a significant impact on the species richness and stem density along an increasing fallow gradient (Table 3). This finding supports our proposed hypothesis that slope, aspect and elevation have a bearing on the species composition in successional areas.

Table 3: Relationship of jhum fallows with slope, aspect and elevation

|  |  | UKHRUL | | | CHANDEL | | |
| --- | --- | --- | --- | --- | --- | --- | --- |
|  |  | R² | F | P | R² | F | P |
| Species  Richness | JF x Slope | 0.28 | 10.86 | <0.001** | 0.32 | 13.53 | <0.001** |
|  | JF x Aspect | 0.26 | 9.89 | <0.001** | 0.31 | 12.98 | <0.001** |
|  | JF x Elevation | 0.26 | 9.87 | <0.001** | 0.31 | 12.87 | <0.001** |
| Stem  Density | JF x Slope | 0.79 | 105.64 | <0.001** | 0.89 | 242.91 | <0.001** |
|  | JF x Aspect | 0.79 | 110.21 | <0.001** | 0.90 | 244.93 | <0.001** |
|  | JF x Elevation | 0.79 | 104.35 | <0.001** | 0.90 | 248.81 | <0.001** |

Note: **Significant at 0.001

**Species affected by environmental variables:**

The contribution of environmental variables in the distribution and abundance of tree species along an increasing fallow gradient was determined by Canonical Correspondence Analysis (CCA). Fig 6a shows that the abundance of *Toona ciliata* M. Roem and *Prunus ceraseidos* D. Don were closely associated with the elevation of the sites in Ukhrul, while *Pinus kesiya* Royle ex Gordon and *Toona ciliata* M. Roem were associated with the slope. The fallow age as well as the aspect of the sites also influenced the abundance of *Spondias pinnata* (L. f.) Kurz and *Terminalia citrine* Roxb. ex Fleming. In Chandel, the species mostly associated with the slope and aspect of the sites were *Dipterocarpus turbinatus* C. F. Gaertn and *Gmelina arborea* Roxb. (Fig 6b). Species namely *Cinnamomum zeylanicum* Blume, *Bauhinia purpurea* L. and *Spondias pinnata* (L. f.) Kurz, *Sapindus mukorossi* Gaertn. were associated with the elevation and fallow age simultaneously.

**Potential species for restoration:**

ANOVA results showed that the species composition of tree seedling and sapling decreases significantly from the youngest to the oldest fallow in Ukhrul (*F*=136.81, *P*˂0.001) and Chandel (*F*=134.84, *P*˂0.001) while the pole and mature tree species composition increases from the youngest to the oldest fallow in Ukhrul (*F*=28.63, *P*˂0.001) and Chandel (*F*=22.70, *P*˂0.001) as well. Some species establishes rapidly after abandonment and continues to grow to mature trees in older fallows. One such species is *Elaeocarpus* *floribundus* Blume in Ukhrul and *Castanopsis* *hystrix* Hook. f. & Thomson ex A. DC. in Chandel (Appendix 3). The CCA triplots further showed that these species (ElaeFlor and CastHyst) were independent of the environmental variables (Figs 7 and 8), that is, they appear opposite to the direction of increasing value of the environmental variables and its relation with the tree species. This testifies that the elevation, fallow age, slope and aspect of the sites have no influence on the abundance of *Elaeocarpus* *floribundus* Blume and *Castanopsis* *hystrix* Hook. f. & Thomson ex A. DC. in Ukhrul and Chandel respectively.

**Figure 6: CCA ordination diagram showing distribution and abundance of tree species in (a) Ukhrul where Trace=0.799 and p=0.001. The axis 1 accounts for 68.6% and axis 2 accounts for 19.8% of the variability. (b) Chandel where Trace=0.541 and p=0.001. The axis 1 accounts for 77.3% and axis 2 accounts for 14.4% of the variability. Full species names are shown in appendix 2.**

**Figure 7: CCA triplot of tree species in (i) seedling and sapling stage, and (ii) pole and mature stage in Ukhrul from 5 years, 10 years, 15 years and 20 years fallow following shifting cultivation and its relation with selected environmental variables. Full species names are shown in appendix 2.**

**Figure 8: CCA triplot of tree species in (i) seedling and sapling stage, and (ii) pole and mature stage in Chandel from 5 years, 10 years, 15 years and 20 years fallow following shifting cultivation and its relation with selected environmental variables. Full species names are shown in appendix 2.**

**Tree above-ground biomass and total carbon stock:**

The biomass and carbon stock of 79 living woody tree species were evaluated from species specific allometric models (Appendix 2). Total living woody biomass carbon (AGBC+BGBC) ranged from 0.11 Mg ha^-1^ to 5.62 Mg ha^-1^ in Ukhrul and 0.05 Mg ha^-1^ to 5.80 Mg ha^-1^ in Chandel. The total carbon stock increased rapidly with increased time since abandonment (Fig 9).

**Figure 9: Total living woody biomass carbon estimates for Jhum fallow.**

**Species contribution to carbon stock:**

In Ukhrul, *Syzygium praecox* (Roxb.) Rathakr. & N. C. Nair contributed the highest total living woody biomass carbon (TLWBC) in 5 and 15 year old fallows, followed by *Rhus chinensis* Mill. and *Cimmamomum zeylanicum* Blume in 10 and 20 year old fallows respectively. The highest TLWBC in Chandel was contributed by *Aralia armata* (Wall. ex G. Don) Seem. in 5 year old fallow followed by *Lithocrpus pachyphyllus* (Kurz) Rehder, *Elaeocarpus* *floribundus* Blume and *Cimmamomum zeylanicum* Blume in 10, 15 and 20 year old fallow respectively (Table 4).

Table 4: Summary of biomass carbon contributed by individual species in each fallow age

| **Tree species** | **Living Woody Biomass Carbon (Mg haˉ¹)** | | | | | | | |
| --- | --- | --- | --- | --- | --- | --- | --- | --- |
|  | **Ukhrul** | | | | **Chandel** | | | |
|  | **JF₅** | **JF₁₀** | **JF₁₅** | **JF₂₀** | **JF₅** | **JF₁₀** | **JF₁₅** | **JF₂₀** |
| Albizia chinensis (Osbeck) Merr. | 0.00057 | 0.00424 |  | 0.026 | 0.00128 | 0.007 |  |  |
| Albizia procera (Roxb.) Benth |  |  |  |  |  |  | 0.029 | 0.129 |
| Alnus nepalensis D. Don |  | 0.06225 | 0.191 | 0.491 |  |  |  |  |
| Antidesma acidum Retz. | 0.01810 | 0.03190 |  |  |  |  |  |  |
| Aralia armata (Wall. ex G. Don) Seem. |  |  |  |  | 0.01629 |  |  |  |
| Artocarpus chaplasha Roxb. |  |  |  |  |  |  | 0.033 | 0.180 |
| Baccaurea ramiflora Lour. |  |  | 0.067 | 0.224 |  |  | 0.100 | 0.200 |
| Bauhinia purpurea L. |  |  | 0.140 | 0.042 |  |  |  | 0.095 |
| Bauhinia variegata L. |  | 0.03056 |  | 0.116 | 0.00023 |  | 0.088 | 0.274 |
| Bischofia javanica Blume | 0.00254 | 0.00450 |  |  | 0.00008 | 0.018 |  |  |
| Callicarpa arborea Roxb. | 0.00128 |  |  | 0.104 |  | 0.007 | 0.017 | 0.074 |
| Castanopsis hystrix Hook. f. & Thomson ex A. DC. |  | 0.02718 | 0.068 | 0.567 | 0.00243 | 0.051 |  | 0.672 |
| Choerospondias axillaris (Roxb.) B. L. Burtt & A. W. Hill |  |  |  | 0.226 |  |  | 0.092 | 0.211 |
| Chukrasia tabularis A. Juss. |  |  |  |  |  |  | 0.035 | 0.147 |
| Cinnamomum zeylanicum Blume |  |  | 0.348 | 2.783 |  |  |  | 3.296 |
| Combretum acuminatum Roxb. |  | 0.07206 | 0.267 |  |  |  |  |  |
| Dillenia indica L. |  |  |  | 0.193 |  |  |  |  |
| Dipterocarpus turbinatus C. F. Gaertn |  |  |  |  | 0.00564 |  |  |  |
| Docynia indica (Wall.) Decne. |  |  | 0.057 | 0.204 |  |  | 0.046 | 0.227 |
| Elaeocarpus floribundus Blume | 0.00410 | 0.06569 | 0.229 | 0.508 | 0.00322 |  | 0.308 |  |
| Engelhardtia spicata Lectan ex Blume |  |  |  | 0.198 |  |  | 0.101 | 0.216 |
| Eurya japonica Thunb. |  |  |  |  |  |  | 0.124 |  |
| Ficus auriculata Lour. |  |  | 0.042 | 0.153 |  | 0.011 | 0.033 | 0.227 |
| Ficus hispida L. f. |  | 0.00541 | 0.027 | 0.081 |  | 0.002 |  | 0.159 |
| Ficus maxima Mill. |  |  |  | 0.045 |  |  |  |  |
| Ficus palmata Forssk. | 0.00102 | 0.00204 |  |  |  |  |  |  |
| Ficus racemosa L. |  | 0.00405 | 0.019 | 0.052 |  |  | 0.051 |  |
| Ficus semicordata Buch.-Ham. ex Sm. |  | 0.00982 | 0.028 | 0.076 | 0.00130 | 0.022 | 0.041 |  |
| Ficus virens Aiton |  |  |  |  |  |  |  | 0.070 |
| Garcinia xanthochymus Hook. f. Ex T. Anderson |  |  |  |  |  |  | 0.136 |  |
| Gluta usitata (Wall.) Ding Hou |  |  |  |  |  | 0.033 |  |  |
| Gmelina arborea Roxb. |  | 0.00959 |  | 0.038 | 0.00133 |  |  |  |
| Haldina cordifolia (Roxb.) Ridsdale |  |  |  |  |  |  | 0.082 | 0.123 |
| Hydnocarpus kurzü (King) Warb. | 0.00014 | 0.00244 |  |  |  |  |  |  |
| Juglans regia L. |  |  |  | 0.202 | 0.00790 |  |  |  |
| Lannea grandis Engl. |  | 0.06246 |  |  |  | 0.062 | 0.243 |  |
| Leucosceptrum canum Sm. | 0.00925 | 0.07591 | 0.050 |  |  |  |  |  |
| Lithocarpus dealbatus (Hook. F. & Thomson ex Miq.) Rehder | 0.01403 | 0.06821 | 0.181 | 0.345 |  |  |  |  |
| Lithocarpus pachyphyllus (Kurz) Rehder | 0.00005 | 0.00919 | 0.130 |  |  | 0.088 | 0.189 |  |
| Lithocarpus truncatus (King ex Hook. f.) Rehder |  |  | 0.071 | 0.070 |  |  |  |  |
| Litsea cubeba (Lour.) Pers. |  |  |  |  |  | 0.016 |  |  |
| Litsea glutinosa (Lour.) C. B. Rob. |  |  | 0.068 |  |  |  |  |  |
| Macaranga denticulata (Blume) Müll. Ang. | 0.00011 |  | 0.022 |  | 0.00296 |  |  | 0.065 |
| Machilus gamblei King ex Hook. f. | 0.00633 |  |  |  |  | 0.039 |  |  |
| Magnolia hodgsonü (Hook. f. & Thomson) H. Keng |  |  | 0.035 |  |  |  |  |  |
| Magnolia pterocarpa Roxb. |  |  |  |  |  |  | 0.217 | 0.237 |
| Maniltoa polyandra (Roxb.) Harms. |  |  |  |  | 0.00683 | 0.012 |  |  |
| Melia azedarach L. |  |  |  |  |  | 0.008 |  |  |
| Meyna laxiflora Robyns |  |  |  | 0.172 |  |  |  |  |
| Michelia champaca L. |  | 0.08259 |  | 0.516 |  |  |  |  |
| Mimusops elengi L. |  |  |  |  |  |  | 0.141 | 0.186 |
| Neolamarckia cadamba (Roxb.) Bosser |  |  |  |  |  |  |  | 0.288 |
| Oreocnide integrifolia (Gaudich.) Miq. |  |  |  |  | 0.00008 | 0.055 |  |  |
| Phoebe hainesiana Brandis |  |  | 0.048 |  |  |  | 0.048 | 0.068 |
| Phyllanthus emblica L. | 0.00029 | 0.07086 | 0.079 |  | 0.00017 | 0.027 | 0.125 |  |
| Pinus kesiya Royle ex Gordon |  |  | 0.138 |  | 0.00127 | 0.005 | 0.152 |  |
| Prunus ceraseidos D. Don |  |  |  | 0.148 |  |  |  |  |
| Pyrus pashia Buch.-Ham. Ex D. Don. |  | 0.03299 | 0.092 | 0.268 |  |  |  |  |
| Quercus serrata Murray | 0.00176 | 0.02301 | 0.151 |  | 0.01187 | 0.073 |  | 0.658 |
| Rhus chinensis Mill. | 0.01959 | 0.15980 |  |  | 0.00633 | 0.055 |  |  |
| Salix tetrasperma Roxb. |  | 0.01432 |  |  |  |  |  |  |
| Sapindus mukorossi Gaertn. |  |  | 0.059 | 0.176 |  |  | 0.125 | 0.181 |
| Saurauia roxburghii Wall. |  |  |  |  | 0.00010 | 0.039 |  |  |
| Schima wallichii Choisy | 0.00131 | 0.01565 | 0.146 | 0.474 | 0.00278 | 0.021 | 0.275 | 0.438 |
| Spondias pinnata (L. f.) Kurz |  |  |  | 0.445 |  |  |  | 0.431 |
| Sterculia villosa Roxb. |  |  |  |  |  | 0.014 |  |  |
| Stereospermum chelonoides (L. f.) DC. | 0.00008 | 0.02927 |  |  | 0.00014 |  | 0.083 | 0.493 |
| Syzygium cumini (L.) Skeels | 0.00042 | 0.01618 | 0.014 |  |  |  |  | 0.093 |
| Syzygium praecox (Roxb.) Rathakr. & N. C. Nair | 0.10582 | 0.00006 | 0.929 |  |  |  |  |  |
| Tamarindus indica L. |  |  |  |  |  | 0.034 |  |  |
| Tectona grandis L. f. |  |  | 0.016 | 0.094 |  |  | 0.092 | 0.087 |
| Terminalia chebula Retz. |  |  | 0.070 | 0.233 |  |  | 0.155 | 0.222 |
| Terminalia citrine Roxb. ex Fleming |  |  |  | 0.184 |  |  |  |  |
| Tetrameles nudiflora R. Br. |  |  | 0.034 | 0.083 |  |  |  |  |
| Toona ciliata M. Roem |  |  |  | 0.394 | 0.00594 |  |  | 0.322 |
| Trema orientalis (L.) Blume | 0.00248 |  |  |  | 0.00254 | 0.026 |  |  |
| Wendlandia glabrata DC. | 0.00306 | 0.05418 | 0.180 |  | 0.00256 | 0.028 |  |  |
| Xylia xylocarpa (Roxb.) Taub. |  |  |  |  | 0.00305 |  |  | 0.171 |
| Xylosma longifolia Clos |  | 0.05484 | 0.082 |  | 0.00031 | 0.041 |  |  |
| AGB | 0.19 | 1.10 | 4.08 | 9.93 | 0.09 | 0.79 | 3.16 | 10.24 |
| AGB Carbon | 0.09 | 0.52 | 1.92 | 4.67 | 0.04 | 0.37 | 1.48 | 4.81 |
| BGB Carbon | 0.02 | 0.11 | 0.39 | 0.96 | 0.01 | 0.08 | 0.30 | 0.99 |
| **Total Carbon** | **0.11** | **0.62** | **2.31** | **5.62** | **0.05** | **0.45** | **1.79** | **5.80** |

**Factors affecting the recovery of carbon stock:**

The fallow age was found to have contributed the most to the recovery of carbon from living woody biomass in Ukhrul and Chandel as well (Table 5). It was also observed that the aspect and slope of the sites in Ukhrul were important to explaining the increase in carbon stock. However, there was no association of the other explanatory variables with the recovery of carbon stock from living woody biomass in the Jhum fallows of Chandel.

Table 5: Summary of LMEM across site carbon stock with environmental variables using SPSS

| UKHRUL | | | | | | | | | |
| --- | --- | --- | --- | --- | --- | --- | --- | --- | --- |
| Parameter | Explanatory variables | | | | Log Likelihood | AIC | AICc | ∆ AICc | Weight |
|  | Fallow age | Aspect | Slope | Elevation |  |  |  |  |  |
| log(TLWBC) | x |  |  |  | **28.63** | **40.63** | **42.22** | **0.00** | **0.78** |
|  | x | x |  |  | 11.06 | 37.06 | 44.97 | 2.75 | 0.20 |
|  | x | x | x |  | 0.94 | 34.94 | 49.51 | 7.29 | 0.02 |
|  | x | x | x | x | -4.83 | 37.17 | 61.48 | 19.26 | 0.00 |
| CHANDEL | | | | | | | | | |
| Parameter | Explanatory variables | | | | Log Likelihood | AIC | AICc | ∆ AICc | Weight |
|  | Fallow age | Aspect | Slope | Elevation |  |  |  |  |  |
| log(TLWBC) | x |  |  |  | **-16.54** | **-4.54** | **-2.96** | **0.00** | **1.00** |
|  | x | x |  |  | -23.41 | 2.59 | 10.50 | 13.46 | 0.00 |
|  | x | x | x |  | -27.35 | 6.65 | 21.22 | 24.18 | 0.00 |
|  | x | x | x | x | -52.25 | -10.25 | 14.07 | 17.03 | 0.00 |

**Discussion**

**Characteristics of secondary successive communities on Jhum fallows:**

The structure of secondary communities after the abandonment of Jhum fields generally becomes more complex as a result of species recovery. The findings of this study are in agreement with earlier studies which state that there is an increase in the number of trees with increase in fallow age [30]. The basal area is also considered as an essential predictor of ecological succession, and as reported from similar aged fallows in tropical moist forest, the present study also showed a positive association between basal area and fallow age [31-32]. The values of the Shannon-Wiener diversity index (H') and the Margalef species richness index (D_mg_) along an increasing fallow gradient indicates highly significant diversity and species richness in the successive communities. However, the values of the Simpson’s dominance index (C) and the Pielou’s evenness index (J) were not illustrated as the change was not significant.

**Species composition along an increasing fallow gradient:**

All species response individually to the process of succession, but the similarity of species among fallows is reported by many researchers. The species similarity between 5 year and 10 year fallow in Ukhrul, and 15 year and 20 year fallow in Chandel suggests that the species compositions of fallows having similar age are more identical than those with more differing ages [33-34]. The fallows were predominantly composed of fast-growing pioneer and shade-tolerant tree species. Both pioneer and forest tree species got established in the young fallows and dominated at different stages of succession depending on their life span and growth rate [35].

**Relationship between species composition and topographical parameters:**

In a hilly region, elevation, slope and aspect are considered as key topographical factors that strongly affect the species composition, structure and distribution patterns of vegetation [36]. Elevation affects the micro-climate in several ways and thus results in the variation of communities [37-38]. In the present study, Chandel situated in lower elevation displays higher species richness and stem density than the Ukhrul site which concurs with the results of other workers [39-40]. In the northern hemisphere, southern slopes are directly exposed to radiation while northern exposures are cold and humid which could sustain more tree species and stem density [41-42]. With majority of the sampling sites facing north, our result showed significant relation between species richness and number of individuals along an increasing fallow gradient, identical to the findings by others [43-44].

**Recovery of Jhum fallows:**

The percent recovery of tree species from 5 years fallow to 20 years fallow was more in Ukhrul than in Chandel because the forest recovery increased with increasing elevation [45]. High altitude species have great potential to adapt to diverse micro-climatic situation as high elevation forests are generally open forests and therefore have more ability to recruit in the deforested areas [46]. In our study, five species in Ukhrul and eight species in Chandel were associated with the youngest fallow in the stage of seedlings and saplings, and survived as mature trees in the oldest fallow. Amongst all tree species, *Elaeocarpus* *floribundus* Blume and *Castanopsis* *hystrix* Hook. f. & Thomson ex A. DC. were independent of any environmental variables, and this suggests that they may be capable of effective restoration of degraded forest areas. These tree species can be considered as generalist species as they rapidly establish after abandonment and survive in dense forest. Since both the species occur naturally in wild, they can be utilized for active restoration to accelerate the recovery process of fallows.

**Living woody biomass carbon recovery in Jhum fallows:**

Our result showed a positive relationship between the fallow period and the total living woody biomass carbon which is in accord with other works related to recovery following shifting cultivation in the tropical forests and in neotropical secondary forests [47-49] as well. In our study we used locally developed and calibrated allometric models to reduce errors in carbon accounting [50]. From the many allometric models developed for the tropical region, we opted for the equation given by IPCC (2003) for those species without volume equation. The reason for this option is that the value of AGB obtained from IPCC (2003) equation gave the highest accuracy with reference to species specific equation with diminutive over-estimation (Appendix 4).

**Implications for management of Jhum fallows:**

Over the years, it is accepted that regrowth forests in the tropics provide similar ecosystem goods and services as old-growth forests [51]. This region however has been a huge contributor of greenhouse gas emission due to deforestation and adapting proper land-use system will help recover biomass carbon stock in the forests [52]. Tree plantation may be adapted to accelerate the steady process of natural succession in the fallows thereby restoring the species richness and enhancing the carbon sinks. And as per our findings, *Elaeocarpus* *floribundus* Blume and *Castanopsis* *hystrix* Hook. f. & Thomson ex A. DC. which occurs naturally in wild can be utilized as potential species for active restoration to accelerate the recovery process of fallows.

**Conclusion**

With the decrease in the pristine forests, secondary forests are of greater importance and value in the conservation and restoration of tropical biodiversity. Species richness and basal area recovered relatively with time since abandonment in our study, while the species composition recovery showed resemblance between similar-aged fallows. Topographical factors namely elevation, slope and aspect influenced the high species richness and stem density of fallows in Chandel than in Ukhrul. Regeneration fallow stands are considered to mitigate forest degradation and contribute to global carbon sequestration. We found that the fallow age plays a vital role in recovering aboveground biomass from living woody species followed by the aspect of the site. In this study, we identified two species which can be used in the active restoration of Jhum fallows and which colonize early fallows and appear naturally in the forests. The seedlings of these tree species can be collected from the surrounding forests or germinated and planted in jhum fallows to accelerate natural succession. Tropical Amazonia had observed 90% survival in the transplantation of seedlings into fallows with partially established vegetation [53]. This would facilitate quicker rehabilitation and reinstate the soil nutrients making the soil reusable in a short term, such that the expansion of slashing and burning of primary forests for cultivation would be reduced. A gradual increase in the diversity of woody species along the fallow age evinces the potential of longer fallow forests to enhance carbon stock. Further, the results would help in formulating appropriate policy interventions on conservation strategies and developing climate change mitigation policies for efficient preservation of forest carbon stocks.

**Acknowledgement**

The authors thank the Head of Department, Department of Forestry, Mizoram University for providing the necessary equipment for carrying out this work.

**References**

1. Brady NC. Alternatives to slash-and-burn: A global imperative. Agric Ecosyst Environ. 1996; 58:3-11.
2. Fearnside PM. Global warming and traditional land-use change: greenhouse gas emissions from biomass burning, decomposition and soils in forest conversion, shifting cultivation and secondary vegetation. Clim Change. 2000; 46:115-158.
3. Kotto-Same J, Woomer PL, Appolinaire M, Louis Z. Carbon dynamics in slash-and-burn agriculture and land use alternatives of the humid forest zone in Cameroon. Agric Ecosyst Environ. 1997; 809: 245-256.
4. Ramakrishnan PS. Shifting agriculture and sustainable development: An interdisciplinary study from North-eastern India. UNESCO-MAP Series, Paris (republished by Oxford University Press, New Delhi, India).
5. Mertz O. The relationship between length of fallow and crop yields in shifting cultivation: a rethinking. Agrofor Syst. 2002; 55: 149-159.
6. Thong P, Pebam R, Sahoo UK. A geospatial approach to understand the dynamics of shifting cultivation in Champhai district of Mizoram, North-East India. J Indian Soc Remote. 2018; 46: 1713-1723.
7. Thong P, Sahoo UK, Pebam R, Thangjam U. Spatial and temporal dynamics of shifting cultivation in Manipur, North-East India based on time-series satellite data. Remote Sens App: Soc Environ. 2019; 14: 126-137.
8. NRSC (National Remote Sensing Centre). Wasteland atlas of India. Indian Space Research Organisation. Hyderabad: India; 2010.
9. FAO (Food and Agriculture Organization of the United Nations). Global forest resources assessment. FAO Forestry Report. Rome: Italy; 2010.
10. Chaturvedi AN. Management of secondary forests. Wasteland News.1992; 7: 40-44.
11. Heinimann A, Mertz O, Frolking S, Christensen AE, Hurni K, Sedano F,et al. A global view of shifting cultivation: recent, current and future extent. PLoS ONE. 2017; 12: 1-21.
12. N'Dja K, Justin K, Guillaume D. Successional patterns of plant species and community diversity in a semi-deciduous tropical forest under shifting cultivation. J Veg Sci. 2008; 19: 809-820.
13. Thong P, Pebam R, Sahoo UK Recovery pattern of vegetation during succession following slash and burn agriculture in Mizoram, North-East India. Journal of Plant Biology and Soil Health. 2016; 3: 1-8.
14. Brown SL, Schrooder P, Kern JS. Spatial distribution of biomass in forests of the eastern USA. Forest Ecol Manag. 1999; 123: 81-90.
15. India State of Forest Report. Forest Survey of India. Dehradun: India; 2017.
16. Mueller-Dombois D, Ellenberg H. Aims and methods of vegetation ecology. New York: USA; 1974.
17. Whitford PB. Distribution of woodland plants in relation to succession and clonal growth. Ecology. 1948; 30: 199-208.
18. Shannon CE, Weaver W. The mathematical theory of communication. Urbana: USA; 1949.
19. Simpson EH. Measurement of diversity. Nature. 1949; 163: 688.
20. Pielou EC. Species diversity and pattern diversity in the study of ecological succession. J Theor Biol. 1966; 10: 370-383.
21. Margalef R. Perspectives in ecological theory. Chicago: USA; 1968.
22. Whittaker RH. Vegetation of the Siskiyou mountains, Oregon and California. Ecol Monogr. 1960; 30: 279-338.
23. Jaccard P. Comparative study of floral distribution in a portion of Alps and Jura. Bulletin de la Soci'et'e Vaudoise des Sciences Naturelles. 1901; 37: 547-579. French.
24. FSI (Forest Survey of India). Volume equations for forests of India, Nepal and Bhutan, Dehradun: India; 1996.
25. Zanne AE, Lopez-Gonzalez G, Coomes DS, Ilic J, Jansen S, Lewis SL, et al. Global wood density database; 2009.
26. IPCC (Intergovernmental Panel on Climate Change). Good practice guidance for Land Use, Land-Use Change and Forestry. IPCC National Greenhouse Gas Inventories Programme. Hayama: Japan; 2003.
27. Chaturvedi RK, Raghubanshi AS, Singh JS. Biomass estimation of dry tropical woody species at juvenile stage. Sci World J. 2012: 1-5.
28. Aalde H, Gonzalez P, Gytarsky M, Krug T, Kurz WA, Ogle S, Somogyi Z. Agriculture, Foresry and Other Land Use. In: Eggleston HS, Buendia L, Miwa K, Ngara T, Tanabe K, editors. IPCC Guidelines for National Greenhouse Gas Inventories. Iges, Hayama: Japan; 2006.
29. Mokany K, Raison RJ, Prokushkin AS. Critical analysis of root: shoot ratios in terrestrial biomes. Glob Change Biol. 2006; 12: 84-96.
30. Klanderud L, Mbolatiana HZH, Vololomboahangy MN, Radimbison MA, Roger E, Totland Ø, et al. Recovery of plant species richness and composition after slash-and-burn agriculture in tropical rainforest in Madagascar. Biodivers Conserv. 2010; 19: 187-204.
31. Lohbeck M, Poorter L, Paz H, Pla L, Breugel M.van, Martinez-Ramos M, et al. Functional diversity changes during tropical forest succession. Perspect Plant Ecol Evol Syst. 2012; 14: 89-96.
32. Kammesheidt K. The role of tree sprouts in the restoration of stand structure and species diversity in tropical moist forest after slash-and-burn agriculture in Eastern Paraguay. Plant Ecol. 1998; 139: 155-165.
33. Toledo M, Salick J. Secondary succession and indigenous management in semideciduous forest fallows of the Amazon Basin. Biotropica. 2006; 38: 161-170.
34. Aweto AO. Secondary succession and soil fertility restoration in south-western Nigeria I, Succession. J Ecol. 1981; 69: 601-607.
35. Pena-Claros M. Change in forest structure and species composition during secondary forest succession in the Bolivian Amazon. Biotropica. 2003; 35: 450-461.
36. Titshall LW, O’Connor TG, Morris CD. Effect of long-term exclusion of fire and herbivory on the soils and vegetation of sour grassland. Afr J Range For Sci. 2000; 17: 70-80.
37. Kikvidze Z, Khetsuriani L, Kikodze D, Callaway RM. Seasonal shifts in competition and facilitation in subalpine plant communities of the central Caucasus. J Veg Sci. 2006; 17: 77-82.
38. Vittoz P, Bayfield N, Brooker R, Elston DA, Duff EI, Theurillat JP, et al. Reproducibility of species lists, visual cover estimates and frequency methods for recording high-mountain vegetation. J Veg Sci. 2010; 21: 1035-1047.
39. Kӧrner C. Why are there global gradient in species richness? Mountains might hold the answer. Trends Ecol Evol. 2000; 15: 513-514.
40. Odland A, Birks HJB. The altitudinal gradient of vascular plant richness in Aurland, Western Norway. Ecography. 1999; 22: 548-566.
41. Geiger R, Aron RH, Todhunter P. The climate near the ground. 6th ed. Maryland: USA; 2003.
42. Maren IE, Karki S, Prajapati C, Yadav RK, Shretha BB. Facing north or south: Does slope aspect impact forest stand characteristics and soil properties in a semiarid trans-Himalayan Valley?. J Arid Environ. 2015; 121: 112-123.
43. Lovett JC, Marshall AR, Carr J. Changes in tropical forest vegetation along an altitudinal gradient in the Udzungwa Mountains National Park, Tanzania. Afr J Ecol. 2006; 44: 478-490.
44. Zhang JT, Zhang F. Diversity and composition of plant functional groups in mountain forests of the Lishan Nature Reserve, North China. Botanical Studies. 2007; 48: 339-348.
45. Lippok D, Beck SG, Renison D, Gallegos SC, Saavedra FV, Hensen I, et al. Forest recovery of areas deforested by fire increases with elevation in the tropical Andes. Forest Ecol Manag. 2013; 295: 69-76.
46. Homeier J, Breckle SW, Günter S, Rollenbeck RT, Leuschner C. Tree diversity, forest structure and productivity along altitudinal and topographical gradients in a species-rich Ecuadorian montane rain forest. Biotropica. 2010; 42: 140-148.
47. Pelletier J, Codjia C, Potvin C. Traditional shifting agriculture: Tracking forest carbon stock and biodiversity through time in Western Panama. Glob Change Biol. 2012; 18: 3581-3595.
48. Chan N, Takeda S, Suzuki R, Yamamoto S. Assessment of biomass recovery and soil carbon storage of fallow forests after swidden cultivation in the Bago Mountains, Myanmar. New For. 2016; 47: 565-585.
49. Poorter L, Bongers F, Aide TM, Zambrano AMA, Balvanera P, Becknell, et al. Biomass resilience of Neotropical secondary forests. Nature. 2016; 530: 211-214.
50. Chave J, Andalo C, Brown S, Cairns MA, Chambers JQ, Eamus D, et al. Tree allometry and improved estimation of carbon stocks and balance in tropical forests. Oecologia. 2005; 145: 87-99.
51. Bonner MTL, Schmidt S, Shoo LP. A meta-analytical global comparison of aboveground biomass accumulation between tropical secondary forests and monoculture plantations. Forest Ecol Manag. 2013; 291: 73-86.
52. Budiharta S, Meijaard E, Erskine PD, Rondinini C, Pacifici M, Wilson KA. (2014). Restoring degraded tropical forests for carbon and biodiversity. Environ Res Lett. 2014; 9: 114020.
53. Uhl C. Factors controlling succession following slash-and-burn agriculture in Amazonia. J Ecol. 1987; 75: 337-407.
