# Supplementary material for: Pattern of forest recovery and carbon stock following shifting cultivation in Manipur, North-East India
Source: PLoS One. 2020 Oct 8;15(10):e0239906. doi: 10.1371/journal.pone.0239906 (PMC7544089; doi:10.1371/journal.pone.0239906)
Supplement: Attachment — Submitted filename: Response_to_Reviewers.docx [file pone.0239906.s007.docx]

**Response to Reviewer’s comments**

**Respected Gerhard Hartwig Buck-Sorlin**, Ph.D.
Academic Editor, PLOS ONE

Thank you very much for giving us an opportunity to re-submit our manuscript (PONE-D-19-29248) entitled “PATTERN OF FOREST RECOVERY AND CARBON STOCK FOLLOWING SHIFTING CULTIVATION IN MANIPUR, NORTH-EAST INDIA” in revised form.

I wish to mention that we have thoroughly revised the manuscript by considering all the comments of the reviewer. We immensely thank the anonymous reviewer for his critical comments and inputs which has helped us in improving the manuscript.

The point-wise response to the reviewer’s comment is as follows.

**Comment [A1]:** Similar to Mukul et al 2016 Scientific Reports | 6:22483 | DOI: 10.1038/srep22483

**Response**: The sentence has been reframed as ‘abandoned fallows are known to have high potential for carbon capture’.

**Comment [A2]:** See Kenzo et al 2009 Journal of Tropical Ecology (2009) 25:371-386

**Response:** The sentence has been reframed as ‘have the ability to assimilate and store carbon’.

**Comment [A3]:** Typo (Jhum)

**Response:** The typographical error has been corrected to ‘Jhum’.

**Comment [A4]:** Fast growing group of species.

**Response**: The sentence has been reframed.

**Comment** **[A5]:** From where did this assumption came from? See Poorter et al 2016 “Here we analyse aboveground biomass recovery during secondary succession in 45 forest sites and about 1,500 forest plots covering the major environmental gradients in the Neotropics. The studied secondary forests are highly productive and resilient. Aboveground biomass recovery after 20 years was on average 122megagrams per hectare (Mgha−1 ), corresponding to a net carbon uptake of 3.05 MgCha−1 yr−1 , 11 times the uptake rate of old-growth forests. Aboveground biomass stocks took a median time of 66 years to recover to 90% of old-growth values. Aboveground biomass recovery after 20 years varied 11.3-fold (from 20 to 225Mg ha−1 ) across sites, and this recovery increased with water availability (higher local rainfall and lower climatic water deficit).”NATURE | VOL 530 | 11 February 2016doi:10.1038/nature16512

**Response:** The highlighted sentenced has been removed from the manuscript.

**Comment [A6]:** Typo

**Response:** The typographical error has been corrected to ‘Jhum’.

**Comment [A7]:** 2 districts (four fallow aged plots x three replicates total of 24 sites

**Response:** We identified a total of 12 sites (four fallow aged plots x three replicates) in each district which sums up to 24 sites.

**Comment [A8]:** Brown equation was developed using 226 trees between 5-148 cm in dbh.

**Response:** Tree species has been assessed at different growth stages namely mature tree, pole, sapling and seedling, and therefore, different dbh classification has been adopted for diversity assessment and carbon stock assessment.

**Comment [A9]:** Uncertainty for each equation, and uncertainty assumed for wood density?

“When allometric equations developed from a biome-wide database, such as those in Annex 4A.2, Tables 4.A.1 and 4.A.2, are used, it is good practice to verify the equation by destructively harvesting, within the project area but outside the sample plots, a few trees of different sizes and estimate their biomass and then compare against a selected equation. If the biomass estimated from the harvested trees is within about +/- 10% of that predicted by the equation, then it can be assumed that the selected equation is suitable for the project. If this is not the case, it is recommended to develop local allometric equations for the project use.”

Equation Forest type a R2 /sample size DBH range (cm) Y = exp[–2.289 + 2.649 • ln (DBH) – 0.021 • (ln(DBH))2 ] Tropical moist hardwoods 0.98/226 5 - 148

**Response:** We used local allometric equations for the region and obtained the wood density value from Global wood density database. So the uncertainties for both were not assessed.

**Comment [A10]:** Tropical moist hardwood equation for dry mass (Table 4.A.1) page

**Response:** This equation has been removed and we opted for the best fit model in the tropics.

**Comment [A11]:** For saplings See Ribeiro et al 2014 <http://www.jircas.affrc.go.jp/>

**Response:** The equation developed for saplings by Ribeiro et al. (2014) overestimates the biomass. Therefore, we chose equation given by Ali et al. (2015).

**Comment [A12]:** Standard error of estimative 0.196 (saplings below 10 cm in dbh and taller than 30 cm) derived from tropical dry wood species (different root:shoot allocation?)

**Response:** This equation has been rejected.

**Comment [A13]:** IPCC, 2006 vol 4 table 4.3 recommends 49% for trees above 10cm in DBH. The default value of 47% is recommended for total tree biomass. The recommendation derives from Hughes, R.F., Kauffman, J.B. and Jaramillo, V.J. (1999). Biomass, carbon, and nutrient dynamics of secondary forests in a humid tropical region of México. Ecology 80: 1892-1907.

**Response:** The above ground carbon stock for woody trees with dbh˂10 cm, and ≥10 cm were calculated as 46% and 49% of the ABG respectively.

**Comment [A14]:** Dry biomass equation, you need to know the moisture content to talk about the living biomass for the species without volume equations evaluable.

**Response:** Since non-destructive method was adopted this study, we referred to the root shoot ratio for biomass estimation below ground.

**Comment [A15]:** Reported Standard error of 0.036 (Table 2 – Global Change Biology, 12, 84–96)

**Response:** The root shoot ratio of 0.205 is the most frequently used value for the tropical/ subtropical moist forest regardless of the SE value.

**Comment [A16]:** Include the uncertainty for each compartment

**Response**: The uncertainty % for AGBC and BGBC in all the fallow stands has been mentioned in the result and also incorporated in the Supporting information.

**Comment [A17]:** Time limits the appearance of new species in fallow ages

**Response:** The sentence has been reconstructed as ‘The rarefaction curves of species accumulation indicated that the tree species richness curves in all the fallow ages reached a plateau with increasing fallow age which validates that time duration limits the appearance of new species in fallow ages’.

**Comment [A18]:** Altitude995-1537m a.s.l smaller range than Chandel. Is there any threshold that limits species composition in higher altitudes?

**Response:** There is no significant correlation between elevation and fallow replicates of species composition in Ukhrul and Chandel. Therefore, this question is not applicable.

**Comment [A19]:** 398-1247 m a.s.l

**Response:** There is no significant correlation between elevation and fallow replicates of species composition in Ukhrul and Chandel. Therefore, this question is not applicable

**Comment [A20]:** Is there any similarity among the environmental factors for this sites?

**Response:** Environmental factors namely slope and aspect were found to be similar between 5 and 10 years fallow in Ukhrul as well as between 15 and 20 years fallow in Chandel. This explains for the high species similarity between the fallows (see S1 File).

**Comment [A21]:** The fact that the index incorporates both components of biodiversity can be seen as both a strength and a weakness. It is a strength because it provides a simple, synthetic summary, but it is a weakness because it makes it difficult to compare communities that differ greatly in richness. Since evenness and dominance are simply two sides of the same coin, their measures are complimentary. Simpsonʼs index is based on the probability of any two individuals drawn at random from an infinitely large community belonging to the same species.

See Morris et al 2014 Ecology and Evolution 2014; 4(18): 3514– 3524Choosing and using diversity indices: insights for ecological applications from the German Biodiversity Exploratories.

“Magurran and Dornelas (2010) argue against using compound indices when the objective is to detect effects of external factors on diversity, such as when assessing anthropogenic impacts on the environment. There is some empirical evidence that simple indices are indeed more effective in these cases”

**Response:** Since evenness and dominance are simply two sides of the same coin, the evenness index has been removed from the table to simplify the result interpretation.

**Comment [A22]:** “evenness represents the degree to which individuals are split among species with low values indicating that one or a few species dominate, and high values indicating that relatively equal numbers of individuals belong to each species. Evenness is not calculated independently, but rather is derived from compound diversity measures such as H’, D1, and D2, as they inherently contain richness and evenness components. However, evenness as calculated from H’ (J’) is of only limited use predictively because it mathematically correlates with H’ (DeBenedictis 1973). E, calculated from D2 (Table 1), is mathematically independent of D1 (Smith and Wilson 1996) and therefore a more useful measure of evenness in many contexts”

Morris et al 2014 Ecology and Evolution 2014; 4(18): 3514– 3524Choosing and using diversity indices: insights for ecological applications from the German Biodiversity Exploratories

“The greater ability of measures derived from Hill’s N2 (D1, D2) to discriminate

**Response:** Since evenness and dominance are simply two sides of the same coin, the evenness index has been removed from the table to simplify the result interpretation

**Comment [A23]:** E.g. **the Simpson's Dominance Index is telling you that this community (dominated by one specie, but containing even amounts of 9 other species), can be similar to a community with 3 dominant species and not much else. Does this prove anything? Not really -- just because the math works out does not mean that nature has to operate that way.**

**Response:** Data mining to identify an index providing strong significant effects is avoided and the results are presented in the simplest manner.

**Comment [A24]:** Could the authors provide a distribution of species in undisturbed sites along the slope aspect and altitude gradients?

**Response:** The study sites ie the age of fallow stands were traced using satellite data. By performing post-classification comparison, the age of the fallow stand was ascertained. And it would be time consuming effort to trace back to a primary forest/undisturbed sites. Therefore, the findings of Waikom et al. (2017) on sacred groves of Manipur was used as reference for carbon stock recovery.

**Comment [A25]:** We identified a total of 12 sites (four fallow aged plots x three replicates) in each district. How was the slope aspect and elevation for the three replicates? And how that distribution influences the results? It is difficult to check from Fig 1.

**Response:** The detailed descriptions of the sites are presented in table 1. The influences of site distribution are explained in the manuscript (see S1).

**Comment [A26]:** How were the differences for the replicates?

**Response:** It was observed that in Ukhrul, significant difference (*F*=16.71, *P*<0.001 and *F*=8.707, *P*<0.005) was observed in the 15 years fallow replicates of species richness and stem density as well as in the 10 years fallow replicates of Stem density (*F*=4.244, *P*<0.05). On the other hand, Chandel species richness was found to be significant among the replicates of 15 years fallow (*F*=3.912, *P*<0.05) and none of the replicates were significant for stem density.

**Comment [A27]:** Jhum

**Response:** The typographical error has been corrected to ‘Jhum’

**Comment [A28]:** Generalists species

**Response:** The sentence has been reframed as ‘The CCA triplots further showed that these species (ElaeFlor and CastHyst) were generalist species, that is, they appear opposite to the direction of increasing value of the environmental variables and its relation with the tree species’.

**Comment [A29]:** Can the authors provide the number of species that had biomass estimated from Brown 1997 equation? What was the DBH range?

**Response:** The number of species that had biomass estimated from Nath et al. (2019) and FSI volume equation has been mentioned. Species with dbh<5 cm were considered juvenile while the biomass for trees with dbh>5 cm were calculated using either Nath et al. (2019) or FSI volume equation.

**Comment [A30]:** What’s the uncertainty measure for this estimates?

**Response:** The uncertainty % of TLWBC decreased from 84% to 9% in Ukhrul and 60% to 12% in Chandel with increasing fallow age

**Comment [A31]:** That can be explained by the range of altitude for each Jhum fallow, the 5 year sites for UKHRUL 995-1285. 10 year 1179 – 1537. 15 year 1216 – 1404. 20 year 1225 – 1491. That bring my attention for human activity in lower altitudes during last decades. For Chandel site this trend didn’t occur. Extracted from the Excel file in supplementary material.

**Response:** Although slope and elevation displayed a positive correlation with the carbon stock in Ukhrul and Chandel respectively, it did not influence the recovery of carbon stock in the Jhum fallows.

**Comment [A32]:** The authors bring no information related to undisturbed forests. It’s difficult to talk about secondary successive communities without “primary communities” or old-growth forests.

**Response:** The study sites ie the age of fallow stands were traced using satellite data. By performing post-classification comparison, the age of the fallow stand was ascertained. And it would be time consuming effort to trace back to a primary forest/undisturbed sites. Therefore, the findings of Waikom et al. (2017) on sacred groves of Manipur was used as reference for carbon stock recovery.

**Comment [A33]:** What level of uncertainty for the use of wood density? Values for standard error of estimates for the different models/equations used were not clear.

**Response:** These sentences have been removed from the manuscript.

**Comment [A34]:** IPCC suggests the use of Brown 1997 equation when no data availability for the region 2000-4000 mm annual precipitation. IPCC do not prescribe anything.

**Response:** These sentences have been removed from the manuscript.

**Comment [A35]:** Not clear.

**Response:** These sentences have been removed from the manuscript.

**Comment [A36]:** Values for old growth forests not presented

**Response:** Biomass carbon stock from old growth forest/undisturbed forest was used as a control to calculate per cent recovery of the jhum fallows.

**Comment [A37]:** Replications were not represented by all topographical factors

**Response:** Topographical parameters of the replicates are presented in Table 1.

**Comment [A38]:** Along the main text, figures etc check all the Jhum along the manuscript

**Response:** All the typographical error in the spelling of Jhum have been corrected.

**Comment [A39]:** Typo

**Response:** Fernside has been corrected as ‘Fearnside’

**Comment [A40]: Typo**

**Response:** coversion has been corrected as ‘conversion’

I hope, with the above changes, the manuscript may now be acceptable for publication in PLos One.

However, we will still be ready to do any further corrections, if desired.

With kind regards,

Sincerely,

Professor U.K.Sahoo
