# Supplementary material for: Pattern of forest recovery and carbon stock following shifting cultivation in Manipur, North-East India
Source: PLoS One. 2020 Oct 8;15(10):e0239906. doi: 10.1371/journal.pone.0239906 (PMC7544089; doi:10.1371/journal.pone.0239906)
Supplement: Attachment — Submitted filename: Response to Reviewers.docx [file pone.0239906.s008.docx]

**Response to Reviewer’s comments**

**Respected Gerhard Hartwig Buck-Sorlin**, Ph.D.
Academic Editor, PLOS ONE

Thank you very much for giving us an opportunity to re-revise our manuscript (PONE-D-19-29248) entitled “PATTERN OF FOREST RECOVERY AND CARBON STOCK FOLLOWING SHIFTING CULTIVATION IN MANIPUR, NORTH-EAST INDIA” and considering it for minor revision.

I wish to mention that we have thoroughly gone through the comments, and have revised the manuscript accordingly. We immensely thank the anonymous reviewer for his critical comments and inputs which has further improved the revised manuscript.

The point-wise response to the reviewer’s comment is as follows.

**Reviewer #1:** Most comments were proper addressed and the manuscript have improved, but there is a main issue related to the interpretation of biomass and carbon stock values uncertainty and the use of Waikhom et al 2017 data for comparison of carbon stock recovery estimation. A central point after the selection of a allometric equation to apply to your data is understand if this equation gives the fresh biomass estimates or the dry biomass estimates. If the model gave you fresh biomass estimates you should first remove the water content (multipling the estimated value by 1 less water content percentage value) and then convert it to carbon stocks. Waikhom et al 2017 using Chambers et al 2001 equation found the aboveground biomass, carbon stock of the sacred grove ranged from 962.94 to 1130.79 Mg ha-1 and 481.47 to 565.40 Mg ha-1 C. Then Waikhon et al 2017 calculated the aboveground carbon stock by assuming that carbon content is 50% of the total aboveground biomass. And thats why the values reported are so high, e.g for Central Amazon old growth forests, above ground fresh biomass can assume values around 600 Mg.ha-1 and water content around 40.8%, so dry biomass will be the result of 600*(1-0.408)= 355,2 Mg.ha-1 this value assuming carbon content is 50% will rang around 177.6 Mg. ha-1 C.
Chambers et al assumed 38% of water content.
Lines 162-163. The findings of Waikhom et al. [34] on sacred groves of Manipur were used as comparison data for carbon stock recovery estimation.
When i first read tought tha Waikhom et al 2017 developed some allometric model, but instead they use a Central Amazon model developed by Chambers et al 2001.
Jeffrey Q. Chambers worked with 315 trees harvest by the group of Niro Higuchi, i strongly recommend the read of Higuchi N, Suwa R, Higuchi FG, Lima AJN, Santos J dos, Noguchi H, et al. Overview of Forest Carbon Stocks Study in Amazonas State, Brazil. Interactions Between Biosphere, Atmosphere and Human Land Use in the Amazon Basin. Nagy L., Forsberg B., Artaxo P.(eds); 2016. pp. 171–187. doi:10.1007/978-3-662-49902-3
ABSTRACT
"Tropical forests, specially the Amazon, are important ecosystems in research on the effects of climate change on biological systems and their functioning. The main relationship between the forest and the climate is the carbon fluxes and stock. Although carbon stock estimates of tropical forests are an issue and still raise many uncertainties. This study aims to present the main literature references and the huge effort to sample the forests of Central Amazon. Many attempts to estimate the carbon stock of the Amazon rainforest resulted in high uncertainties due to sampling and allometric constrains. After developing an allometric equation, based on destructive method and adapted to each different site based on dominant height, sampling over 1800 plots in primary and selectively logged non-flooded forests, across the State of Amazonas, the estimated average of total (tree’s above- and below-ground) carbon stock is 159.8 ± 9.2 Mg C ha−1."

**Response:** The uncertainty of TLWBC was evaluated as per Higuchi et al. 2016. In the present study, the biomass was estimated based on species specific volume equation as well as region specific allometric model. Conversion from tree volume to carbon content results in larger uncertainties as compared to the use of allometric models [Njana et al. 2017]. This explains the high uncertainty percentage in the present study.

As for the use of Waikhom et al 2017 data for comparison of carbon stock recovery estimation, they estimated the biomass using regression equation developed by Chambers et al. which had 38% moisture content. Therefore, biomass estimated from the sacred grove was converted to dry biomass by removing the water content and then the carbon stock was determined. This was done to assess the recovery per cent of our estimated dry biomass carbon.

I hope, with the above changes, the manuscript may now be accepted for publication in PLos One.

Thanking you once again and with kind regards,

Sincerely,

Professor U.K.Sahoo
