# Supplementary material for: Pattern of forest recovery and carbon stock following shifting cultivation in Manipur, North-East India
Source: PLoS One. 2020 Oct 8;15(10):e0239906. doi: 10.1371/journal.pone.0239906 (PMC7544089; doi:10.1371/journal.pone.0239906)
Supplement: Peer review history

# Peer review: Decision Letter 0

**DOI:** 10.1371/journal.pone.0239906.r001

**Authors:** Gerhard Hartwig Buck-Sorlin

29 Apr 2020

PONE-D-19-29248

PATTERN OF FOREST RECOVERY AND CARBON STOCK FOLLOWING SHIFTING CULTIVATION IN MANIPUR, NORTH-EAST INDIA

PLOS ONE

Dear Prof Sahoo,

Thank you for submitting your manuscript to PLOS ONE. After careful consideration, we feel that it has merit but does not fully meet PLOS ONE’s publication criteria as it currently stands. Therefore, we invite you to submit a revised version of the manuscript that addresses the points raised during the review process.

First of all, I would like to apologize for the the very long time it has taken to come back to you with a decision. However, it has been very difficult to recruit reviewers for your manuscript. I have therefore decided to base my decision on the recommendation of only one reviewer (see below): The manuscript is at the moment not acceptable for publication in PLOS ONE. I recommend that you subject your manuscript to a very thorough revision along the lines suggested by the reviewer before resubmitting it. You should in particular rethink your use of allometric equations to estimate biomass, and enrich the discussion with more literature (see below). Please accompany your revised manuscript with a detailed letter in which you answer to the reviewer how you revised your manuscript point by point.

We would appreciate receiving your revised manuscript by Jun 13 2020 11:59PM. When you are ready to submit your revision, log on to https://www.editorialmanager.com/pone/ and select the 'Submissions Needing Revision' folder to locate your manuscript file.

If you would like to make changes to your financial disclosure, please include your updated statement in your cover letter.

To enhance the reproducibility of your results, we recommend that if applicable you deposit your laboratory protocols in protocols.io, where a protocol can be assigned its own identifier (DOI) such that it can be cited independently in the future. For instructions see: http://journals.plos.org/plosone/s/submission-guidelines#loc-laboratory-protocols

Please include the following items when submitting your revised manuscript:

- A rebuttal letter that responds to each point raised by the academic editor and reviewer(s). This letter should be uploaded as separate file and labeled 'Response to Reviewers'.
- A marked-up copy of your manuscript that highlights changes made to the original version. This file should be uploaded as separate file and labeled 'Revised Manuscript with Track Changes'.
- An unmarked version of your revised paper without tracked changes. This file should be uploaded as separate file and labeled 'Manuscript'.

Please note while forming your response, if your article is accepted, you may have the opportunity to make the peer review history publicly available. The record will include editor decision letters (with reviews) and your responses to reviewer comments. If eligible, we will contact you to opt in or out.

We look forward to receiving your revised manuscript.

Kind regards,

Gerhard Hartwig Buck-Sorlin, Ph.D.

Academic Editor

PLOS ONE

Journal Requirements:

When submitting your revision, we need you to address these additional requirements.

1. Please ensure that your manuscript meets PLOS ONE's style requirements, including those for file naming. The PLOS ONE style templates can be found at https://journals.plos.org/plosone/s/file?id=wjVg/PLOSOne_formatting_sample_main_body.pdf and https://journals.plos.org/plosone/s/file?id=ba62/PLOSOne_formatting_sample_title_authors_affiliations.pdf

2. We note that Figure 1 in your submission contain [map/satellite] images which may be copyrighted. All PLOS content is published under the Creative Commons Attribution License (CC BY 4.0), which means that the manuscript, images, and Supporting Information files will be freely available online, and any third party is permitted to access, download, copy, distribute, and use these materials in any way, even commercially, with proper attribution. For these reasons, we cannot publish previously copyrighted maps or satellite images created using proprietary data, such as Google software (Google Maps, Street View, and Earth). For more information, see our copyright guidelines: http://journals.plos.org/plosone/s/licenses-and-copyright.

We require you to either (1) present written permission from the copyright holder to publish these figures specifically under the CC BY 4.0 license, or (2) remove the figures from your submission:

1.    You may seek permission from the original copyright holder of Figure 1 to publish the content specifically under the CC BY 4.0 license.

We recommend that you contact the original copyright holder with the Content Permission Form (http://journals.plos.org/plosone/s/file?id=7c09/content-permission-form.pdf) and the following text:

“I request permission for the open-access journal PLOS ONE to publish XXX under the Creative Commons Attribution License (CCAL) CC BY 4.0 (http://creativecommons.org/licenses/by/4.0/). Please be aware that this license allows unrestricted use and distribution, even commercially, by third parties. Please reply and provide explicit written permission to publish XXX under a CC BY license and complete the attached form.”

Please upload the completed Content Permission Form or other proof of granted permissions as an "Other" file with your submission.

In the figure caption of the copyrighted figure, please include the following text: “Reprinted from [ref] under a CC BY license, with permission from [name of publisher], original copyright [original copyright year].”

2.    If you are unable to obtain permission from the original copyright holder to publish these figures under the CC BY 4.0 license or if the copyright holder’s requirements are incompatible with the CC BY 4.0 license, please either i) remove the figure or ii) supply a replacement figure that complies with the CC BY 4.0 license. Please check copyright information on all replacement figures and update the figure caption with source information. If applicable, please specify in the figure caption text when a figure is similar but not identical to the original image and is therefore for illustrative purposes only.

The following resources for replacing copyrighted map figures may be helpful:

USGS National Map Viewer (public domain): http://viewer.nationalmap.gov/viewer/

The Gateway to Astronaut Photography of Earth (public domain): http://eol.jsc.nasa.gov/sseop/clickmap/

Maps at the CIA (public domain): https://www.cia.gov/library/publications/the-world-factbook/index.html and https://www.cia.gov/library/publications/cia-maps-publications/index.html

NASA Earth Observatory (public domain): http://earthobservatory.nasa.gov/

Landsat: http://landsat.visibleearth.nasa.gov/

USGS EROS (Earth Resources Observatory and Science (EROS) Center) (public domain): http://eros.usgs.gov/#

Natural Earth (public domain): http://www.naturalearthdata.com/

3. We note that you have included the phrase “data not shown” in your manuscript. Unfortunately, this does not meet our data sharing requirements. PLOS does not permit references to inaccessible data. We require that authors provide all relevant data within the paper, Supporting Information files, or in an acceptable, public repository. Please add a citation to support this phrase or upload the data that corresponds with these findings to a stable repository (such as Figshare or Dryad) and provide and URLs, DOIs, or accession numbers that may be used to access these data. Or, if the data are not a core part of the research being presented in your study, we ask that you remove the phrase that refers to these data.

4. Please include captions for your Supporting Information files at the end of your manuscript, and update any in-text citations to match accordingly. Please see our Supporting Information guidelines for more information: http://journals.plos.org/plosone/s/supporting-information.

[Note: HTML markup is below. Please do not edit.]

Reviewers' comments:

Reviewer's Responses to Questions

**Comments to the Author**

1. Is the manuscript technically sound, and do the data support the conclusions?

The manuscript must describe a technically sound piece of scientific research with data that supports the conclusions. Experiments must have been conducted rigorously, with appropriate controls, replication, and sample sizes. The conclusions must be drawn appropriately based on the data presented. 

Reviewer #1: No

**********

2. Has the statistical analysis been performed appropriately and rigorously? 

Reviewer #1: No

**********

3. Have the authors made all data underlying the findings in their manuscript fully available?

The PLOS Data policy requires authors to make all data underlying the findings described in their manuscript fully available without restriction, with rare exception (please refer to the Data Availability Statement in the manuscript PDF file). The data should be provided as part of the manuscript or its supporting information, or deposited to a public repository. For example, in addition to summary statistics, the data points behind means, medians and variance measures should be available. If there are restrictions on publicly sharing data—e.g. participant privacy or use of data from a third party—those must be specified.

Reviewer #1: No

**********

4. Is the manuscript presented in an intelligible fashion and written in standard English?

PLOS ONE does not copyedit accepted manuscripts, so the language in submitted articles must be clear, correct, and unambiguous. Any typographical or grammatical errors should be corrected at revision, so please note any specific errors here.

Reviewer #1: Yes

**********

5. Review Comments to the Author

Please use the space provided to explain your answers to the questions above. You may also include additional comments for the author, including concerns about dual publication, research ethics, or publication ethics. (Please upload your review as an attachment if it exceeds 20,000 characters)

Reviewer #1: I have no competing interests.

(1) understand the tree recovery pattern following shifting cultivation( didn't bring any information about the old-growth forests); (2) estimate the living woody biomass and carbon stock under different fallow regimes. The authors did not bring sufficient evidence to support use of allometric equation developed by Sandra Brown, from IPCC, 2003 “When allometric equations developed from a biome-wide database, such as those in Annex 4A.2, Tables 4.A.1 and 4.A.2, are used, it is good practice to verify the equation by destructively harvesting, within the project area but outside the sample plots, a few trees of different sizes and estimate their biomass and then compare against a selected equation. If the biomass estimated from the harvested trees is within about +/- 10% of that predicted by the equation, then it can be assumed that the selected equation is suitable for the project. If this is not the case, it is recommended to develop local allometric equations for the project use.”

The equation is for dry above ground biomass.

Equation Forest type Tropical moist hardwoods Y = exp[–2.289 + 2.649 • ln (DBH) – 0.021 • (ln(DBH))2 ] R2 /sample size 0.98/226 5 - DBH range (cm) 148. It's not clear what are the uncertainties for each volumetric equation, for the use of wood density values. That just an empty estimate of Biomass/Carbon.

There is a lot of typo regarding the word Jhum along the mais text, figures and also in bibliography e.g. Fernside (Fearnside)

"The biomass and carbon stock of 79 living woody tree species were evaluated from species specific allometric models (Appendix 2)" Appendix 2 just bring the 9 figures.

Replications were not represented by all topographical factors.

We identified a total of 12 sites (four fallow aged plots x three replicates) in each district. How was the slope aspect and elevation for the three replicates? And how that distribution influences the results? It is difficult to check from Fig 1.

That can be explained by the range of altitude for each Jhum fallow, the 5 year sites for UKHRUL 995-1285. 10 year 1179 – 1537. 15 year 1216 – 1404. 20 year 1225 – 1491. That bring my attention for human activity in lower altitudes during last decades. For Chandel site this trend didn’t occur. Extracted from the Excel file in supplementary material.

There is a lot of typo regarding the word Jhum along the mais text, figures.

"The biomass and carbon stock of 79 living woody tree species were evaluated from species specific allometric models (Appendix 2)" Appendix 2 just bring the 9 figures.

"The reason for this option is that the value of AGB obtained from IPCC (2003) equation gave the highest accuracy with reference to species specific equation with diminutive over-estimation (Appendix 4)." Didn't find Appendix 4 in any of the available files ( Supplentary file.xlsx; Supporting information.docx and Figures.docx)

Please include the uncertainty in Fig 9.

Similar research was conducted by Mukul et al 2016 Scientific Reports | 6:22483 | DOI: 10.1038/srep22483, and it would enrich the discussion.

**********

6. PLOS authors have the option to publish the peer review history of their article (what does this mean?). If published, this will include your full peer review and any attached files.

If you choose “no”, your identity will remain anonymous but your review may still be made public.

**Do you want your identity to be public for this peer review?** For information about this choice, including consent withdrawal, please see our Privacy Policy.

Reviewer #1: No

[NOTE: If reviewer comments were submitted as an attachment file, they will be attached to this email and accessible via the submission site. Please log into your account, locate the manuscript record, and check for the action link "View Attachments". If this link does not appear, there are no attachment files to be viewed.]

While revising your submission, please upload your figure files to the Preflight Analysis and Conversion Engine (PACE) digital diagnostic tool, https://pacev2.apexcovantage.com/. PACE helps ensure that figures meet PLOS requirements. To use PACE, you must first register as a user. Registration is free. Then, login and navigate to the UPLOAD tab, where you will find detailed instructions on how to use the tool. If you encounter any issues or have any questions when using PACE, please email us at figures@plos.org. Please note that Supporting Information files do not need this step.

Attachment Submitted filename: Review for Manuscript Number PONE-D-19-29248.docx Click here for additional data file.

# Author comment: Author response to Decision Letter 0

**DOI:** 10.1371/journal.pone.0239906.r002

16 Jun 2020

Respected Gerhard Hartwig Buck-Sorlin, Ph.D.

Academic Editor, PLOS ONE

Thank you very much for giving us an opportunity to re-submit our manuscript (PONE-D-19-29248) entitled “PATTERN OF FOREST RECOVERY AND CARBON STOCK FOLLOWING SHIFTING CULTIVATION IN MANIPUR, NORTH-EAST INDIA” in revised form. 

I wish to mention that we have thoroughly revised the manuscript by considering all the comments of the reviewer. We immensely thank the anonymous reviewer for his critical comments and inputs which has helped us in improving the manuscript.

The point-wise response to the reviewer’s comment is as follows.

Comment [A1]: Similar to Mukul et al 2016 Scientific Reports | 6:22483 | DOI: 10.1038/srep22483

Response: The sentence has been reframed as ‘abandoned fallows are known to have high potential for carbon capture’.

Comment [A2]: See Kenzo et al 2009 Journal of Tropical Ecology (2009) 25:371-386

Response: The sentence has been reframed as ‘have the ability to assimilate and store carbon’.

Comment [A3]: Typo (Jhum)

Response: The typographical error has been corrected to ‘Jhum’.

Comment [A4]: Fast growing group of species. 

Response: The sentence has been reframed.

Comment [A5]: From where did this assumption came from? See Poorter et al 2016 “Here we analyse aboveground biomass recovery during secondary succession in 45 forest sites and about 1,500 forest plots covering the major environmental gradients in the Neotropics. The studied secondary forests are highly productive and resilient. Aboveground biomass recovery after 20 years was on average 122megagrams per hectare (Mgha−1 ), corresponding to a net carbon uptake of 3.05 MgCha−1 yr−1 , 11 times the uptake rate of old-growth forests. Aboveground biomass stocks took a median time of 66 years to recover to 90% of old-growth values. Aboveground biomass recovery after 20 years varied 11.3-fold (from 20 to 225Mg ha−1 ) across sites, and this recovery increased with water availability (higher local rainfall and lower climatic water deficit).”NATURE | VOL 530 | 11 February 2016doi:10.1038/nature16512

Response: The highlighted sentenced has been removed from the manuscript.

Comment [A6]: Typo 

Response: The typographical error has been corrected to ‘Jhum’.

Comment [A7]: 2 districts (four fallow aged plots x three replicates total of 24 sites

Response: We identified a total of 12 sites (four fallow aged plots x three replicates) in each district which sums up to 24 sites.

Comment [A8]: Brown equation was developed using 226 trees between 5-148 cm in dbh.

Response: Tree species has been assessed at different growth stages namely mature tree, pole, sapling and seedling, and therefore, different dbh classification has been adopted for diversity assessment and carbon stock assessment.

Comment [A9]: Uncertainty for each equation, and uncertainty assumed for wood density?

“When allometric equations developed from a biome-wide database, such as those in Annex 4A.2, Tables 4.A.1 and 4.A.2, are used, it is good practice to verify the equation by destructively harvesting, within the project area but outside the sample plots, a few trees of different sizes and estimate their biomass and then compare against a selected equation. If the biomass estimated from the harvested trees is within about +/- 10% of that predicted by the equation, then it can be assumed that the selected equation is suitable for the project. If this is not the case, it is recommended to develop local allometric equations for the project use.”

Equation Forest type a R2 /sample size DBH range (cm) Y = exp[–2.289 + 2.649 • ln (DBH) – 0.021 • (ln(DBH))2 ] Tropical moist hardwoods 0.98/226 5 - 148 

Response: We used local allometric equations for the region and obtained the wood density value from Global wood density database. So the uncertainties for both were not assessed.

Comment [A10]: Tropical moist hardwood equation for dry mass (Table 4.A.1) page 

Response: This equation has been removed and we opted for the best fit model in the tropics.

Comment [A11]: For saplings See Ribeiro et al 2014 http://www.jircas.affrc.go.jp/

Response: The equation developed for saplings by Ribeiro et al. (2014) overestimates the biomass. Therefore, we chose equation given by Ali et al. (2015).

Comment [A12]: Standard error of estimative 0.196 (saplings below 10 cm in dbh and taller than 30 cm) derived from tropical dry wood species (different root:shoot allocation?)

Response: This equation has been rejected.

Comment [A13]: IPCC, 2006 vol 4 table 4.3 recommends 49% for trees above 10cm in DBH. The default value of 47% is recommended for total tree biomass. The recommendation derives from Hughes, R.F., Kauffman, J.B. and Jaramillo, V.J. (1999). Biomass, carbon, and nutrient dynamics of secondary forests in a humid tropical region of México. Ecology 80: 1892-1907.

Response: The above ground carbon stock for woody trees with dbh˂10 cm, and ≥10 cm were calculated as 46% and 49% of the ABG respectively.

Comment [A14]: Dry biomass equation, you need to know the moisture content to talk about the living biomass for the species without volume equations evaluable. 

Response: Since non-destructive method was adopted this study, we referred to the root shoot ratio for biomass estimation below ground.

Comment [A15]: Reported Standard error of 0.036 (Table 2 – Global Change Biology, 12, 84–96)

Response: The root shoot ratio of 0.205 is the most frequently used value for the tropical/ subtropical moist forest regardless of the SE value.

Comment [A16]: Include the uncertainty for each compartment

Response: The uncertainty % for AGBC and BGBC in all the fallow stands has been mentioned in the result and also incorporated in the Supporting information.

Comment [A17]: Time limits the appearance of new species in fallow ages

Response: The sentence has been reconstructed as ‘The rarefaction curves of species accumulation indicated that the tree species richness curves in all the fallow ages reached a plateau with increasing fallow age which validates that time duration limits the appearance of new species in fallow ages’.

Comment [A18]: Altitude995-1537m a.s.l smaller range than Chandel. Is there any threshold that limits species composition in higher altitudes? 

Response: There is no significant correlation between elevation and fallow replicates of species composition in Ukhrul and Chandel. Therefore, this question is not applicable.

Comment [A19]: 398-1247 m a.s.l

Response: There is no significant correlation between elevation and fallow replicates of species composition in Ukhrul and Chandel. Therefore, this question is not applicable 

Comment [A20]: Is there any similarity among the environmental factors for this sites?

Response: Environmental factors namely slope and aspect were found to be similar between 5 and 10 years fallow in Ukhrul as well as between 15 and 20 years fallow in Chandel. This explains for the high species similarity between the fallows (see S1 File).

Comment [A21]: The fact that the index incorporates both components of biodiversity can be seen as both a strength and a weakness. It is a strength because it provides a simple, synthetic summary, but it is a weakness because it makes it difficult to compare communities that differ greatly in richness. Since evenness and dominance are simply two sides of the same coin, their measures are complimentary. Simpsonʼs index is based on the probability of any two individuals drawn at random from an infinitely large community belonging to the same species.

See Morris et al 2014 Ecology and Evolution 2014; 4(18): 3514– 3524Choosing and using diversity indices: insights for ecological applications from the German Biodiversity Exploratories.

“Magurran and Dornelas (2010) argue against using compound indices when the objective is to detect effects of external factors on diversity, such as when assessing anthropogenic impacts on the environment. There is some empirical evidence that simple indices are indeed more effective in these cases”

Response: Since evenness and dominance are simply two sides of the same coin, the evenness index has been removed from the table to simplify the result interpretation. 

Comment [A22]: “evenness represents the degree to which individuals are split among species with low values indicating that one or a few species dominate, and high values indicating that relatively equal numbers of individuals belong to each species. Evenness is not calculated independently, but rather is derived from compound diversity measures such as H’, D1, and D2, as they inherently contain richness and evenness components. However, evenness as calculated from H’ (J’) is of only limited use predictively because it mathematically correlates with H’ (DeBenedictis 1973). E, calculated from D2 (Table 1), is mathematically independent of D1 (Smith and Wilson 1996) and therefore a more useful measure of evenness in many contexts”

Morris et al 2014 Ecology and Evolution 2014; 4(18): 3514– 3524Choosing and using diversity indices: insights for ecological applications from the German Biodiversity Exploratories

“The greater ability of measures derived from Hill’s N2 (D1, D2) to discriminate

Response: Since evenness and dominance are simply two sides of the same coin, the evenness index has been removed from the table to simplify the result interpretation

Comment [A23]: E.g. the Simpson's Dominance Index is telling you that this community (dominated by one specie, but containing even amounts of 9 other species), can be similar to a community with 3 dominant species and not much else. Does this prove anything? Not really -- just because the math works out does not mean that nature has to operate that way.

Response: Data mining to identify an index providing strong significant effects is avoided and the results are presented in the simplest manner.

Comment [A24]: Could the authors provide a distribution of species in undisturbed sites along the slope aspect and altitude gradients?

Response: The study sites ie the age of fallow stands were traced using satellite data. By performing post-classification comparison, the age of the fallow stand was ascertained. And it would be time consuming effort to trace back to a primary forest/undisturbed sites. Therefore, the findings of Waikom et al. (2017) on sacred groves of Manipur was used as reference for carbon stock recovery. 

Comment [A25]: We identified a total of 12 sites (four fallow aged plots x three replicates) in each district. How was the slope aspect and elevation for the three replicates? And how that distribution influences the results? It is difficult to check from Fig 1.

Response: The detailed descriptions of the sites are presented in table 1. The influences of site distribution are explained in the manuscript (see S1).

Comment [A26]: How were the differences for the replicates?

Response: It was observed that in Ukhrul, significant difference (F=16.71, P<0.001 and F=8.707, P<0.005) was observed in the 15 years fallow replicates of species richness and stem density as well as in the 10 years fallow replicates of Stem density (F=4.244, P<0.05). On the other hand, Chandel species richness was found to be significant among the replicates of 15 years fallow (F=3.912, P<0.05) and none of the replicates were significant for stem density.

Comment [A27]: Jhum

Response: The typographical error has been corrected to ‘Jhum’

Comment [A28]: Generalists species

Response: The sentence has been reframed as ‘The CCA triplots further showed that these species (ElaeFlor and CastHyst) were generalist species, that is, they appear opposite to the direction of increasing value of the environmental variables and its relation with the tree species’.

Comment [A29]: Can the authors provide the number of species that had biomass estimated from Brown 1997 equation? What was the DBH range?

Response: The number of species that had biomass estimated from Nath et al. (2019) and FSI volume equation has been mentioned. Species with dbh<5 cm were considered juvenile while the biomass for trees with dbh>5 cm were calculated using either Nath et al. (2019) or FSI volume equation.

Comment [A30]: What’s the uncertainty measure for this estimates?

Response: The uncertainty % of TLWBC decreased from 84% to 9% in Ukhrul and 60% to 12% in Chandel with increasing fallow age

Comment [A31]: That can be explained by the range of altitude for each Jhum fallow, the 5 year sites for UKHRUL 995-1285. 10 year 1179 – 1537. 15 year 1216 – 1404. 20 year 1225 – 1491. That bring my attention for human activity in lower altitudes during last decades. For Chandel site this trend didn’t occur. Extracted from the Excel file in supplementary material.

Response: Although slope and elevation displayed a positive correlation with the carbon stock in Ukhrul and Chandel respectively, it did not influence the recovery of carbon stock in the Jhum fallows.

Comment [A32]: The authors bring no information related to undisturbed forests. It’s difficult to talk about secondary successive communities without “primary communities” or old-growth forests.

Response: The study sites ie the age of fallow stands were traced using satellite data. By performing post-classification comparison, the age of the fallow stand was ascertained. And it would be time consuming effort to trace back to a primary forest/undisturbed sites. Therefore, the findings of Waikom et al. (2017) on sacred groves of Manipur was used as reference for carbon stock recovery.

Comment [A33]: What level of uncertainty for the use of wood density? Values for standard error of estimates for the different models/equations used were not clear.

Response: These sentences have been removed from the manuscript.

Comment [A34]: IPCC suggests the use of Brown 1997 equation when no data availability for the region 2000-4000 mm annual precipitation. IPCC do not prescribe anything.

Response: These sentences have been removed from the manuscript.

Comment [A35]: Not clear.

Response: These sentences have been removed from the manuscript.

Comment [A36]: Values for old growth forests not presented

 Response: Biomass carbon stock from old growth forest/undisturbed forest was used as a control to calculate per cent recovery of the jhum fallows.

Comment [A37]: Replications were not represented by all topographical factors

Response: Topographical parameters of the replicates are presented in Table 1.

Comment [A38]: Along the main text, figures etc check all the Jhum along the manuscript

Response: All the typographical error in the spelling of Jhum have been corrected.

Comment [A39]: Typo

Response: Fernside has been corrected as ‘Fearnside’

Comment [A40]: Typo

Response: coversion has been corrected as ‘conversion’

I hope, with the above changes, the manuscript may now be acceptable for publication in PLos One.

However, we will still be ready to do any further corrections, if desired.

With kind regards,

Sincerely,

Professor U.K.Sahoo

Attachment Submitted filename: Response_to_Reviewers.docx Click here for additional data file.

# Peer review: Decision Letter 1

**DOI:** 10.1371/journal.pone.0239906.r003

**Authors:** Gerhard Hartwig Buck-Sorlin

12 Aug 2020

PONE-D-19-29248R1

PATTERN OF FOREST RECOVERY AND CARBON STOCK FOLLOWING SHIFTING CULTIVATION IN MANIPUR, NORTH-EAST INDIA

PLOS ONE

Dear Dr. Sahoo,

Thank you for submitting your manuscript to PLOS ONE. After careful consideration, we feel that it has merit but does not fully meet PLOS ONE’s publication criteria as it currently stands. Therefore, we invite you to submit a revised version of the manuscript that addresses the points raised during the review process.

Your manuscript needs to undergo a second, minor revision along the lines suggested by the reviewer. Please follow closely the suggestions and resubmit the improved version as soon as possible. I have looked at the manuscript myself and agree with the points raised by the reviewer.

Please submit your revised manuscript by Sep 26 2020 11:59PM. If you will need more time than this to complete your revisions, please reply to this message or contact the journal office at plosone@plos.org. When you're ready to submit your revision, log on to https://www.editorialmanager.com/pone/ and select the 'Submissions Needing Revision' folder to locate your manuscript file.

Please include the following items when submitting your revised manuscript:

- A rebuttal letter that responds to each point raised by the academic editor and reviewer(s). You should upload this letter as a separate file labeled 'Response to Reviewers'.
- A marked-up copy of your manuscript that highlights changes made to the original version. You should upload this as a separate file labeled 'Revised Manuscript with Track Changes'.
- An unmarked version of your revised paper without tracked changes. You should upload this as a separate file labeled 'Manuscript'.

If you would like to make changes to your financial disclosure, please include your updated statement in your cover letter. Guidelines for resubmitting your figure files are available below the reviewer comments at the end of this letter.

If applicable, we recommend that you deposit your laboratory protocols in protocols.io to enhance the reproducibility of your results. Protocols.io assigns your protocol its own identifier (DOI) so that it can be cited independently in the future. For instructions see: http://journals.plos.org/plosone/s/submission-guidelines#loc-laboratory-protocols

We look forward to receiving your revised manuscript.

Kind regards,

Gerhard Hartwig Buck-Sorlin, Ph.D.

Academic Editor

PLOS ONE

[Note: HTML markup is below. Please do not edit.]

Reviewers' comments:

Reviewer's Responses to Questions

**Comments to the Author**

1. If the authors have adequately addressed your comments raised in a previous round of review and you feel that this manuscript is now acceptable for publication, you may indicate that here to bypass the “Comments to the Author” section, enter your conflict of interest statement in the “Confidential to Editor” section, and submit your "Accept" recommendation.

Reviewer #1: All comments have been addressed

**********

2. Is the manuscript technically sound, and do the data support the conclusions?

The manuscript must describe a technically sound piece of scientific research with data that supports the conclusions. Experiments must have been conducted rigorously, with appropriate controls, replication, and sample sizes. The conclusions must be drawn appropriately based on the data presented. 

Reviewer #1: Partly

**********

3. Has the statistical analysis been performed appropriately and rigorously? 

Reviewer #1: No

**********

4. Have the authors made all data underlying the findings in their manuscript fully available?

The PLOS Data policy requires authors to make all data underlying the findings described in their manuscript fully available without restriction, with rare exception (please refer to the Data Availability Statement in the manuscript PDF file). The data should be provided as part of the manuscript or its supporting information, or deposited to a public repository. For example, in addition to summary statistics, the data points behind means, medians and variance measures should be available. If there are restrictions on publicly sharing data—e.g. participant privacy or use of data from a third party—those must be specified.

Reviewer #1: Yes

**********

5. Is the manuscript presented in an intelligible fashion and written in standard English?

PLOS ONE does not copyedit accepted manuscripts, so the language in submitted articles must be clear, correct, and unambiguous. Any typographical or grammatical errors should be corrected at revision, so please note any specific errors here.

Reviewer #1: Yes

**********

6. Review Comments to the Author

Please use the space provided to explain your answers to the questions above. You may also include additional comments for the author, including concerns about dual publication, research ethics, or publication ethics. (Please upload your review as an attachment if it exceeds 20,000 characters)

Reviewer #1: Most comments were proper adressed and the manuscript have improved, but there is a main issue related to the interpretation of biomass and carbon stock values uncertainty and the use of Waikhom et al 2017 data for comparison of carbon stock recovery estimation. A central point after the selection of a allometric equation to apply to your data is understand if this equation gives the fresh biomass estimates or the dry biomass estimates. If the model gave you fresh biomass estimates you should first remove the water content (multipling the estimated value by 1 less water content percentage value) and then convert it to carbon stocks. Waikhom et al 2017 using Chambers et al 2001 equation found the aboveground biomass, carbon stock of the sacred grove ranged from 962.94 to 1130.79 Mg ha-1 and 481.47 to 565.40 Mg ha-1 C. Then Waikhon et al 2017 calculated the aboveground carbon stock by assuming that carbon content is 50% of the total aboveground biomass. And thats why the values reported are so high, e.g for Central Amazon old growth forests, above ground fresh biomass can assume values around 600 Mg.ha-1 and water content around 40.8%, so dry biomass will be the result of 600*(1-0.408)= 355,2 Mg.ha-1 this value assuming carbon content is 50% will rang around 177.6 Mg. ha-1 C.

Chambers et al assumed 38% of water content.

Lines 162-163. The findings of Waikhom et al. [34] on sacred groves of Manipur were used as comparison data for carbon stock recovery estimation.

When i first read tought tha Waikhom et al 2017 developed some allometric model, but instead they use a Central Amazon model developed by Chambers et al 2001.

Jeffrey Q. Chambers worked with 315 trees harvest by the group of Niro Higuchi, i strongly recommend the read of

Higuchi N, Suwa R, Higuchi FG, Lima AJN, Santos J dos, Noguchi H, et al. Overview of

Forest Carbon Stocks Study in Amazonas State, Brazil. Interactions Between Biosphere,

Atmosphere and Human Land Use in the Amazon Basin. Nagy L., Forsberg B., Artaxo P.

(eds); 2016. pp. 171–187. doi:10.1007/978-3-662-49902-3

ABSTRACT

"Tropical forests, specially the Amazon, are important ecosystems in research on the effects of climate change on biological systems and their functioning. The main relationship between the forest and the climate is the carbon fluxes and stock. Although carbon stock estimates of tropical forests are an issue and still raise many uncertainties. This study aims to present the main literature references and the huge effort to sample the forests of Central Amazon. Many attempts to estimate the carbon stock of the Amazon rainforest resulted in high uncertainties due to sampling and allometric constrains. After developing an allometric equation, based on destructive method and adapted to each different site based on dominant height, sampling over 1800 plots in primary and selectively logged non-flooded forests, across the State of Amazonas, the estimated average of total (tree’s above- and below-ground) carbon stock is 159.8 ± 9.2 Mg C ha−1."

**********

7. PLOS authors have the option to publish the peer review history of their article (what does this mean?). If published, this will include your full peer review and any attached files.

If you choose “no”, your identity will remain anonymous but your review may still be made public.

**Do you want your identity to be public for this peer review?** For information about this choice, including consent withdrawal, please see our Privacy Policy.

Reviewer #1: No

[NOTE: If reviewer comments were submitted as an attachment file, they will be attached to this email and accessible via the submission site. Please log into your account, locate the manuscript record, and check for the action link "View Attachments". If this link does not appear, there are no attachment files.]

While revising your submission, please upload your figure files to the Preflight Analysis and Conversion Engine (PACE) digital diagnostic tool, https://pacev2.apexcovantage.com/. PACE helps ensure that figures meet PLOS requirements. To use PACE, you must first register as a user. Registration is free. Then, login and navigate to the UPLOAD tab, where you will find detailed instructions on how to use the tool. If you encounter any issues or have any questions when using PACE, please email PLOS at figures@plos.org. Please note that Supporting Information files do not need this step.

# Author comment: Author response to Decision Letter 1

**DOI:** 10.1371/journal.pone.0239906.r004

12 Sep 2020

Respected Gerhard Hartwig Buck-Sorlin, Ph.D.

Academic Editor, PLOS ONE

Thank you very much for giving us an opportunity to re-revise our manuscript (PONE-D-19-29248) entitled “PATTERN OF FOREST RECOVERY AND CARBON STOCK FOLLOWING SHIFTING CULTIVATION IN MANIPUR, NORTH-EAST INDIA” and considering it for minor revision.

I wish to mention that we have thoroughly gone through the comments, and have revised the manuscript accordingly. We immensely thank the anonymous reviewer for his critical comments and inputs which has further improved the revised manuscript.

The point-wise response to the reviewer’s comment is as follows.

Reviewer #1: Most comments were proper addressed and the manuscript have improved, but there is a main issue related to the interpretation of biomass and carbon stock values uncertainty and the use of Waikhom et al 2017 data for comparison of carbon stock recovery estimation. A central point after the selection of a allometric equation to apply to your data is understand if this equation gives the fresh biomass estimates or the dry biomass estimates. If the model gave you fresh biomass estimates you should first remove the water content (multipling the estimated value by 1 less water content percentage value) and then convert it to carbon stocks. Waikhom et al 2017 using Chambers et al 2001 equation found the aboveground biomass, carbon stock of the sacred grove ranged from 962.94 to 1130.79 Mg ha-1 and 481.47 to 565.40 Mg ha-1 C. Then Waikhon et al 2017 calculated the aboveground carbon stock by assuming that carbon content is 50% of the total aboveground biomass. And thats why the values reported are so high, e.g for Central Amazon old growth forests, above ground fresh biomass can assume values around 600 Mg.ha-1 and water content around 40.8%, so dry biomass will be the result of 600*(1-0.408)= 355,2 Mg.ha-1 this value assuming carbon content is 50% will rang around 177.6 Mg. ha-1 C.

Chambers et al assumed 38% of water content.

Lines 162-163. The findings of Waikhom et al. [34] on sacred groves of Manipur were used as comparison data for carbon stock recovery estimation.

When i first read tought tha Waikhom et al 2017 developed some allometric model, but instead they use a Central Amazon model developed by Chambers et al 2001.

Jeffrey Q. Chambers worked with 315 trees harvest by the group of Niro Higuchi, i strongly recommend the read of Higuchi N, Suwa R, Higuchi FG, Lima AJN, Santos J dos, Noguchi H, et al. Overview of Forest Carbon Stocks Study in Amazonas State, Brazil. Interactions Between Biosphere, Atmosphere and Human Land Use in the Amazon Basin. Nagy L., Forsberg B., Artaxo P.(eds); 2016. pp. 171–187. doi:10.1007/978-3-662-49902-3

ABSTRACT

"Tropical forests, specially the Amazon, are important ecosystems in research on the effects of climate change on biological systems and their functioning. The main relationship between the forest and the climate is the carbon fluxes and stock. Although carbon stock estimates of tropical forests are an issue and still raise many uncertainties. This study aims to present the main literature references and the huge effort to sample the forests of Central Amazon. Many attempts to estimate the carbon stock of the Amazon rainforest resulted in high uncertainties due to sampling and allometric constrains. After developing an allometric equation, based on destructive method and adapted to each different site based on dominant height, sampling over 1800 plots in primary and selectively logged non-flooded forests, across the State of Amazonas, the estimated average of total (tree’s above- and below-ground) carbon stock is 159.8 ± 9.2 Mg C ha−1."

Response: The uncertainty of TLWBC was evaluated as per Higuchi et al. 2016. In the present study, the biomass was estimated based on species specific volume equation as well as region specific allometric model. Conversion from tree volume to carbon content results in larger uncertainties as compared to the use of allometric models [Njana et al. 2017]. This explains the high uncertainty percentage in the present study.

As for the use of Waikhom et al 2017 data for comparison of carbon stock recovery estimation, they estimated the biomass using regression equation developed by Chambers et al. which had 38% moisture content. Therefore, biomass estimated from the sacred grove was converted to dry biomass by removing the water content and then the carbon stock was determined. This was done to assess the recovery per cent of our estimated dry biomass carbon.

I hope, with the above changes, the manuscript may now be accepted for publication in PLos One.

Thanking you once again and with kind regards,

Sincerely,

Professor U.K.Sahoo

Attachment Submitted filename: Response to Reviewers.docx Click here for additional data file.

# Editor report: Decision Letter 2

**DOI:** 10.1371/journal.pone.0239906.r005

**Authors:** Gerhard Hartwig Buck-Sorlin

16 Sep 2020

PATTERN OF FOREST RECOVERY AND CARBON STOCK FOLLOWING SHIFTING CULTIVATION IN MANIPUR, NORTH-EAST INDIA

PONE-D-19-29248R2

Dear Dr. Sahoo,

We’re pleased to inform you that your manuscript has been judged scientifically suitable for publication and will be formally accepted for publication once it meets all outstanding technical requirements.

Within one week, you’ll receive an e-mail detailing the required amendments. When these have been addressed, you’ll receive a formal acceptance letter and your manuscript will be scheduled for publication.

An invoice for payment will follow shortly after the formal acceptance. To ensure an efficient process, please log into Editorial Manager at http://www.editorialmanager.com/pone/, click the 'Update My Information' link at the top of the page, and double check that your user information is up-to-date. If you have any billing related questions, please contact our Author Billing department directly at authorbilling@plos.org.

If your institution or institutions have a press office, please notify them about your upcoming paper to help maximize its impact. If they’ll be preparing press materials, please inform our press team as soon as possible -- no later than 48 hours after receiving the formal acceptance. Your manuscript will remain under strict press embargo until 2 pm Eastern Time on the date of publication. For more information, please contact onepress@plos.org.

Kind regards,

Gerhard Hartwig Buck-Sorlin, Ph.D.

Academic Editor

PLOS ONE

Additional Editor Comments (optional):

I think you have now addressed all points raised by the reviewer. Your manuscript can now be accepted, but after looking through the manuscript myself I have some final remarks that you should carefully consider :

- L. 212 "Table 2." Insert a space after the dot

- p. 12 etc: eliminate the footnotes, transform them into figure legends (e.g. Figs; 6 - 8)

- L. 273 etc. I saw that you frequently use colons (:) at the end of chapter titles: These need to be deleted

- When referring to supplementary material, do this by writing "see supplementary figure S1", not "file"

- avoid splitting tables, if unavoidable insert a header saying "Table 6 (contd.)"

- Acknowledgments: I think the hard work of the anonymous reviewer should be acknowledged

- Fig. 1: I see a problem of poor resolution for the middle part of the figure, and the left part of the figure (map of India) seems distorted (I DID look at the tiff version!)

- S1 File: The table requires a legend, at least explaining "JF5, JF10, JF15...", and I think "fellows" must be "fallows".

Reviewers' comments:

# Editor report: Acceptance letter

**DOI:** 10.1371/journal.pone.0239906.r006

**Authors:** Gerhard Hartwig Buck-Sorlin

28 Sep 2020

PONE-D-19-29248R2 

PATTERN OF FOREST RECOVERY AND CARBON STOCK FOLLOWING SHIFTING CULTIVATION IN MANIPUR, NORTH-EAST INDIA 

Dear Dr. Sahoo:

I'm pleased to inform you that your manuscript has been deemed suitable for publication in PLOS ONE. Congratulations! Your manuscript is now with our production department. 

If your institution or institutions have a press office, please let them know about your upcoming paper now to help maximize its impact. If they'll be preparing press materials, please inform our press team within the next 48 hours. Your manuscript will remain under strict press embargo until 2 pm Eastern Time on the date of publication. For more information please contact onepress@plos.org.

If we can help with anything else, please email us at plosone@plos.org. 

Thank you for submitting your work to PLOS ONE and supporting open access. 

Kind regards, 

PLOS ONE Editorial Office Staff

on behalf of

Prof. Dr. Gerhard Hartwig Buck-Sorlin 

Academic Editor

PLOS ONE
